# Quantifying differences in cell line population dynamics using CellPD

**DOI:** 10.1186/s12918-016-0337-5

**Published:** 2016-09-21

**Authors:** Edwin F. Juarez, Roy Lau, Samuel H. Friedman, Ahmadreza Ghaffarizadeh, Edmond Jonckheere, David B. Agus, Shannon M. Mumenthaler, Paul Macklin

**Affiliations:** 1 Lawrence J. Ellison Institute for Transformative Medicine, University of Southern California, Los Angeles, California USA; 2 Department of Electrical Engineering, Viterbi School of Engineering, University of Southern California, Los Angeles, California USA

**Keywords:** Phenotype digitizer, Growth rate, Net birth rate, Phenotype comparison, Cell population dynamics, Parameter estimation, Computational modeling, Mathematical models, Open source, User friendly, MultiCellDS

## Abstract

**Background:**

The increased availability of high-throughput datasets has revealed a need for reproducible and accessible analyses which can quantitatively relate molecular changes to phenotypic behavior. Existing tools for quantitative analysis generally require expert knowledge.

**Results:**

CellPD (cell phenotype digitizer) facilitates quantitative phenotype analysis, allowing users to fit mathematical models of cell population dynamics without specialized training. CellPD requires one input (a spreadsheet) and generates multiple outputs including parameter estimation reports, high-quality plots, and minable XML files. We validated CellPD’s estimates by comparing it with a previously published tool (cellGrowth) and with Microsoft Excel’s built-in functions. CellPD correctly estimates the net growth rate of cell cultures and is more robust to data sparsity than cellGrowth. When we tested CellPD’s usability, biologists (without training in computational modeling) ran CellPD correctly on sample data within 30 min. To demonstrate CellPD’s ability to aid in the analysis of high throughput data, we created a synthetic high content screening (HCS) data set, where a simulated cell line is exposed to two hypothetical drug compounds at several doses. CellPD correctly estimates the drug-dependent birth, death, and net growth rates. Furthermore, CellPD’s estimates quantify and distinguish between the cytostatic and cytotoxic effects of both drugs—analyses that cannot readily be performed with spreadsheet software such as Microsoft Excel or without specialized computational expertise and programming environments.

**Conclusions:**

CellPD is an open source tool that can be used by scientists (with or without a background in computational or mathematical modeling) to quantify key aspects of cell phenotypes (such as cell cycle and death parameters). Early applications of CellPD may include drug effect quantification, functional analysis of gene knockout experiments, data quality control, minable big data generation, and integration of biological data with computational models.

**Electronic supplementary material:**

The online version of this article (doi:10.1186/s12918-016-0337-5) contains supplementary material, which is available to authorized users.

## Background

The growing adoption of systems biology and high-throughput experimental techniques increasingly demonstrates the need for quantitative and dynamic measurements to better characterize the complexity of biological systems [1, 2]. Measurements from a single experimental snapshot in time (e.g., an endpoint analysis) can often be misleading. For example, cell growth dynamics are influenced by cell density and nutrient availability, which are continually in flux. It is therefore better to use data across multiple time points when measuring cell phenotypes. On a broader scale, dynamical measurements can help to compare data across labs and identify protocol errors/discrepancies that may go unnoticed if data are only collected at a single time point.

With the increasing availability of high-throughput microscopy and high content screening (HCS), one can measure cell counts in many different environmental contexts with high precision over several time points [3]. These platforms can be used to link studies on molecular biology to observable, quantitative changes in cell behavior [4, 5]. However, as these experimental platforms have advanced, they have allowed the generation of vast amounts of data which, in turn, require sophisticated bioinformatics tools for analysis [6]. In order to leverage these bioinformatics tools, a scientist needs to learn how to use complex computer programs [7] or work in bioinformatics-oriented programming environments (e.g., R, MATLAB, or Python), often without the benefit of graphical interfaces to assist them [8]. The need for specialized knowledge is compounded when the user, for example, may want to test several mathematical models of cell population dynamics (e.g., birth, death, and clearance rates) and choose one among them. Furthermore, the many ways in which a software package, the operating system in which it runs, and its required dependencies can be configured lead to challenges in data reproducibility [9–11]. While built-in functions in popular spreadsheet software such as Microsoft Excel can perform basic analyses on small, simple datasets (e.g., total cell counts for a few replicates), the functions cannot easily be extended to fit more sophisticated mathematical models that are better suited to analyzing more complex datasets. Furthermore, in the process of implementing more sophisticated mathematical models, a scientist can inadvertently introduce elusive bugs in their calculations [12, 13]. Therefore, tools which facilitate the replication and implementation of new analyses should be used in scientific computing.

To expedite the quantitative analysis of cellular phenotypes from experimental data while promoting data reproducibility, we introduce CellPD: a user-friendly cell line phenotype digitizer which obtains best-fit parameters and uncertainty estimates for cell birth, death, and population carrying capacity, based upon well-established “canonical” mathematical forms (e.g., exponential and logistic growth, with either net birth rates or separate birth, death, and dead cell clearance rates). CellPD has been designed to comply with the MultiCellDS data standard [14], therefore it can be expanded to record additional phenotype parameters, such as pharmacodynamics and cell motility. CellPD uses Microsoft Excel-compatible spreadsheets containing cell counts and experimental metadata as its sole input. The spreadsheets are also compatible with open source office suites such as LibreOffice. It is packaged as both a Python script (for those with existing Python 3 or Python 2.7 installations) and standalone executables for Windows and OSX, eliminating the need for installing and learning any additional software. Finally, CellPD generates locally-stored webpage outputs to clearly and intuitively present parameter estimation results with publication-quality tables and graphics as well as machine-readable XML outputs. These webpage reports also rank the quality of each fitted model to help the user choose the appropriate results without specialized mathematical knowledge. (See Additional file 1 for two examples of CellPD outputs.) CellPD is a beneficial tool for experimentalists, especially for those without a computational background or an existing partnership with a trained biostatistician or mathematician, as it provides a uniform and precise method for analyzing cellular dynamics data. Furthermore, CellPD not only computes growth rates from time series data, but also fits mathematical models in order to gain further insights from time series data, such as discerning between cytostatic and cytotoxic drug effects (as shown in the Results and Discussion section). While all the tasks that CellPD performs (automatic analysis by multiple models, uncertainty quantification, automatic ranking of fitted models by quality, user-friendly interfaces, publication-quality graphics, open data standards-compliant outputs for future data sharing, and utility in high-content screening experiments) are in principle possible today with significant custom scripts (e.g., in R, Python, or Matlab), no tools available today have already been tailored to these tasks and shared with the scientific community in a user-friendly format. In this article we describe some applications of CellPD and link to its source code (which will be updated as new features are added) so the scientific community can use it and build upon it.

## Implementation

CellPD was designed with the following goals in mind:

- *Utility for experimental biologists:* The main goal of CellPD is to facilitate a quantitative description and analysis of cell population dynamics, using mathematical models that are powerful enough to make full use of increasingly detailed datasets.
- *Ease of learning and ease of use:* A scientist with no training in mathematical/computational modeling can learn how to use CellPD in an hour or less.
- *Robustness to sparsity in data:* CellPD can fit mathematical models to irregularly and sparsely sampled data requiring a minimum of two data points to fit the most basic mathematical model.
- *Accessibility and Shareability:* CellPD is open source and free to use with an unrestrictive license.
- *Extensibility:* We have planned extensions to CellPD’s capabilities. In addition, its source code may be modified by any member of the scientific community, provided they follow the guidelines of the (permissive) MIT License.
- *Portability:* CellPD’s Python code is packaged with a Python interpreter and all the required libraries; therefore, a computer running Windows, OSX, or Linux can run it without installing any software.

### Previous work

There have been numerous efforts to compare and standardize cell line data across labs to ensure reproducibility and accuracy [15–17]. For example, an early effort by Osborne et al. characterized MCF7 breast cancer cells grown in four different laboratories [18]. Their investigation exposed substantial differences in the four labs’ cell population doubling times. However, it can be difficult to discern any irregularities between cell cultures from different labs using doubling times for comparison, especially if those doubling times have not been computed to account for confluency effects.

Many tools have been developed specifically to estimate cell line growth parameters. Several were written in R such as cellGrowth [4, 19], grofit [20], and minpack.lm [21, 22]; MATLAB packages include PHANTAST [23] and SBaddon [24]; Ruby packages include BGFit [25]; and Python packages include ABC-SysBio [26] and GATHODE [27]. Although these packages are very useful, they are difficult to use for those without formal programming or bioinformatics expertise; moreover, the MATLAB-based packages require additional, costly software licenses. Some of these packages require data to be formatted in an inflexible format, for example requiring the data to be the output of a specific high content screening microscope. None of these tools and software packages are designed for regular use by scientists without extensive training with computational tools (i.e., they do not incorporate user-friendly inputs and outputs). They are also primarily designed for single-lab use. For example, they create outputs with lab-specific formats, rather than a standardized, well-annotated format suitable for curation and meta-analysis. These output formats make it challenging to compare different datasets from multiple laboratories. Thus, they do not answer the call for (big) data sharing [28]. While spreadsheet software such as Microsoft Excel can be used for some basic calculations (such as computing the net growth rate of an exponentially growing cell culture), fitting more sophisticated models is much more difficult and potentially error-prone. Hand-coded spreadsheet formulas and macros can hide subtle but critical errors (e.g., incorrect row/cell numbers), sometimes invalidating results or requiring paper retractions [12, 13].

CellPD aims to fill these workflow gaps by providing a user-friendly tool to estimate some key cell phenotype parameters from data acquired using common experimental platforms. In this paper, we lay the groundwork for a suite of tools that can be shared among different labs, that will help to facilitate data sharing and cross-lab meta-analyses. While the first release of CellPD is focused on quantifying cell cycle and death parameters, it has been built from the ground up to allow future extensions to quantify other phenotypic parameters, such as motility and pharmacodynamics, and to leverage anticipated advances in single-cell tracking to test hypotheses-driven phenotype parameters (e.g., an S-phase duration that depends upon glucose availability and cell size). Some early applications of CellPD may include quantification of drug effects on cancer cells (using data from assays containing varying drug doses), functional analysis of gene and other knockout experiments (such as the ones used in Gagneur et al. [4]), quantifying the effect of the microenvironment on cell phenotype (such as described in Garvey et al. [5]), cell culture quality control (by comparing estimated growth rates with a curated database), data mining (by extracting data from databases and analyzing it with CellPD) and generation of minable big data (by creating digital cell lines for each experiment that CellPD analyzes), and integration of biological data into computational models (by using CellPD’s estimates as parameters for computational models).

In order to simplify the user interface, the primary input for CellPD is a Microsoft Excel spreadsheet that contains the experimental data (e.g., total cell counts at different time points), metadata related to the experimental setup (e.g., the name of the cell line and user notes), and the user information (e.g., the name and contact information of the CellPD user/data creator). Every CellPD download includes template spreadsheets to guide users through the spreadsheet layout and data formatting. In order to bring mathematical modelling expertise to biologists, the user-supplied data are then parsed and used to calibrate several mathematical models (for example exponential and logistic growth); the models were designed for extracting biologically-meaningful cell parameters from typical experimental data, such as the cell population growth rate and cell cycle information (such as population doubling times) with adjustment for confluence effects. See the Methods section for details on the mathematical models, their underlying assumptions, and a layperson's description of the mathematical models.

CellPD automatically selects one or more mathematical models for fitting based upon the type and quantity of data supplied by the user. For example, if the user provides cell counts at different time points but no cell viability, then CellPD (without extra input from the user) will calibrate models which describe cell counts but will omit models which describe cell death. Finally, a series of locally-stored webpage outputs are created to report and rank the fitted models (by quality of fit) and their parameters. Each fitted model includes a layperson’s description of the underlying model assumptions and the biological meaning of each parameter. The results are reported in publication-quality figure (PNG, JPG, SVG, EPS, and PDF files) and table formats (XLSX and CSV files). For a list of all the outputs, see the Additional file 1.

**Fig. 1:**
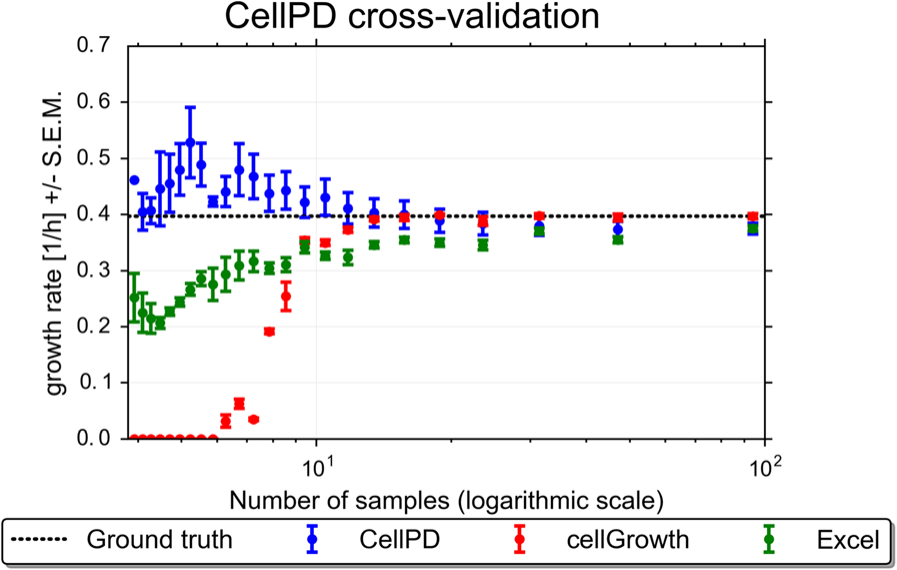
Cross-validation of growth rates. Growth rates ± Standard error of the mean (SEM) of Yeast strain seg_07A grown in YPD media computed by cellGrowth (*red*), Excel (*green*) and CellPD (*blue*) using different number of sampling time points (i.e., at different sampling rates). All three tools correctly estimated the maximum growth rate for high sampling rates. For low sampling numbers (approximately less than 10 samples), the three tools become less accurate; cellGrowth lacks the necessary number of data points to perform data smoothing, Excel becomes inaccurate, but CellPD continues to estimate reasonable growth rates. Even at the limit case of only 3 sampled data points, CellPD provides a reasonable estimate (although it can no longer estimate SEM of the parameter)

CellPD has been designed to run on both finely-sampled data (measured at many time points, for example every 15 min, such as in yeast and bacteria experiments where dynamics have shorter timescales) and more sparsely (measured once per day, as is common in cancer cell culture experiments). We intend CellPD to be used in a wide range of applications so it is robust to sparse data, but its accuracy improves when given more data points (as shown in Fig. 1). For a model with *n* parameters, CellPD requires at least *n* data points to estimate those parameters and at least *n* + 1 data points to estimate standard error of the mean for each parameter. The simplest model that CellPD can fit is the exponential model (a two-parameter model; see the methods section for more details); CellPD can analyze data so as long as there are at least 2 data points, in which case CellPD will assume exponential growth in between those two points.

### Downloading CellPD

CellPD is hosted at SourceForge.net. Its source code, Windows and OSX executables can be downloaded at http://CellPD.sf.net [29]. Alternatively, tutorials and the most recent version of CellPD can also be downloaded at http://MultiCellDS.org/CellPD/ [30].

## Results and discussion

We first validate CellPD’s parameter estimates by comparing its results against another previously published cell growth parameter estimator, cellGrowth, and Excel 2016’s built in functions (see Additional file 2 for a list of other tools that we examined). After validating the code, we demonstrate some key applications of CellPD by (1) evaluating its use in cell culture quality control by comparing two cultures of the same cell line (HCT 116, a colon cancer epithelial-like cell line) grown in two different media, (2) demonstrating its utility in HCS experiments by calculating dose-dependent cell birth and death parameters in a simulated drug screening experiment, and (3) using CellPD to determine whether these drugs are cytostatic or cytotoxic.

To test and quantify user-friendliness, we subjected CellPD, cellGrowth, and Excel to a series of timed use cases: installing all necessary software (first time setup for a new user), formatting data (6 replicates of yeast growth data from [4, 19] sampled every 15 min) running the software, and analyzing output. The lead author performed this test and recorded them (see Table 1 for links to the videos), and a group of 12 biologists volunteered to perform this test using CellPD either as individual testers (two participants) or in either pairs or groups of three. We also tested robustness by repeating the use cases for sparser data samples.

**Table 1:** Comparison between CellPD, cellGrowth, and Excel

	CellPD	cellGrowth	Spreadsheet (Excel)
0.25 h sampling rate (95 samples) max_growth_rate (±SEM) h^-1^	0.375 (±0.00963)	0.3971 (±0.0045)	0.3751 (±0.0045)
3 h sampling rate (7 samples) max_growth_rate (±SEM) h^-1^	0.438 (±0.0326)	0.1916 (±0.0045)	0.3044 (±0.0096)
6 h sampling rate (3 samples) max_growth_rate (±SEM) h^-1^	0.462 (N/A)	Breaks down	0.2519 (±0.0435)
Usability benchmark: T_t__otal_, lead author Usability benchmark: T_total_, lead author	6 m 55 s	6 m 35 s	2 m 27 s
Usability benchmark: T_analysis_, lead author Usability benchmark: T_analysis_, lead author	5 m 43 s	3 m 17 s	2 m 27 s
Usability benchmark: T_total_, 12 scientists unfamiliar with CellPD	Approximate range, in minutes [20, 30]	N/A	N/A
Usability benchmark: T_analysis_, 12 scientists unfamiliar with CellPD	Approximate range, in minutes [14, 26]	N/A	N/A
Run time	~30 s	<1 s	<1 s
Video of timed use case	https://youtu.be/3xR9x_2pBKs	https://youtu.be/DO-LkVVglIg	https://youtu.be/YCyCfzl7yFY
Comments on ease of use	Tutorial available, drag and drop option	Good tutorial to use custom data	Present on (viritually) every computer, many tutorials available online
Comments on input user interface	Executable file, command line option	Command-line in R	Manual input of formulas within the GUI
Comments on output user interface	Easy to read webpages with downloadable plots	Option to display and save an informative plot	Easy to create simple graphs
Typical UI	https://youtu.be/3xR9x_2pBKs?t=276	https://youtu.be/DO-LkVVglIg?t=272	https://youtu.be/YCyCfzl7yFY?t=12
Typical output	https://youtu.be/3xR9x_2pBKs?t=406	https://youtu.be/DO-LkVVglIg?t=391	https://youtu.be/YCyCfzl7yFY?t=158
Feature comparison matrix:			
Uncertainty quantification	Yes	If user computes it	If user computes it
Parametric growth models	Yes	Yes	If user creates them
Nonparametric growth models	No	Yes	If user creates them
Publication quality graphs	Yes	No	No
Fully annotated results in a standardized markup language	Yes	No	No
Open Source	Yes	Yes	No
Language written	Python	R	C/C++, C++/Java/Python
Required software to run	Spreadsheet editor (Excel, LibreOffice), Web browser (internet Explorer will suffice)	R	Excel, LibreOffice
Required computational expertise	No specialized experience	Working knowledge of R	Familiarity with spreadsheets

All three tools correctly estimate the growth rate when provided with a large number of samples. cellGrowth is more precise than CellPD for higher number of samples (i.e., shorter sampling intervals). However, even with fewer samples (i.e., larger sampling intervals), CellPD correctly estimates the growth rate (within the 95 % confidence interval). For fewer samples (i.e., larger sampling intervals), both cellGrowth and Excel become unreliable. CellPD is slower than cellGrowth or Excel for an experienced user, but CellPD does not require prior programming knowledge (unlike cellGrowth) and it also creates multiple useful outputs (Excel does not generate publication-quality graphs and cellGrowth has the option of creating a single graph which the user can export). CellPD is quicker to set up than cellGrowth, but it takes longer to run in order to create the multiple outputs. Excel usually requires no set up (beyond installing Microsoft Office), and it is often already installed in a research computer. The lead author computed the usability benchmark running a fixed, “clean” Windows 7 configuration on a Virtual Machine (VM). This VM included an installation of LibreOffice 5.1.4 and was run in a Lenovo ThinkPad Yoga with an Intel Core i7-4600U CPU with 8GB of Ram running Windows 10 (64-bit)

### CellPD comparison and cross-validation

#### Advantages of CellPD over Excel’s built-in fitting functions

Some of the computations that CellPD performs automatically can be replicated, albeit not automatically, using a spreadsheet. Spreadsheet software can use built-in regression functions to fit an exponential curve to experimental data. While other, more complex, dynamics can be modeled using a spreadsheet (such as logistic curves), these approaches push the limits of spreadsheet software by requiring hand-coded formulas, macros, or VisualBasic coding. Such calculations tend to be less reusable and more error prone, occasionally invalidating study results or even contributing to retractions [12, 13]. Hence, spreadsheets should only be used for minor calculations; more complex applications are best left to well-designed, purpose-built open source scientific software. CellPD is designed to estimate parameters accounting for expected behavior in cell population growth (such as logistic limitations). Additionally, CellPD is modular and extensible, thus allowing the user to fit multiple mathematical models at once, modify its current models, or even code new custom models. CellPD also creates high resolution figures which can be used in scientific publications, as well as minable XML reports and intuitive webpage reports.

#### Cross-validating CellPD

In order to cross-validate CellPD’s parameter estimates, we utilized a publicly-available dataset from [4]. From this dataset, we selected the strain *seg_07A* grown in “YPD” medium (high in glucose) and computed the population growth rates using CellPD, Excel’s linear regression function (linest), and cellGrowth. Not all of the replicates from that dataset have the same number of measurements, so we truncated the longer-time replicates so that they all have the same number of time points. We first computed the maximum growth rate of the data using all three tools, and defined cellGrowth’s estimate (0.397 h^−1^_,_ which corresponds to a population doubling time of 1.75 h) as the ground truth (the true value). We then used the three tools to estimate the growth rate using only a subset of the data (to simulate an experiment where samples are taken less frequently). We first fitted to the original data, which corresponds to 95 samples (and a sampling time interval of 0.25 h), then we reduced the number of samples roughly by half (the equivalent of sampling every 0.5 h), then we used roughly 1/3rd of the number of samples (the equivalent of sampling every 0.75 h), and so on, until we fitted to only 3 data points (i.e., sampling every 6 h). Figure 1 shows the estimated growth rates as the number of samples is varied (for the same experiment) using the three tools (see Additional file 3 for a figure where the x-axis represents sampling interval). CellPD, Excel, and cellGrowth correctly estimate the maximum growth rate for largest number of samples. CellPD generates reasonable growth parameter estimates over a broad range of data sampling rates, while both Excel and cellGrowth rapidly lose accuracy as the number of samples decreases. In particular, cellGrowth fails altogether when there are fewer than 6 samples. cellGrowth relies on smoothing methods arising from signal processing methods in order to provide accurate growth rates. Thus, it requires a substantial number of data samples. Neither CellPD nor Excel perform data smoothing, so they can estimate parameters with fewer data samples. While Excel can still compute the net growth rate with very few sampling points, its approach is prone to user bias (because users must choose which points to include in the linear regression and which to omit, e.g., due to confluence effects) and replication errors due to the manual nature of this computation. Hence there is a need for tools like CellPD which perform these analyses systematically and automatically. However, even tools which do not perform data smoothing are susceptible to problems caused by low number of data samples. Figure 1 shows that the uncertainty of the parameter estimates reduced for all three tools as the number of samples is increased, as described in Harris et al. [31], two data points are not enough to accurately model the dynamics of cell population growth.

### Usability comparison testing

In order to quantitatively assess the usability of software package we used the following usability benchmark:

#### Usability benchmark

Measures how easily a new user can set up the package, starting from a “clean slate” using data formatted as outputted by a generic high content screening microscope (i.e., raw cell counts or optical densities at different times, each replicate recorded in its own file).

Use case:

- Step 1: Install and setup software (included required dependencies)
- Step 2: Reformat data for the software
- Step 3: Run software
- Step 4: Compute means and standard deviations of maximum growth rate

Total time (T_total_) = Step 1 to Step 4.

T_analysis_ = Step 3 and Step 4.

We recorded times measured by the usability benchmark as rows of a feature comparison matrix described in Table 1. We also recorded videos while the lead author performed the usability benchmark. Links to the videos are also listed in Table 1. To minimize user experience differences between the methods, the lead author spent time learning R and cellGrowth before performing the benchmark tests. Thus, the times reported are the minimum times to perform an analysis. For a novice user with no computational expertise, the differences would be larger. In particular, such users would require at least 1–10 days to learn introductory R before completing the benchmark use case, whereas users can complete the benchmark use case with CellPD without any additional training. The usability benchmark was repeated for CellPD by multiple volunteers with a wide range of computational experience, these times are also recorded in Table 1. The volunteers did not repeat the benchmarks on cellGrowth because using it requires familiarity with R.

### Using CellPD to compare two cultures of the same cell line

**Fig. 2:**
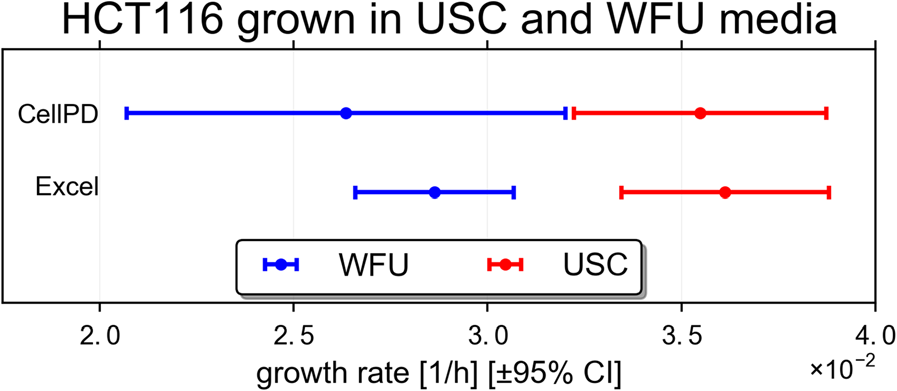
Using CellPD and excel to identify difference in “single clone cell lines” grown under the same microenvironmental conditions. Growth rates of HCT116 cell cultures grown in two different media (*red*: USC, Blue: WFU). USC cells grow at a rate of 0.0354 ± 0.0017 h^−1^, 95 % CI [0.0322, 0.0388]h^−1^ as estimaded by CellPD. WFU cells grow at a rate of 0.0264 ± 0.0029 h^−1^, 95 % CI [0.0206, 0.0321]h^−1^ as measured for CellPD. The 95 % CIs do no overlap (using either tool), showing that the cell cultures grow at different rates. For the complete CellPD outputs see Additional file 1

A significant issue in biological experimentation is inter- and intra-laboratory variability [10, 15–17, 32]. For example, cells are typically grown in various media irrespective of the initial culturing methods. Moreover, even when culturing conditions are standardized, the use of biological reagents that are inherently variable in composition (such as fetal bovine serum (FBS)) can dramatically impact cell growth [33]. As result, it is important to devise tools such as CellPD to assess cell growth and perform quantitative quality control. We used CellPD and Excel (there are not enough sample points for cellGrowth to process these data) to compute the growth rate of two HCT116 cultures grown in two different media. In this paper, cells grown in McCoy’s media are labeled “USC” and those grown in DMEM are labeled “WFU”. Figure 2 shows the growth rates and the 95 % confidence interval (CI) as computed by CellPD and Excel. With either tool, the 95 % CI of the same HCT116 cells grown using these two different media do not overlap. This experiment was designed to observe different growth dynamics when culturing the same cell line (HCT116) in two different media due to differences in glucose concentrations (McCoy’s – 16 mM; DMEM – 25 mM) and other nutrients and growth factors [33]. CellPD allows for detection and quantification of such differences. These quantifications can be used to identify potential deviations from the protocol, such as in this case (where we intentionally used the wrong medium). This result highlights the importance of standardizing experimental conditions within and across laboratories. Such a large discrepancy in growth rates as a result of culture media could significantly alter the interpretation of standard tumor cell growth and their response to stimuli or inhibitors, such as chemotherapies.

### Using CellPD to analyze (synthetic) high-content drug screening data

**Fig. 3:**
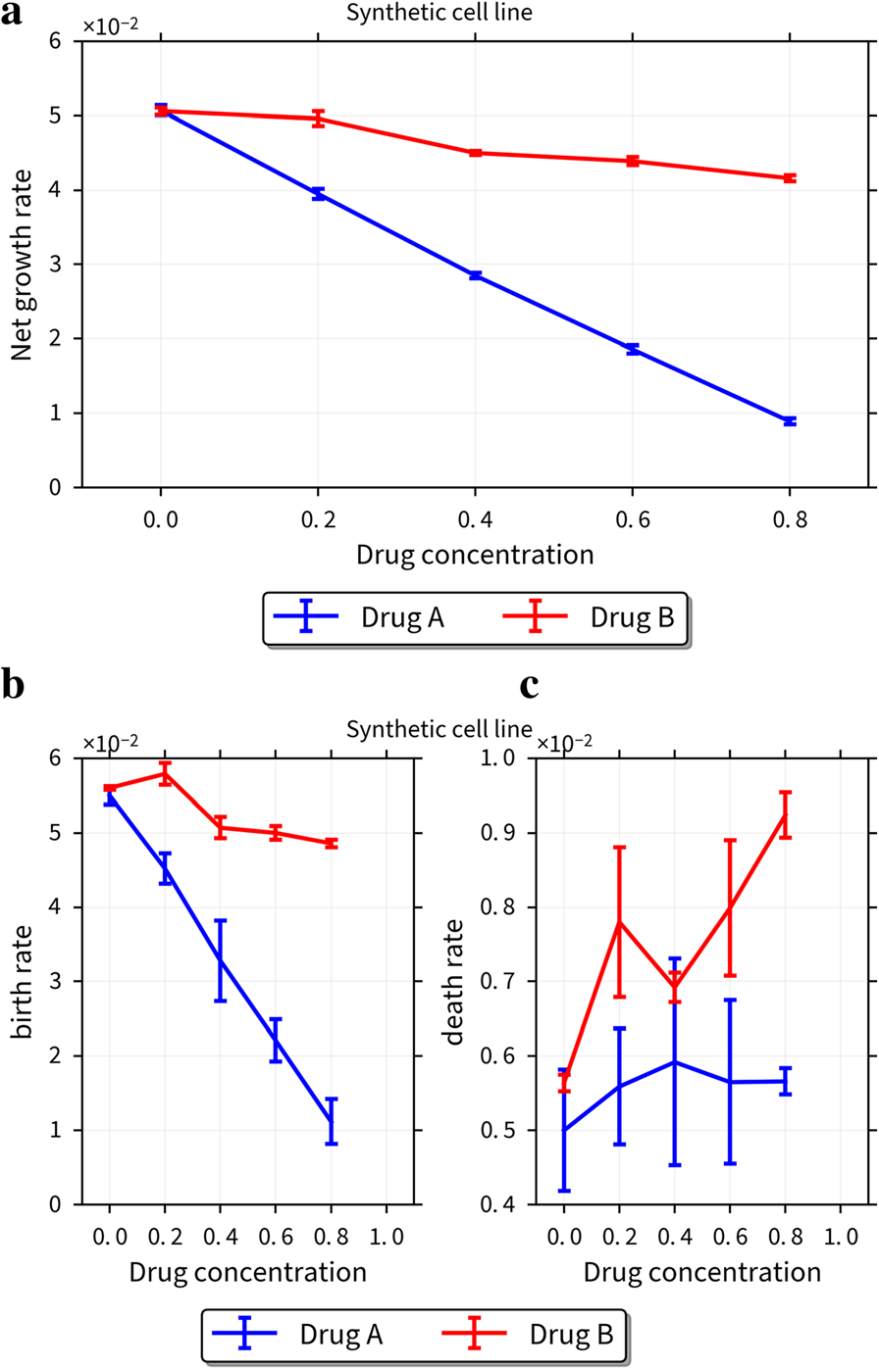
Using CellPD to analyze high-content drug screening (synthetic) data. CellPD’s quantification of a synthetic cell line’s response to two different drugs. The net growth rates **a** show different responses to each drug. CellPD automatically decouples the birth rate **b** and death rate **c** which elucidates the drugs’ mechanisms of action. Drug A reduces the birth rate while keeping the death rate relatively constant, Drug B mainly increases the death rate

CellPD can be used to quantitate cell phenotype under multiple drug conditions using data generated in high content screening platforms. To demonstrate this feature, we first generated a synthetic drug screening dataset typical of high-content screening platforms and tested CellPD against these synthetic data. Specifically, we generated synthetic live and dead cell counts for two drugs at 5 doses, with 5 biological replicates for each experimental condition (a specific drug at a specific dose). Each simulated experimental measurement included normally-distributed noise to approximate both instrument and biological variability. See Additional files 4 and 5 for full details on the synthetic dataset, generating code, and the synthetic data themselves. CellPD was able to quantitate the net birth rate for each experimental condition, along with uncertainty estimates, and plot the responses. See Fig. 3a. Note that these analyses would be difficult to automate with cellGrowth, and would require substantial manual effort when using Excel.

We note that because we used a synthetic dataset with known “true” values, we can assess CellPD’s accuracy and to test its robustness to measurement noise. We found that even with 10 % noise, CellPD was able to recover the correct parameter values for both simulated drugs at all doses, with a mean error of 2.6 % for the birth rate, 6.7 % for the death rate, and 3.0 % for the net growth rate. See Additional files 4 and 5 for more details.

### Using CellPD to differentiate between cytotoxic and cytostatic drug effects

Continuing with the prior analysis, we used CellPD to separately quantitate the cell birth and death rate parameters for each experimental condition. See Fig. 3b-c. This additional analysis correctly reproduced the known birth and death rate parameters. Moreover, CellPD found that drug A was primarily cytostatic (it mainly influenced the cell birth rate) and drug B was cytotoxic (it chiefly affected the cell death rate). Because cellGrowth and Excel only fit the total population curve (i.e., they determine the net birth rate = birth – death), they cannot easily repeat this analysis nor help distinguish between cytostatic and cytotoxic drug effects here. In fact, by only examining the dose-dependent net birth rate, it can be (wrongly) inferred that drug A is cytotoxic because increasing the dose of drug A rapidly decreases its net birth rate to negative values, whereas drug B has a very shallow dose-net birth rate curve, which could be (wrongly) interpreted as arresting cell birth to maintain a zero or small net birth rate. Here, a more detailed analysis (facilitated by CellPD) was necessary to discern that counter to intuition from the net birth rate graphs, drug A is cytostatic, and drug B is cytotoxic. More sophisticated mathematical analyses—made possible by the broader class of models encoded in CellPD with straightforward usability—are necessary to discern the mechanism of action of each simulated drug compound.

### Limitations

CellPD can currently only estimate parameters for predefined models (see Methods section for a list of the models); it does not currently support user-defined mathematical models except by directly modifying the source code. CellPD can directly analyze a dataset in which multiple environmental conditions are changing, e.g. if a dataset includes experiments where both the oxygenation and the media are changed independently. However, this functionality is under development. Currently publication-quality plots can only be generated for a single environmental condition with multiple values (e.g., different levels of oxygenation). When more than one environmental condition is changing at the same time, CellPD will perform the quantitative analysis and it will create plots, but those plots may not be as intuitive to read as the rest of the output files that CellPD generates. CellPD is designed to analyze population-level phenotypic data and is currently not equipped to provide single-cell dynamic information, although this is a feature that could be added in the future.

### Future versions of CellPD

CellPD is open source: anyone may modify the code under the terms of the MIT License (MIT). We plan to update CellPD and release future versions which will include:

- Implement other common mathematical models of cell growth (e.g., Gompertzian).
- Implement additional cell cycle models (e.g., cells transitioning between G_0_/G_1_, S, G_2_, and M phases) suitable to flow cytometry data.
- Automatically handle multiple-condition datasets for a single experimental factor (e.g., varying a drug level or the oxygenation). This function is in active development and testing. A beta version is now included with the main code of CellPD, with a fully-supported version anticipated soon.
- Automatically handle multiple-condition datasets for multiple experimental factors (e.g., varying a drug level and the oxygenation).
- Implement pharmacodynamics (drug response) models.
- Interactive web version.
- Interface with ORCID’s API [34] to pull in user details automatically.
- Black-box optimization, allowing the user to define a custom mathematical model and estimate its parameters.
- Alternative minimization techniques such as cross-validation, bootstrap, genetic algorithms, and different heuristics for global optimization.

## Conclusions

CellPD is a useful tool for biologists to analyze, quantify, and share phenotypic data. It can be used for data quality control and to identify unexpected changes in cell population dynamics. It can help automate analysis of high-content screening data, while distinguishing between cytostatic and cytotoxic drug effects. In all of its analyses, it makes use of biological and technical replicates to help assess uncertainty. CellPD facilitates integration of experimental data into computational models, rapidly quantifying critical phenotypic characteristics such as a cell line’s net birth rate and producing consistent publication quality data.

## Methods

### Cell culture and reagents

HCT116 cells were acquired from ATCC and maintained in McCoy’s media (USC HCT116). HCT116 cells cultured in DMEM media were also gifted to us by the Soker laboratory at Wake Forest University (WFU HCT116). All culture media was supplemented with 10 % fetal bovine serum (Gemini) and 1 % penicillin/streptomycin solution and cells were kept under standard tissue culture conditions (5 % CO_2_, 37 °C).

### Live/dead cell counts

Cells were seeded at 1,000 cells per well in 96 well plates (Corning #3904). Live and dead cell counts were determined at 0, 48, and 72 h using the Operetta high content screening (HCS) platform by PerkinElmer. Briefly, cells were stained with 5 μg/mL Hoechst 33342 (Invitrogen #H21492) and 7.5 nM Sytox Red (Life Technologies S34859) prior to imaging to identify cells as live or dead, respectively. Using the Harmony 3.5.2 (PerkinElmer) image analysis software, individual cells were quantified as live or dead using nuclear segmentation algorithms and intensity thresholds. Each assay condition was performed in triplicate. All data points used in the analysis were taken before any confluence effects were apparent. Raw data can be found in Additional file 6.

### Software implementation

CellPD is written in Python 3 (but a version compatible with Python 2.7 was created, in part, using 3to2), and it can be downloaded as source code to run in scripted python, or as a downloadable, self-standing executable (Windows and Mac). As shown in the graphical abstract, CellPD takes in a Microsoft Excel file as an input (or a compatible XLSX/XLS spreadsheet created or edited with open source software such as LibreOffice [35]). It creates a collection of webpage (HTML) reports as primary outputs, including a summary and ranking of fitted models and publication-quality graphics. CellPD also generates multiple supplementary outputs to facilitate data extraction. Supplementary outputs include: log-linear plots, black and white plots, figure captions, model descriptions, model equations in latex, table of estimated parameters in multiple formats (TEX, XLSX, and CSV), and a digital cell line XML file (a standardized, hierarchical reporting of cell phenotype and metadata; see the MultiCellDS project website for more details [14]). The HTML-based report is saved locally for later access.

In order to run properly, CellPD requires the following common Python libraries (note that they are included in the executable files):

- **LMFIT:** Non-Linear Least-Square Minimization and Curve-Fitting for Python. CellPD requires it to perform the minimization required in parameter estimation and to store the parameter values in LMFIT’s Parameter’s structure [36]. MIT license [37].
- **NumPy:** A basic numerical library for Python. CellPD requires it through to create numerical arrays and to use various mathematical functions [38]. BSD license [39].
- **SciPy:** A scientific computing library for Python. CellPD requires it to complement NumPy’s mathematical functions and for its numeric integration algorithms [40]. Custom BSD compatible license [41].
- **matplotlib:** A common and versatile plotting library for Python. CellPD requires it to generate publication quality plots [42]. Custom BSD compatible license: [43].
- **tzlocal:** A library with time and locale tools for Python. CellPD requires it for generating time-zone sensitive time stamp [44]. License CC0 1.0 Universal [44].
- **tabulate:** A library for handling tables in Python. CellPD requires it for generating table of parameters for the reports [45]. MIT License [45].
- **OpenPyXL:** A library to read/write Excel files in Python. CellPD requires it to read the input files and to create the excel files that are supplemental outputs [46]. MIT/Expat license: [46].
- **PyInstaller:** A software to create stand-alone executable files for Windows and Mac. CellPD does not invoke PyInstaller, rather, we use PyInstaller to package CellPD with Python interpreters into a single executable file [47]. Modified GPL license to “have no restrictions in using PyInstaller as-is” [47].
- **3to2:** A python script that converts most Python 3 code into Python 2. CellPD does not invoke 3to2, rather, we use 3to2 to translate most of CellPD’s Python 3 code into Python 2.7 code, the rest of the code that is not translated by 3to2 is translated manually [48]. Apache Software License [49].

### Further algorithmic detail

- **Levenberg–Marquardt algorithm (LMA):** CellPD uses LMFIT [50], which uses the LMA to minimizes an error metric to obtain an optimal, best fit between a supplied mathematical and data. LMA is a generalization of the steepest gradient descent method designed to solve smooth nonlinear problems (by using a “damping” on the gradient). A good explanation can be found in the book Numerical Recipes, The Art of Scientific Computing [51]. In our application of LMA, we used the following error metric:
  $$ \begin{array}{c}\hfill {\mathrm{Error}}_i={\mathrm{Data}}_i-{\mathrm{Simulation}}_i\hfill \\ {}\hfill \mathrm{WSSE}={\displaystyle \sum_i^N}\frac{1}{\sigma_i}\frac{{\left[{\mathrm{Error}}_i\right]}^2}{{\mathrm{Data}}_i}\hfill \end{array} $$
  where sigma is the standard deviation of the *i*^*t*h^ data point. Thus, data with the largest uncertainty carries the least weight in the optimization (i.e., LMA prioritizes data with higher certainty). WSEE is the Weighted Sum of Squared Errors.
- **MAPE and Reduced***χ*_*ν*_^2^**:** In order to compare different models, the Mean Absolute Percentage Error (MAPE) and the reduced chi squared are computing using the formulas:
  $$ \begin{array}{c}\hfill \mathrm{MAPE}=\frac{1}{N}{\displaystyle \sum_i^N}100\frac{\left|{\mathrm{Error}}_i\right|}{{\mathrm{Data}}_i}\hfill \\ {}\hfill {\chi}_{\nu}^2=\frac{\mathrm{WSSE}}{{\mathrm{N}}_{\mathrm{data}}-{\mathrm{N}}_{\mathrm{parameters}}}\hfill \end{array} $$
  where N_data_ is the number of data samples and N_parameters_ is the number of parameters of the model being evaluated. MAPE gives an intuitive sense of how well the model fit the individual data time points, on average, expressed as a percentage of the fitted data. *χ*_*ν*_^2^ adjusts this metric to account for the complexity of the fitted model (the difference between the number of measurements and the number of model parameters). It is meant to find a balance between fitting the data and simplifying the model, to avoid overfitting. (E.g., with enough parameters, a model could be made to fit every data point, even if it were a very poor model of the underlying biological system.) Both MAPE and *χ*_*ν*_^2^ are appropriate scores for ranking models applied to a given dataset so we provide both to allow the user decide which metric they prefer.
- **Estimates of standard error of the mean (SEM):** To estimate the SEM of the estimated parameter *i*, LMFIT uses the formula:
  $$ SE{M}_i=\sqrt{\chi_{\nu}^2Cov\left(i,i\right)} $$
  where *Cov*(*i*, *i*) is the element of the covariance matrix in the *i*^*t*h^ row and the *i*^*t*h^ column. A good explanation of these numerical methods can be found in LMFIT’s website [36, 50] and can be supplemented by [52].

### Mathematical models implemented

- **live:** This is an exponential model that describes the growth of the live cells:
  $$ \frac{d\left[\mathrm{Live}\right]}{dt}=\mathrm{growth}\_\mathrm{rate}\left[\mathrm{Live}\right] $$
  Here, [**Live]** is the total number of live cells, and **growth_rate** is the net rate of live cell population growth (cell birth minus death).
- **live_logistic:** This modifies the exponential growth model to account for logistic growth effects (e.g., depletion of a growth substrate, or approaching cell confluence):
  $$ \frac{d\left[\mathrm{Live}\right]}{dt}=\mathrm{growth}\_\mathrm{rate}\left(1-\frac{\left[\mathrm{Live}\right]}{L_{\mathrm{cap}}}\right)\left[\mathrm{Live}\right] $$
  **[Live]** is the total number of live cells, and **growth_rate** is the net rate of live cell population growth (cell birth minus death). ***L***_**cap**_ is the total cell population carrying capacity (the maximum number of live cells).
- **live_dead:** This extends the exponential model to describes the changes of the live and dead cell populations:
  $$ \begin{array}{rcl}\frac{d\left[\mathrm{Live}\right]}{dt}& =& \mathrm{birth}\_\mathrm{rate}\left[\mathrm{Live}\right]-\mathrm{death}\_\mathrm{rate}\left[\mathrm{Live}\right]\\ {}\frac{d\left[\mathrm{Dead}\right]}{dt}& =& \mathrm{death}\_\mathrm{rate}\left[\mathrm{Live}\right]-\mathrm{clearance}\_\mathrm{rate}\left[\mathrm{Dead}\right]\end{array} $$
  **[Live]** is the total number of live cells, **[Dead]** is the total number of dead cells, **birth_rate** is the cell birth rate, **death_rate** is the cell death rate, and **clearance_rate** is the rate at which dead cells are cleared from the system (or the rate at which they become undetectable/unrecognizable to cell segmentation, cell counter, or other measurement techniques). **1/birth_rate** is the mean time between cell divisions, and **1/clearance_rate** is the mean time required for dead cells to degrade and/or cease to be recognized as cells by cell detection software.
- **live_dead_logistic:** This model modifies the live_dead model to account for logistic population effects:
  $$ \begin{array}{rcl}\frac{d\left[\mathrm{Live}\right]}{dt}& =& \mathrm{birth}\_\mathrm{rate}\left(1-\frac{\left[\mathrm{Live}\right]}{L_{\mathrm{cap}}}\right)\left[\mathrm{Live}\right]-\mathrm{death}\_\mathrm{rate}\left[\mathrm{Live}\right]\\ {}\frac{d\left[\mathrm{Dead}\right]}{dt}& =& \mathrm{death}\_\mathrm{rate}\left[\mathrm{Live}\right]-\mathrm{clearance}\_\mathrm{rate}\left[\mathrm{Dead}\right]\end{array} $$
  **[Live]** is the total number of live cells, **[Dead]** is the total number of dead cells, **birth_rate** is the cell birth rate, **death_rate** is the cell death rate, and **clearance_rate** is the rate at which dead cells are cleared from the system (or the rate at which they become undetectable/unrecognizable to cell segmentation, cell counter, or other measurement techniques). ***L***_**cap**_ is the total cell population carrying capacity (the maximum number of live cells). **1/birth_rate** is the mean time between cell divisions, and **1/clearance_rate** is the mean time required for dead cells to degrade and/or cease to be recognized as cells by cell detection software.
- **total:** This is an exponential model that describes the growth of the live and dead cells combined:
  $$ \frac{d\left[\mathrm{Total}\right]}{dt}=\mathrm{growth}\_\mathrm{rate}\left[\mathrm{Total}\right] $$
  **[Total]** = **[Live]** + **[Dead]** is the total number of cells, and **growth_rate** is the net rate of cell population growth (cell birth minus death).
- **total_logistic:** This modifies the exponential growth model to account for logistic growth effects (e.g., depletion of a growth substrate, or approaching cell confluence) in the total cell population:
  $$ \frac{d\left[\mathrm{Total}\right]}{dt}=\mathrm{growth}\_\mathrm{rate}\left(1-\frac{\left[\mathrm{Total}\right]}{T_{\mathrm{cap}}}\right)\left[\mathrm{Total}\right] $$
  **[Total]** = **[Live]** + **[Dead]** is the total number of cells, and **growth_rate** is the net rate of cell population growth (cell birth minus death). *T*_cap_ is the total cell population carrying capacity (the maximum number of total cells).

## Additional files

Additional file 1: Example of CellPD’s outputs. This folder contains two examples of the outputs generated by CellPD (using the data from Fig. 2 and Additional file 6). (ZIP 36462 kb) Additional file 2: Other tools that we examined. This file contains a list of five other tools we attempted to use and the reasons why we did not use them. (PDF 120 kb) Additional file 3: Growth rate estimates versus sampling frequency. This file contains an alternative representation of the same data from Fig. 1. In this representation, the x-axis is the sampling interval (instead of the log 10 of the number of samples). (PDF 239 kb) Additional file 4: Creation of synthetic data. This file contains a description of how the synthetic data for Fig. 3 was created, as well as the calculation of the estimation error. (PDF 500 kb) Additional file 5: Synthetic data. This folder contains the synthetic data used for Fig. 3 and Additional file 4. Additionally, it contains the python scripts that were used to make the synthetic data, Fig. 3, and Additional file 4: Figure S9-1. (ZIP 890 kb) Additional file 6: Raw data. This spreadsheet contains the raw data used for Fig. 2, as well as the experimental setup. (XLSX 15 kb) Additional file 7: CellPD Tutorial. This file contains a step by step tutorial detailing how to add experimental data and metadata to CellPD’s input file, as well as how to run CellPD in Windows, OSX, or Linux. (PDF 2240 kb) Additional file 8: Cuantificación de Dinámicas Poblacionales de Cultivos de Líneas Celulares Utilizando CellPD. This file is a Spanish summary of the manuscript. (PDF 127 kb) Additional file 9: Source code. This folder contains the Python code with the version of CellPD used throughout this manuscript. (ZIP 554 kb) Additional file 10: Windows and OSX distributions of CellPD. This file contains simple instructions and links to download CellPD and use it on a Windows or an OSX computer. (PDF 119 kb)

## Abbreviations

CellPD: Cell phenotype digitizer
CI: Confidence interval
FBS: Fetal bovine serum
HCS: High content screening
LMA: Levenberg-Marquardt algorithm
MAPE: Mean absolute percentage error
SEM: Standard error of the mean
VM: Virtual machine
WSSE: Weighted sum of squared errors
