# Supplementary material for: Quantifying differences in cell line population dynamics using CellPD
Source: BMC Syst Biol. 2016 Sep 21;10:92. doi: 10.1186/s12918-016-0337-5 (PMC5031291; doi:10.1186/s12918-016-0337-5)
Supplement: Additional file 1: — Example of CellPD’s outputs. This folder contains two examples of the outputs generated by CellPD (using the data from Fig. 2 and Additional file 6). (ZIP 36462 kb) [file 12918_2016_337_MOESM1_ESM.zip › WFU/output/total_logistic/total_logistic_report.html]

CellPD: Cell Line Phenotype Digitizer Report


# Fitting results: Total cells model (with logistic limits)

## MultiCellDS model: total\_logistic

## HCT116 cell line

These are the results from running the CellPD: Cell Line Phenotype Digitizer (Version 1.0) for the Total cells model (with logistic limits). Please cite this tool as

> We fit the data to several growth models using CellPD (Version 1.0), which uses least-squares fitting ... (Juarez et al., 2015).
>
> E. Juarez et al., *CellPD: a cell line parameter digitizer to quantify and record critical cell line growth parameters*, eLife (2016, in review)

---

## Model used

## Model description

This is an exponential model that describes the growth of the live and dead cells combined accounting for logistic limitations.

---

## Fitted Parameters

| Parameter | Value | Std. Error | units | Inv\_value |
| --- | --- | --- | --- | --- |
| growth\_rate | 0.0264 | inf | 1/hours | 37.9 |
| carrying\_capacity | 1.66e+10 | inf | number of cells | 6.03e-11 |
| seeding\_cells | 911 | inf | number of cells | 0.0011 |

### Derived parameters

Corrected exponential doubling time: 26.3 hours

Rough doubling time [ignoring logistic limitations and post-thaw-lags]: 23.5 hours


**Table 1** shows the list of parameters that achieve minimal Sum of Squared Errors (SSE) for the Total cells model (with logistic limits), MultiCellDS name: 'total\_logistic.' Sum of Squared Errors is 4.59, Mean Absolute Percentage Error is 9.32%,and the Reduced Chi Squared Goodness of Fit is inf.

**Warning:** At least one parameter estimate standard error is of the same order of magnitude as its corresponding parameter
.This model may not be appropiate.

## Model fit

**Fig. 1:** Shows the GoF of the Total cells model (with logistic limits),MultiCellDS name: 'total\_logistic.' Sum of Squared Errors (SSE) is 4.59, the Mean Absolute Percentage Error (MAPE) is 9.32%,and the Reduced Chi-Squared Goodness of Fit (GoF) is inf.  
[Download as PNG]
[Download as SVG]
[Download as PNG (black & white)]
[Download as SVG (black & white)]

---

[Back to top]

CellPD: Cell Line Phenotype Digitizer (Version 1.0) - Results

[Back to the main page]
