# Supplementary figures and images for: Quantifying differences in cell line population dynamics using CellPD
Source: BMC Syst Biol. 2016 Sep 21;10:92. doi: 10.1186/s12918-016-0337-5 (PMC5031291; doi:10.1186/s12918-016-0337-5)

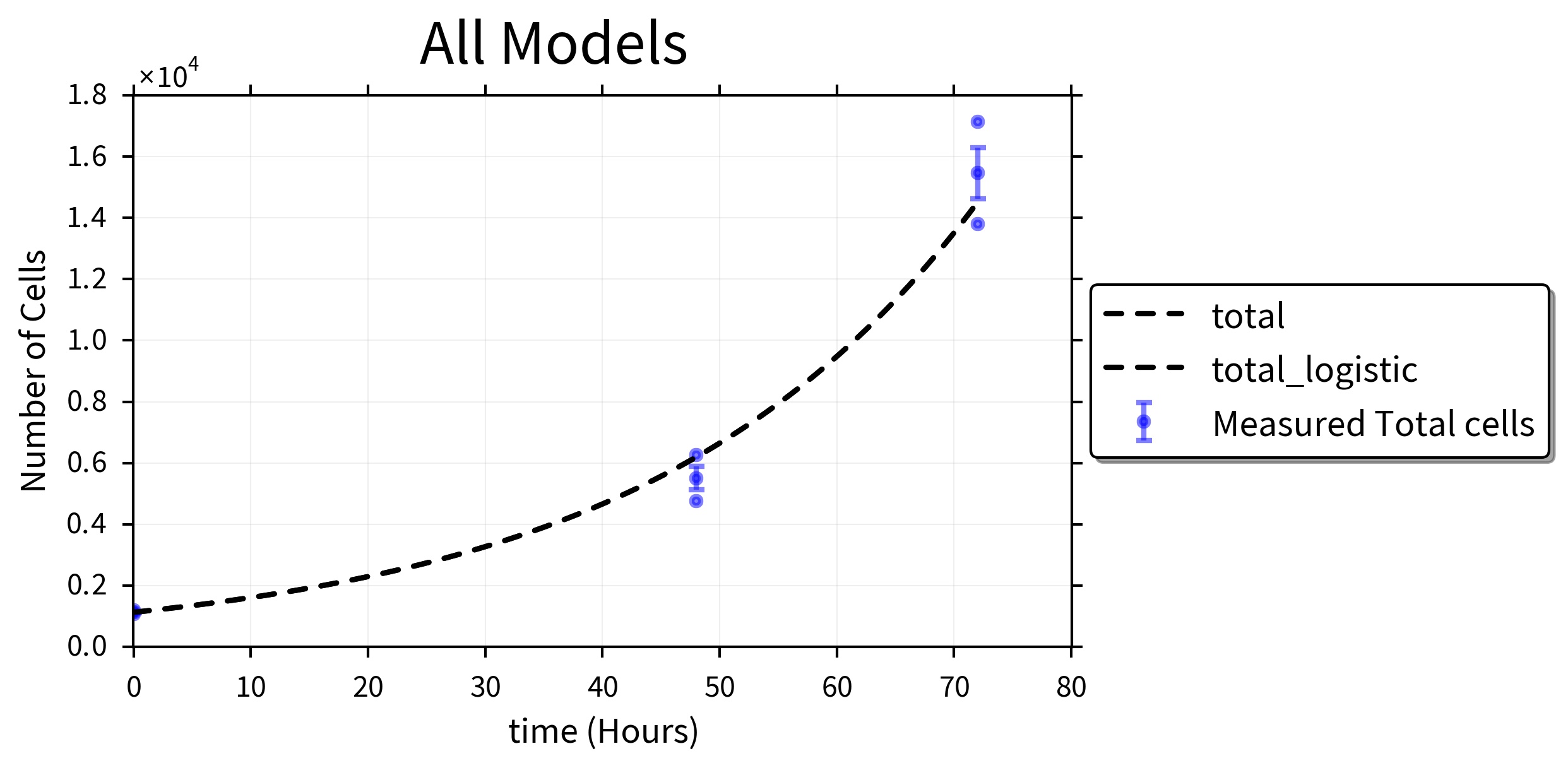

Supplement: Additional file 1: — Example of CellPD’s outputs. This folder contains two examples of the outputs generated by CellPD (using the data from Fig. 2 and Additional file 6). (ZIP 36462 kb) [file 12918_2016_337_MOESM1_ESM.zip › USC/output/data/All_Models.jpg]

# All Models

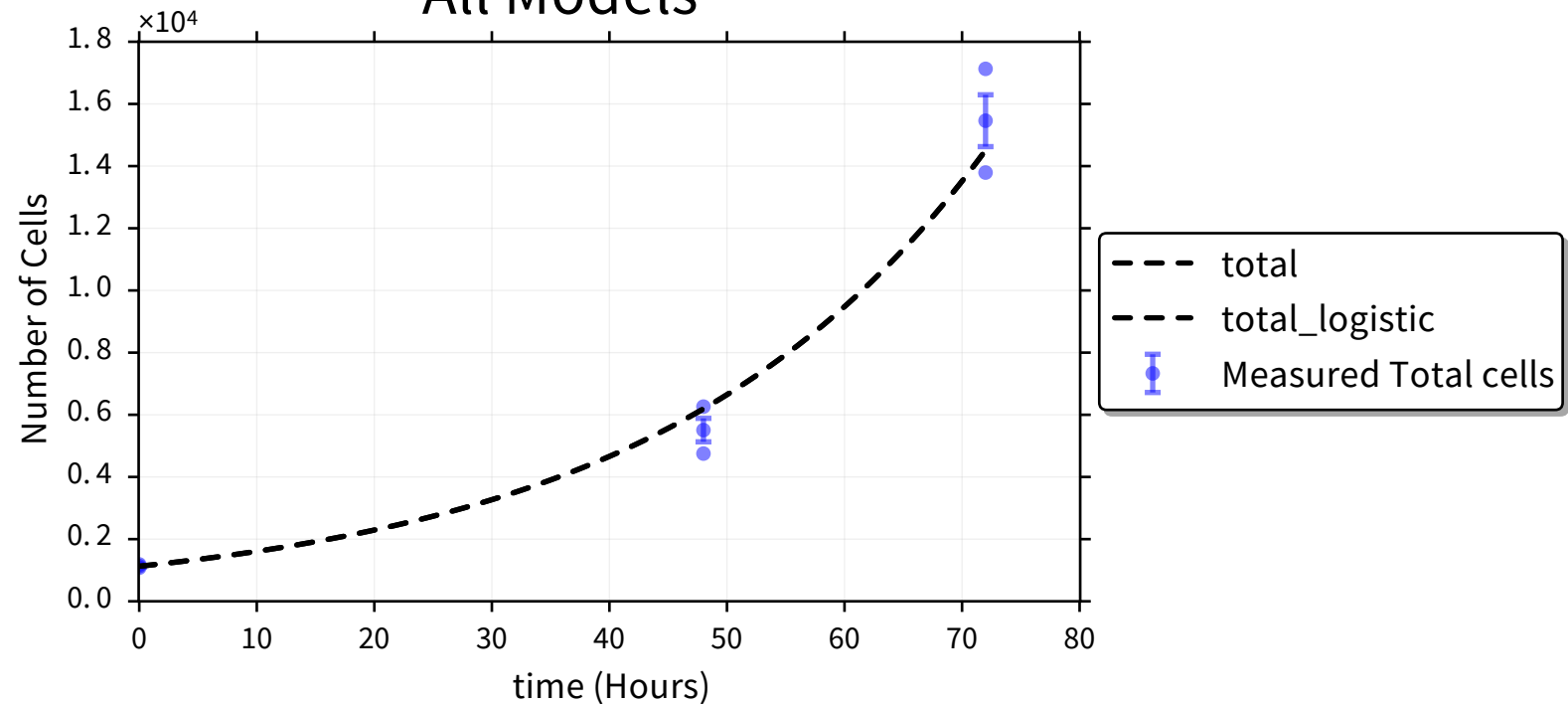

Supplement: Additional file 1: — Example of CellPD’s outputs. This folder contains two examples of the outputs generated by CellPD (using the data from Fig. 2 and Additional file 6). (ZIP 36462 kb) [file 12918_2016_337_MOESM1_ESM.zip › USC/output/data/All_Models.pdf]

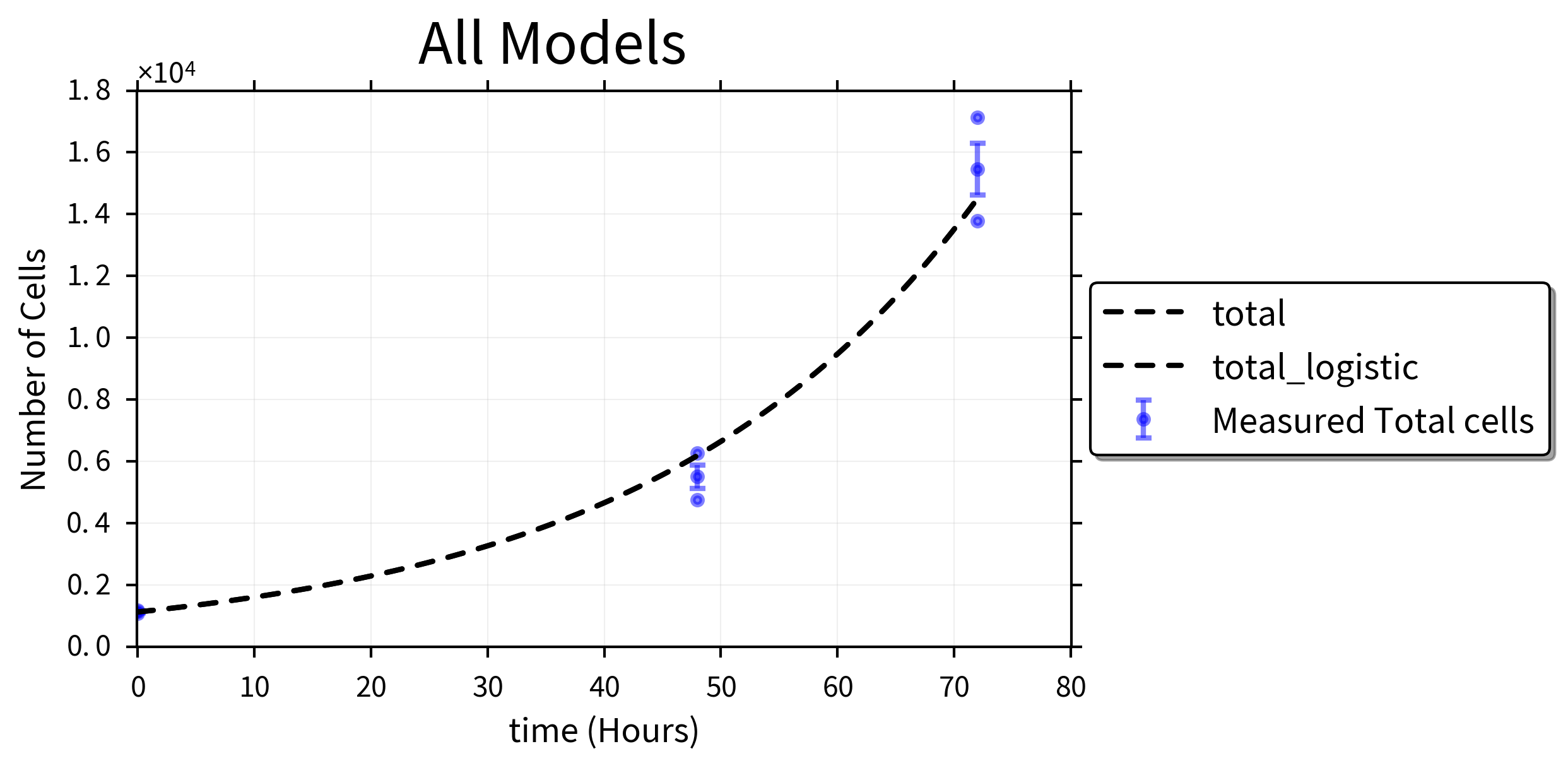

Supplement: Additional file 1: — Example of CellPD’s outputs. This folder contains two examples of the outputs generated by CellPD (using the data from Fig. 2 and Additional file 6). (ZIP 36462 kb) [file 12918_2016_337_MOESM1_ESM.zip › USC/output/data/All_Models.png]

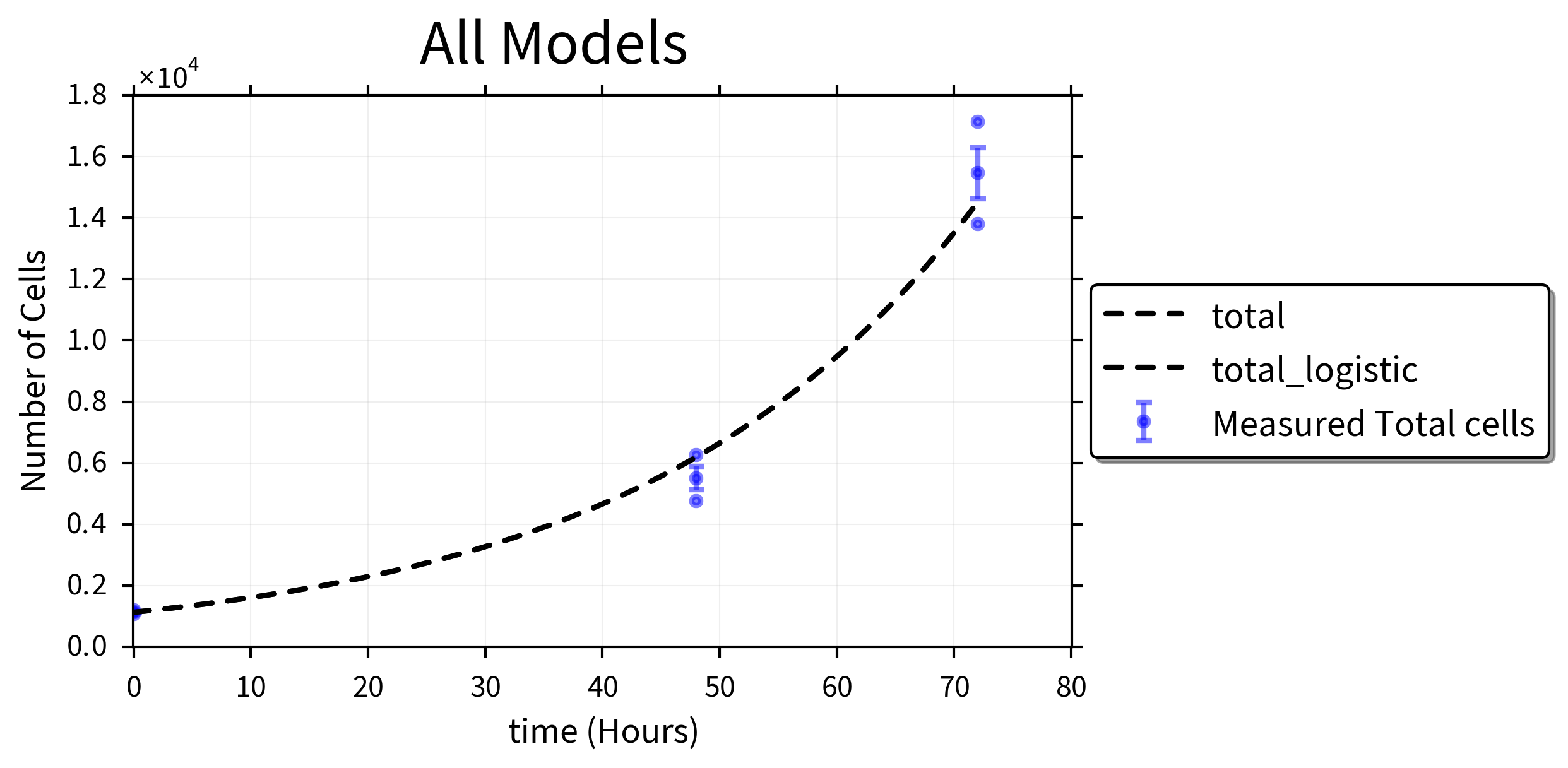

Supplement: Additional file 1: — Example of CellPD’s outputs. This folder contains two examples of the outputs generated by CellPD (using the data from Fig. 2 and Additional file 6). (ZIP 36462 kb) [file 12918_2016_337_MOESM1_ESM.zip › USC/output/data/All_Models.tiff]

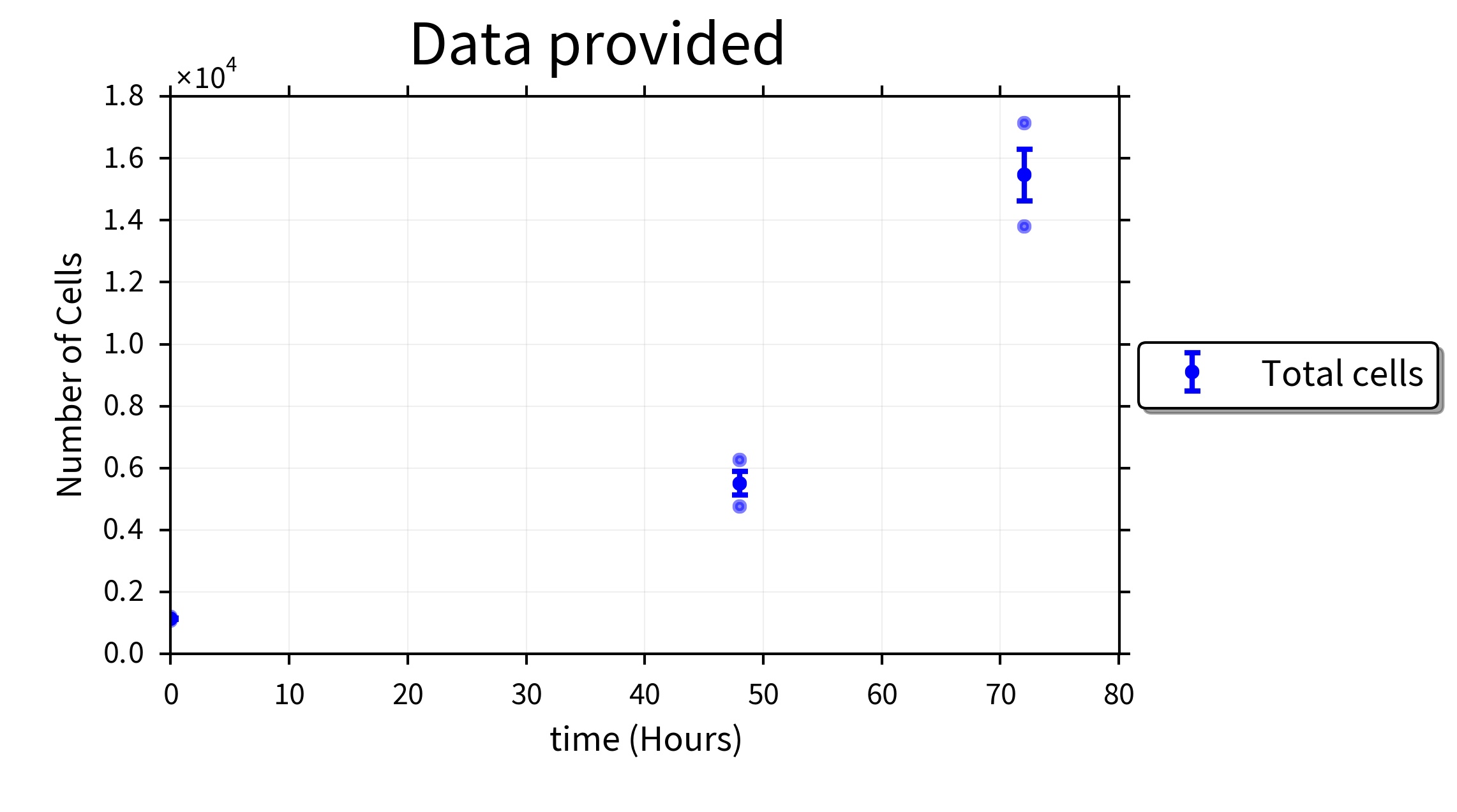

Supplement: Additional file 1: — Example of CellPD’s outputs. This folder contains two examples of the outputs generated by CellPD (using the data from Fig. 2 and Additional file 6). (ZIP 36462 kb) [file 12918_2016_337_MOESM1_ESM.zip › USC/output/data/Data.jpg]

# Data provided

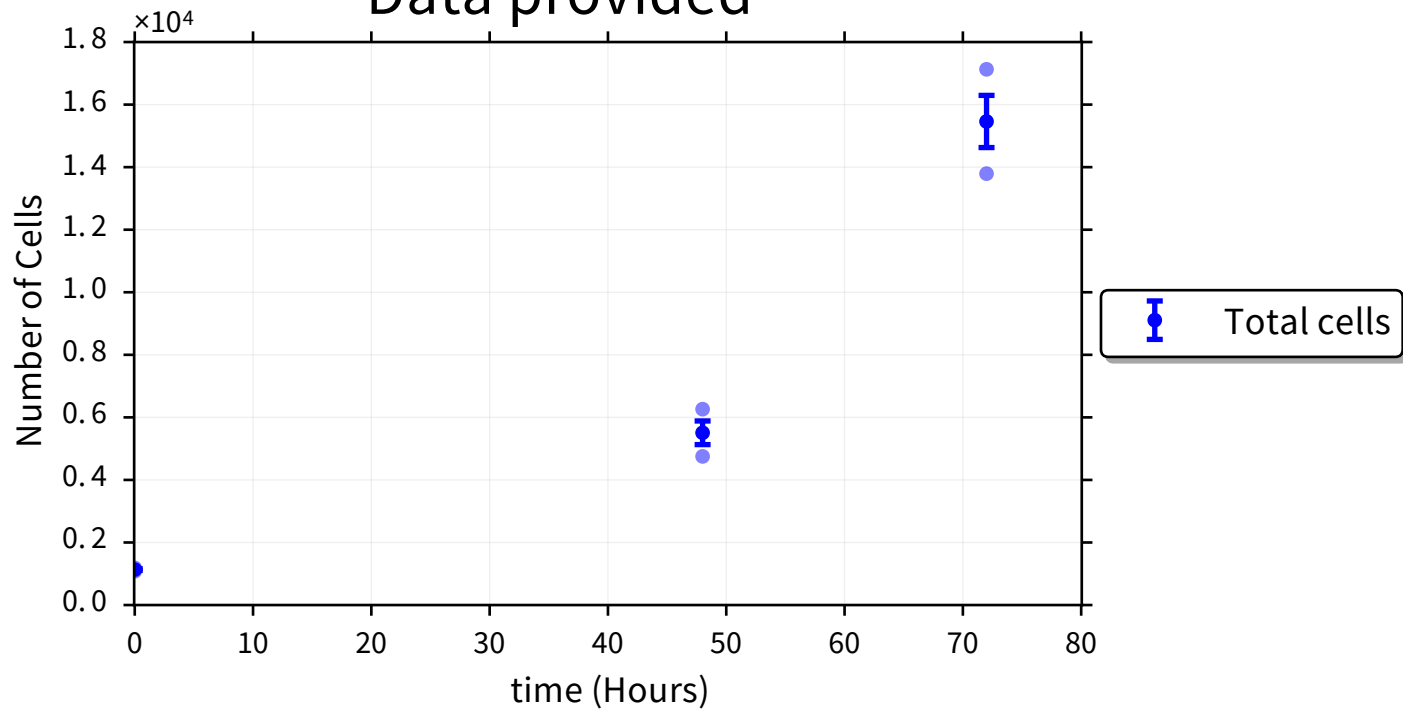

Supplement: Additional file 1: — Example of CellPD’s outputs. This folder contains two examples of the outputs generated by CellPD (using the data from Fig. 2 and Additional file 6). (ZIP 36462 kb) [file 12918_2016_337_MOESM1_ESM.zip › USC/output/data/Data.pdf]

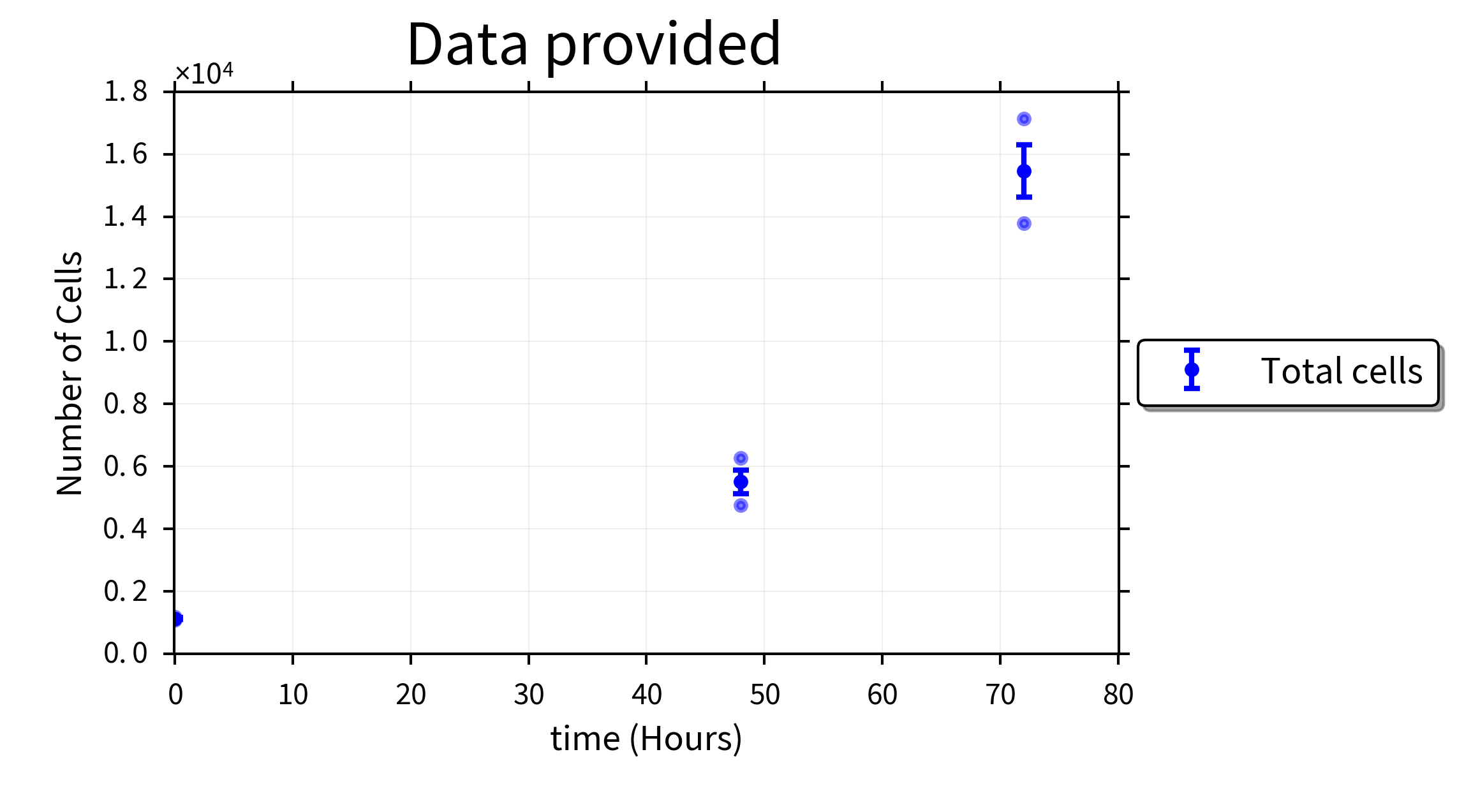

Supplement: Additional file 1: — Example of CellPD’s outputs. This folder contains two examples of the outputs generated by CellPD (using the data from Fig. 2 and Additional file 6). (ZIP 36462 kb) [file 12918_2016_337_MOESM1_ESM.zip › USC/output/data/Data.png]

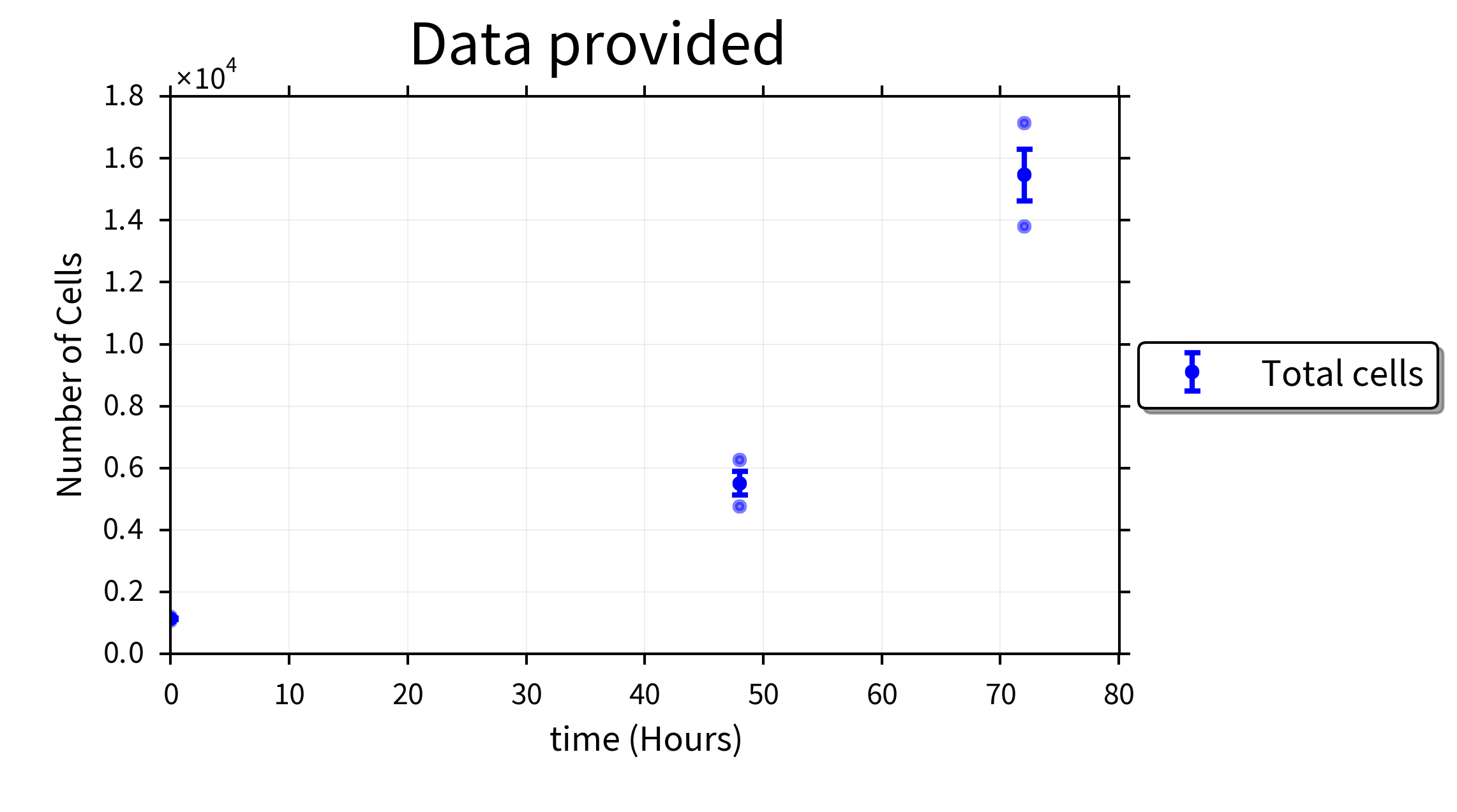

Supplement: Additional file 1: — Example of CellPD’s outputs. This folder contains two examples of the outputs generated by CellPD (using the data from Fig. 2 and Additional file 6). (ZIP 36462 kb) [file 12918_2016_337_MOESM1_ESM.zip › USC/output/data/Data.tiff]

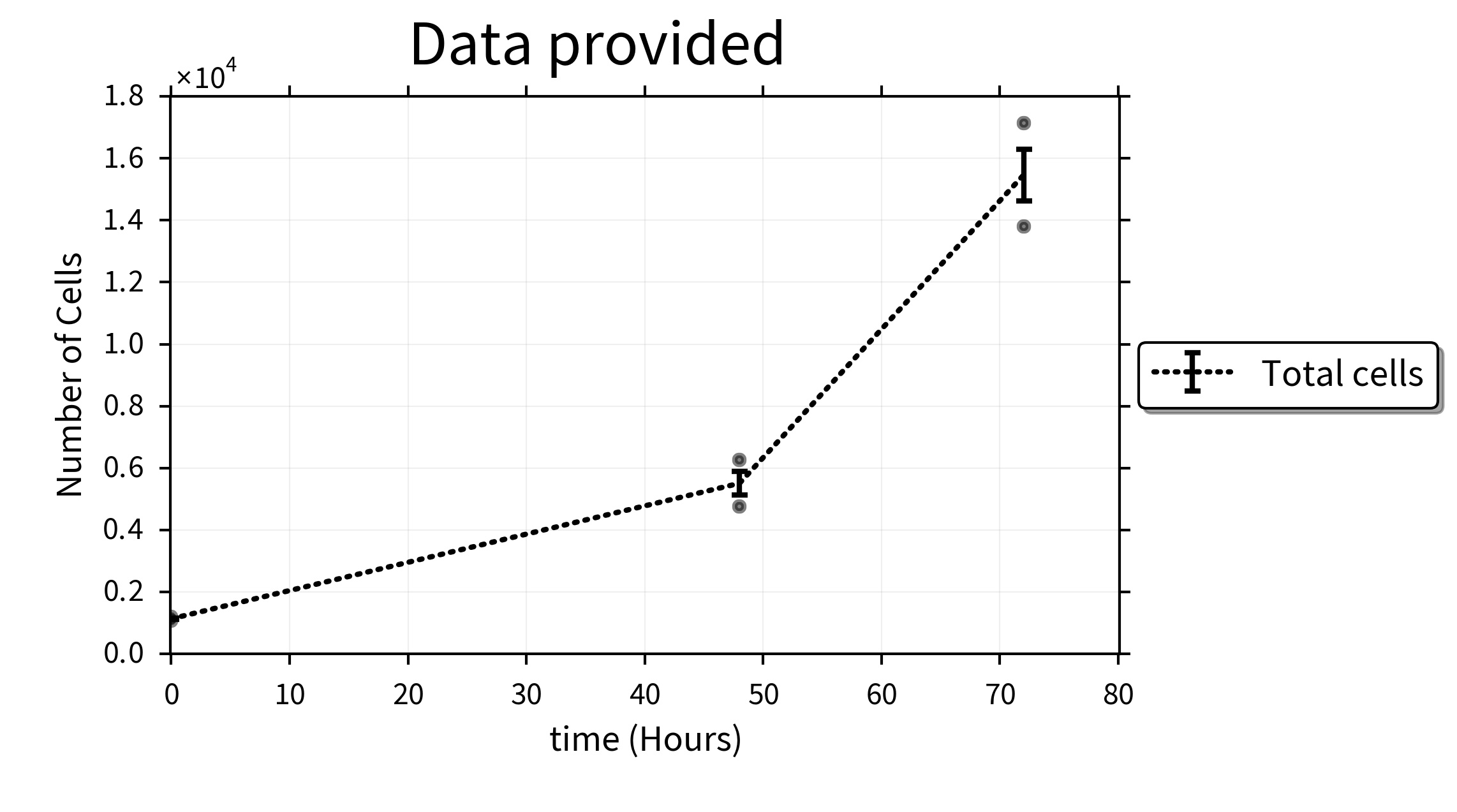

Supplement: Additional file 1: — Example of CellPD’s outputs. This folder contains two examples of the outputs generated by CellPD (using the data from Fig. 2 and Additional file 6). (ZIP 36462 kb) [file 12918_2016_337_MOESM1_ESM.zip › USC/output/data/Data_BW.jpg]

# Data provided

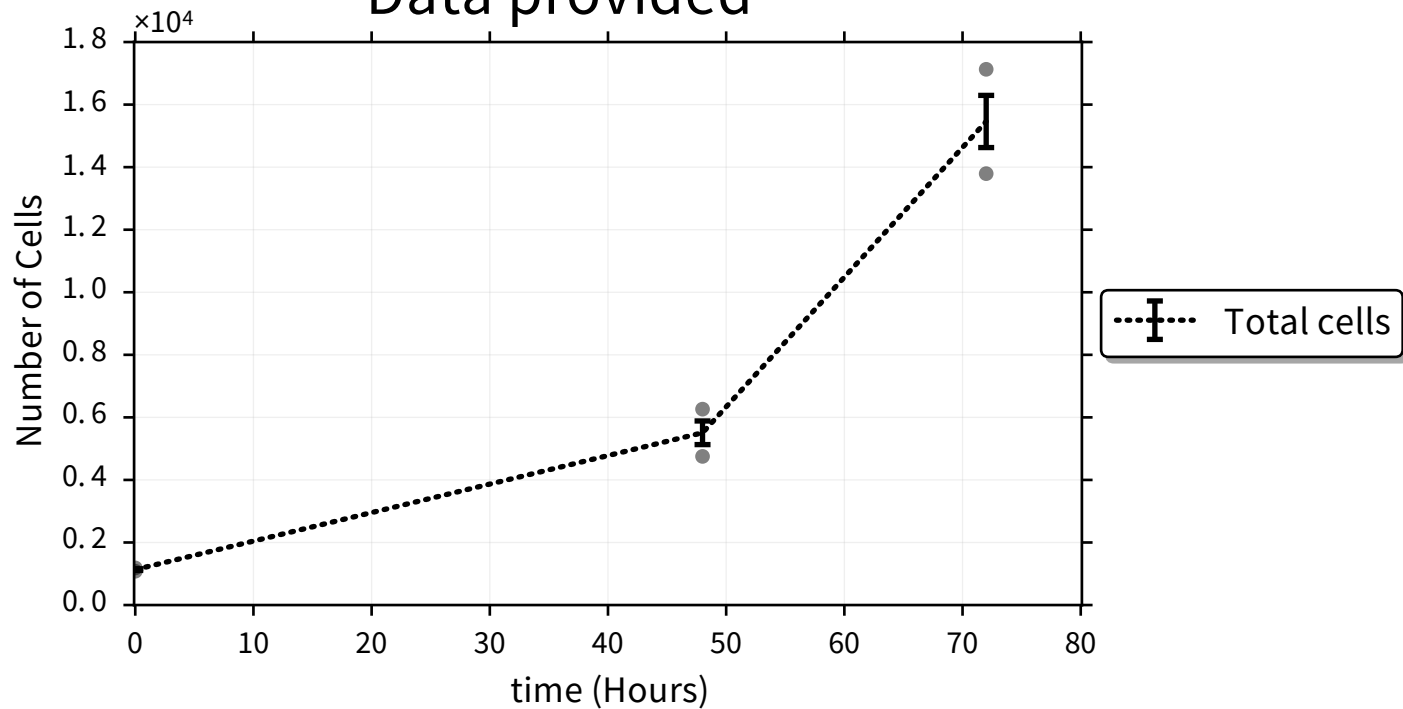

Supplement: Additional file 1: — Example of CellPD’s outputs. This folder contains two examples of the outputs generated by CellPD (using the data from Fig. 2 and Additional file 6). (ZIP 36462 kb) [file 12918_2016_337_MOESM1_ESM.zip › USC/output/data/Data_BW.pdf]

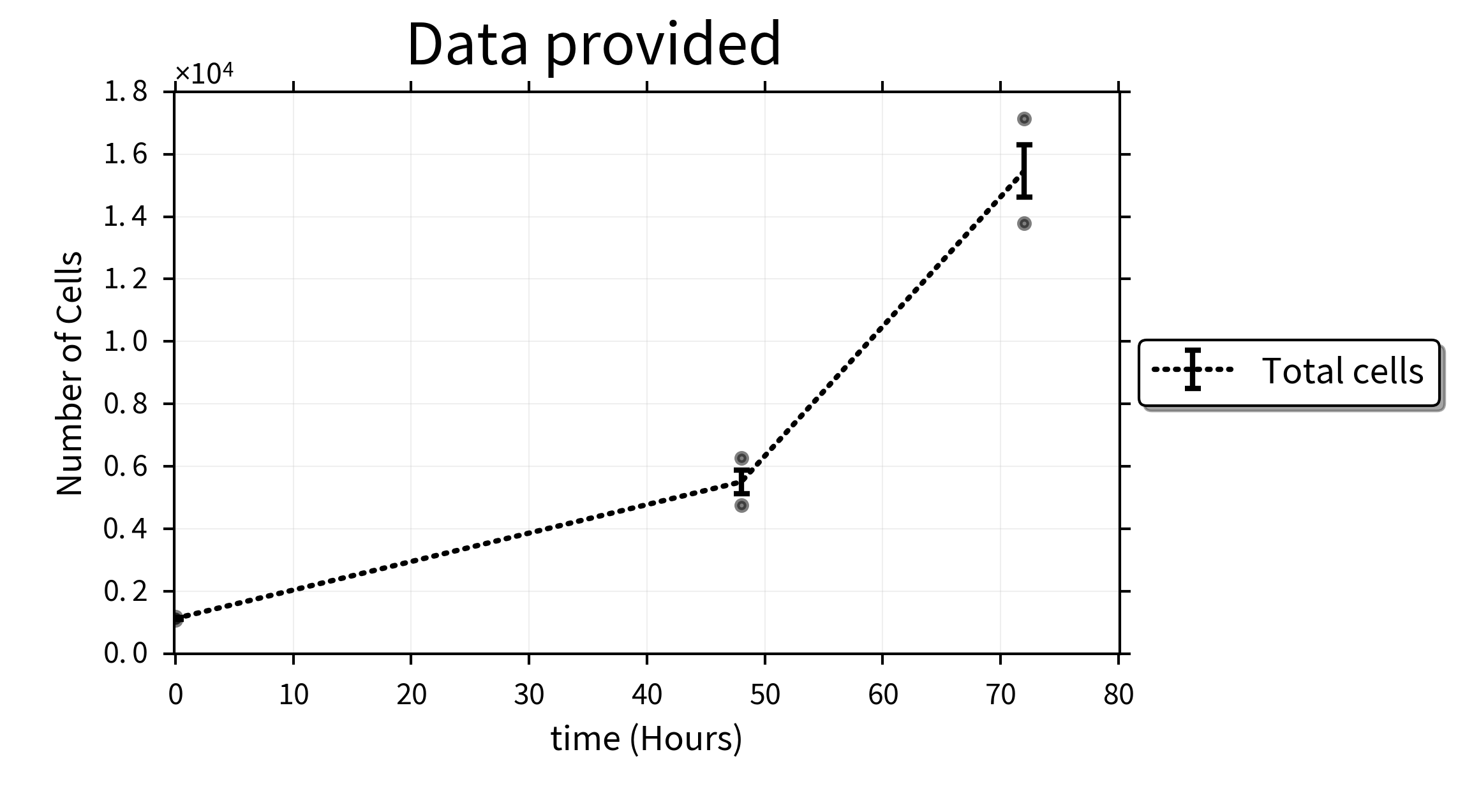

Supplement: Additional file 1: — Example of CellPD’s outputs. This folder contains two examples of the outputs generated by CellPD (using the data from Fig. 2 and Additional file 6). (ZIP 36462 kb) [file 12918_2016_337_MOESM1_ESM.zip › USC/output/data/Data_BW.png]

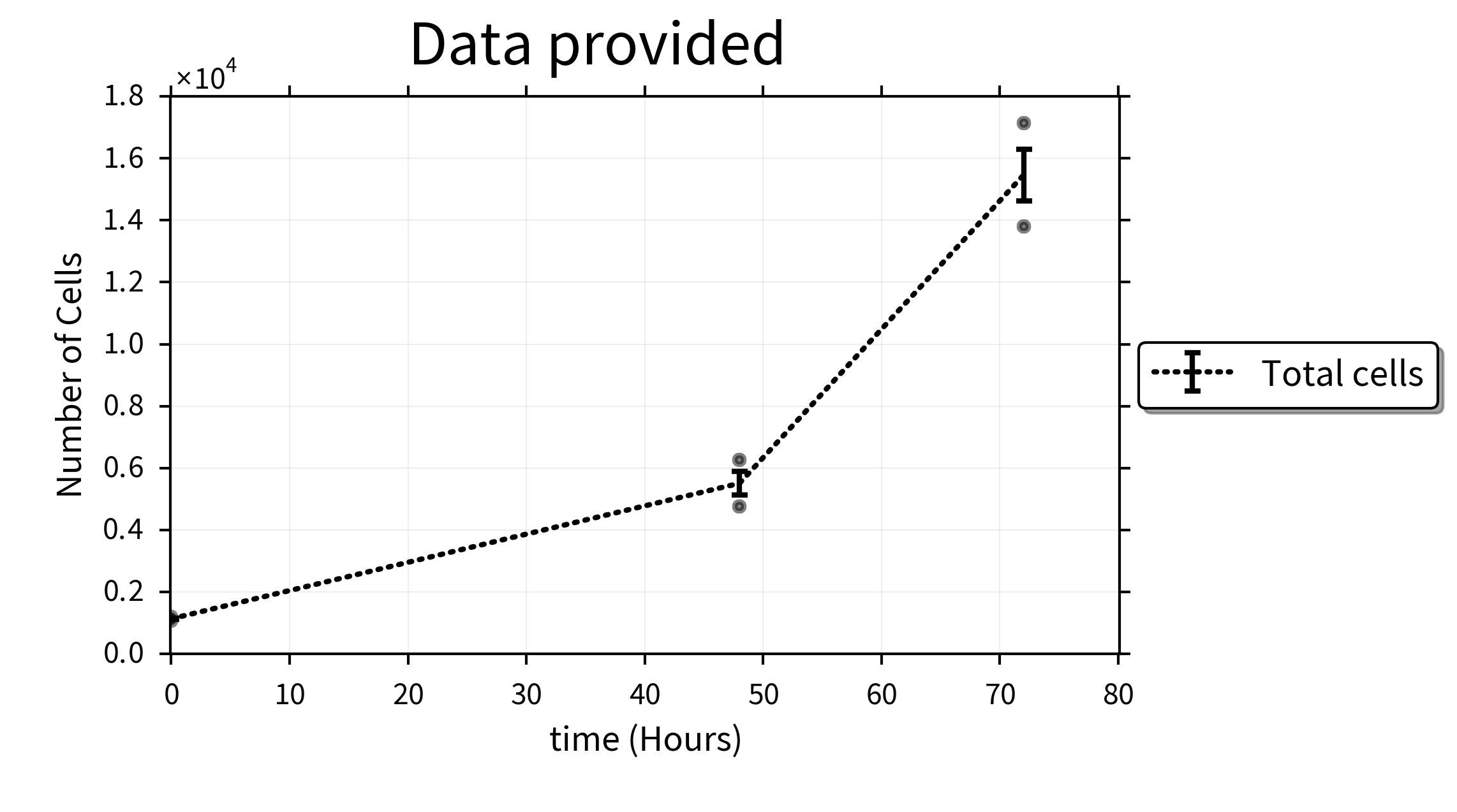

Supplement: Additional file 1: — Example of CellPD’s outputs. This folder contains two examples of the outputs generated by CellPD (using the data from Fig. 2 and Additional file 6). (ZIP 36462 kb) [file 12918_2016_337_MOESM1_ESM.zip › USC/output/data/Data_BW.tiff]

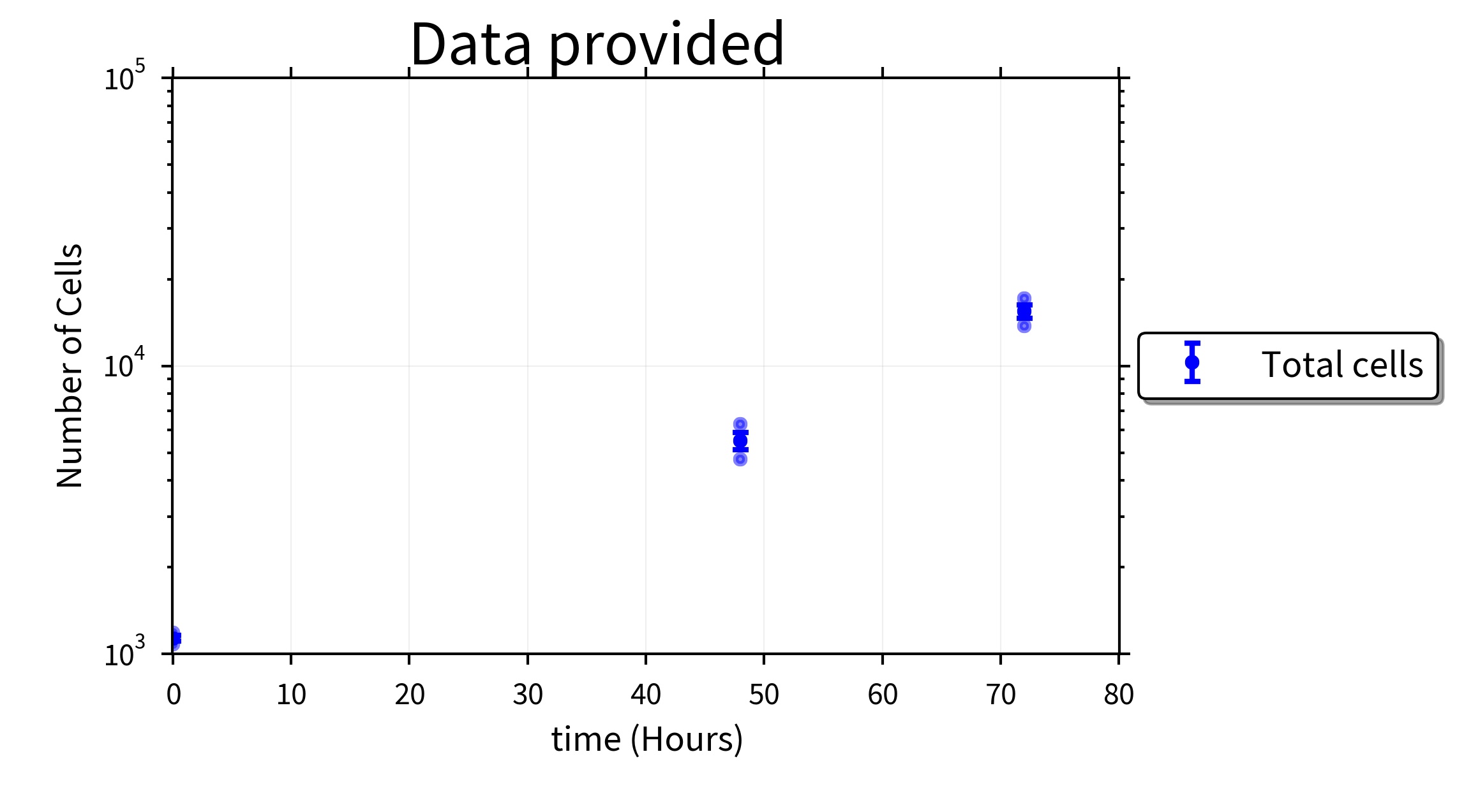

Supplement: Additional file 1: — Example of CellPD’s outputs. This folder contains two examples of the outputs generated by CellPD (using the data from Fig. 2 and Additional file 6). (ZIP 36462 kb) [file 12918_2016_337_MOESM1_ESM.zip › USC/output/data/Data_loglinear.jpg]

# Data provided

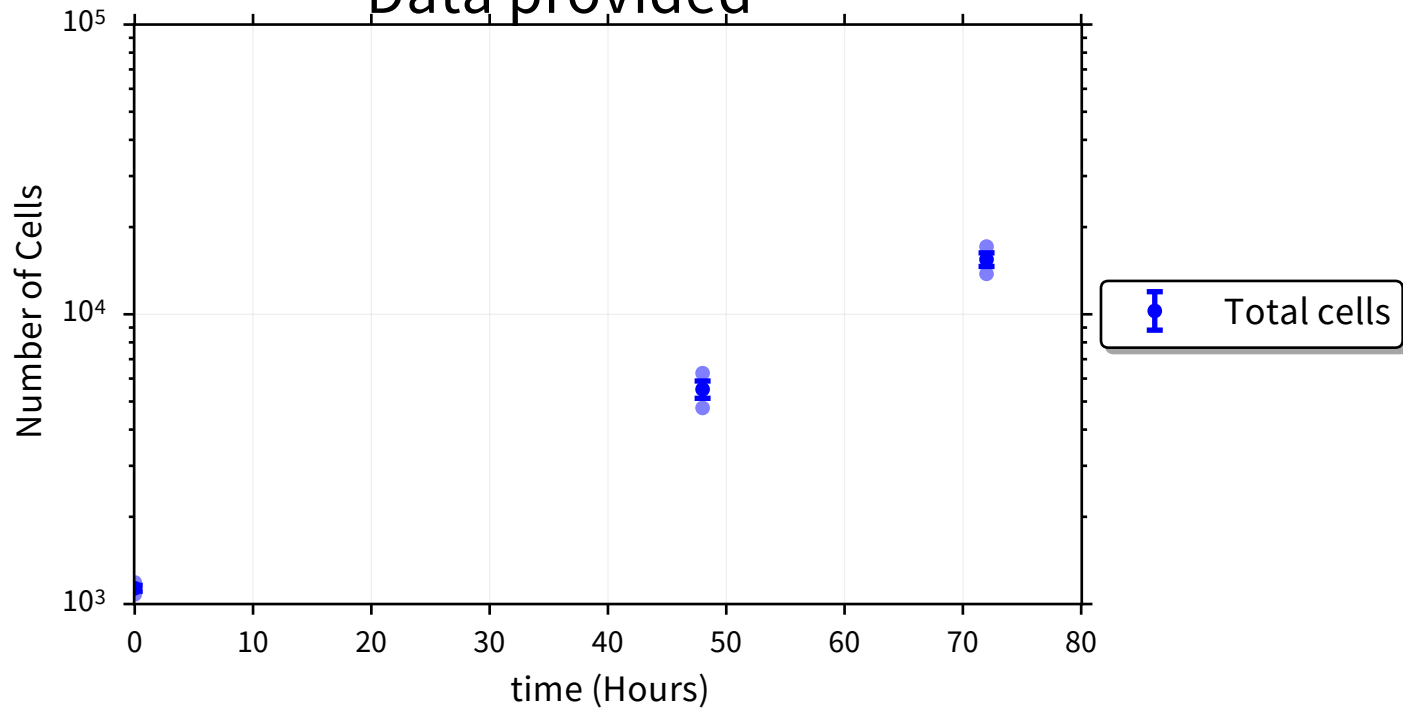

Supplement: Additional file 1: — Example of CellPD’s outputs. This folder contains two examples of the outputs generated by CellPD (using the data from Fig. 2 and Additional file 6). (ZIP 36462 kb) [file 12918_2016_337_MOESM1_ESM.zip › USC/output/data/Data_loglinear.pdf]

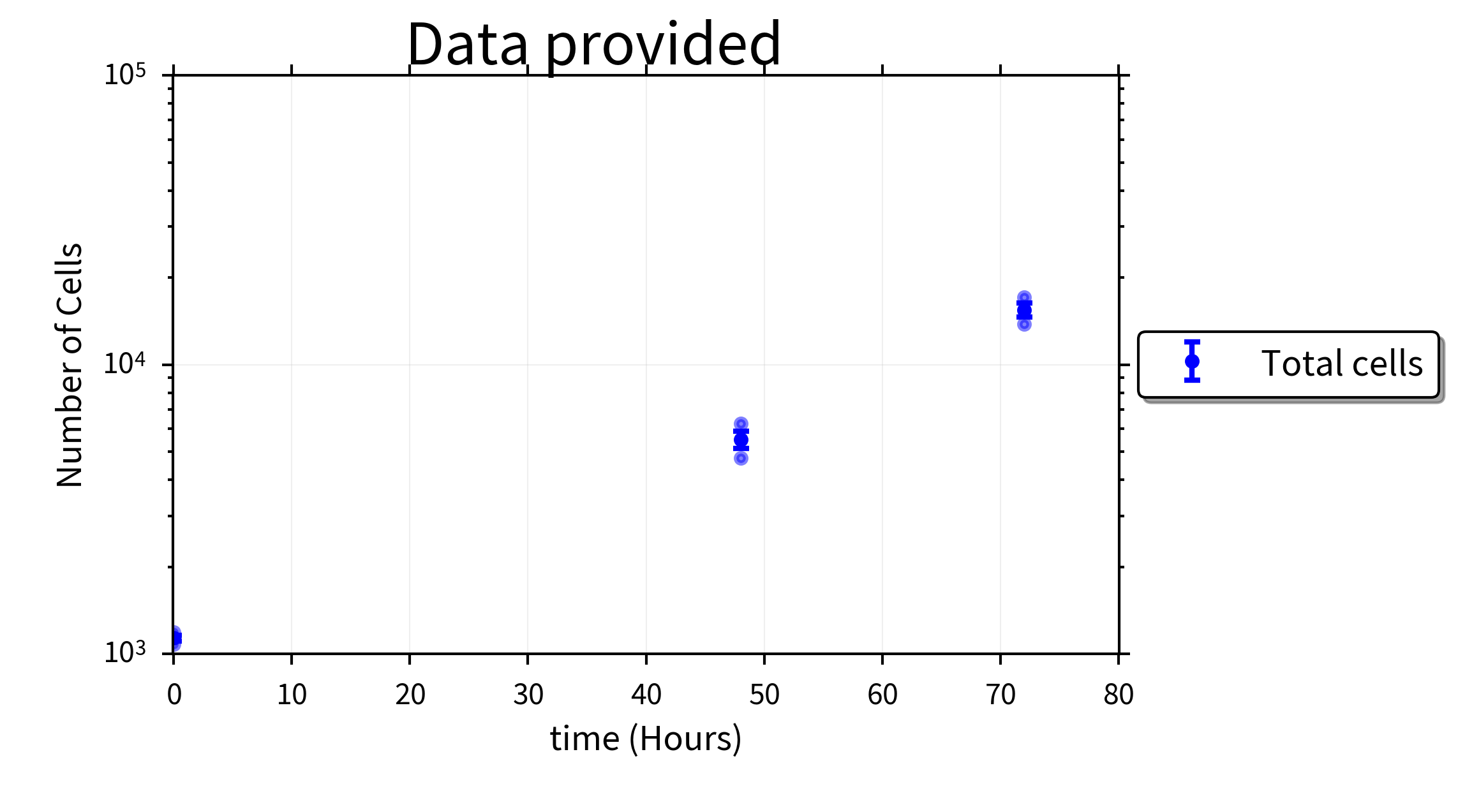

Supplement: Additional file 1: — Example of CellPD’s outputs. This folder contains two examples of the outputs generated by CellPD (using the data from Fig. 2 and Additional file 6). (ZIP 36462 kb) [file 12918_2016_337_MOESM1_ESM.zip › USC/output/data/Data_loglinear.png]

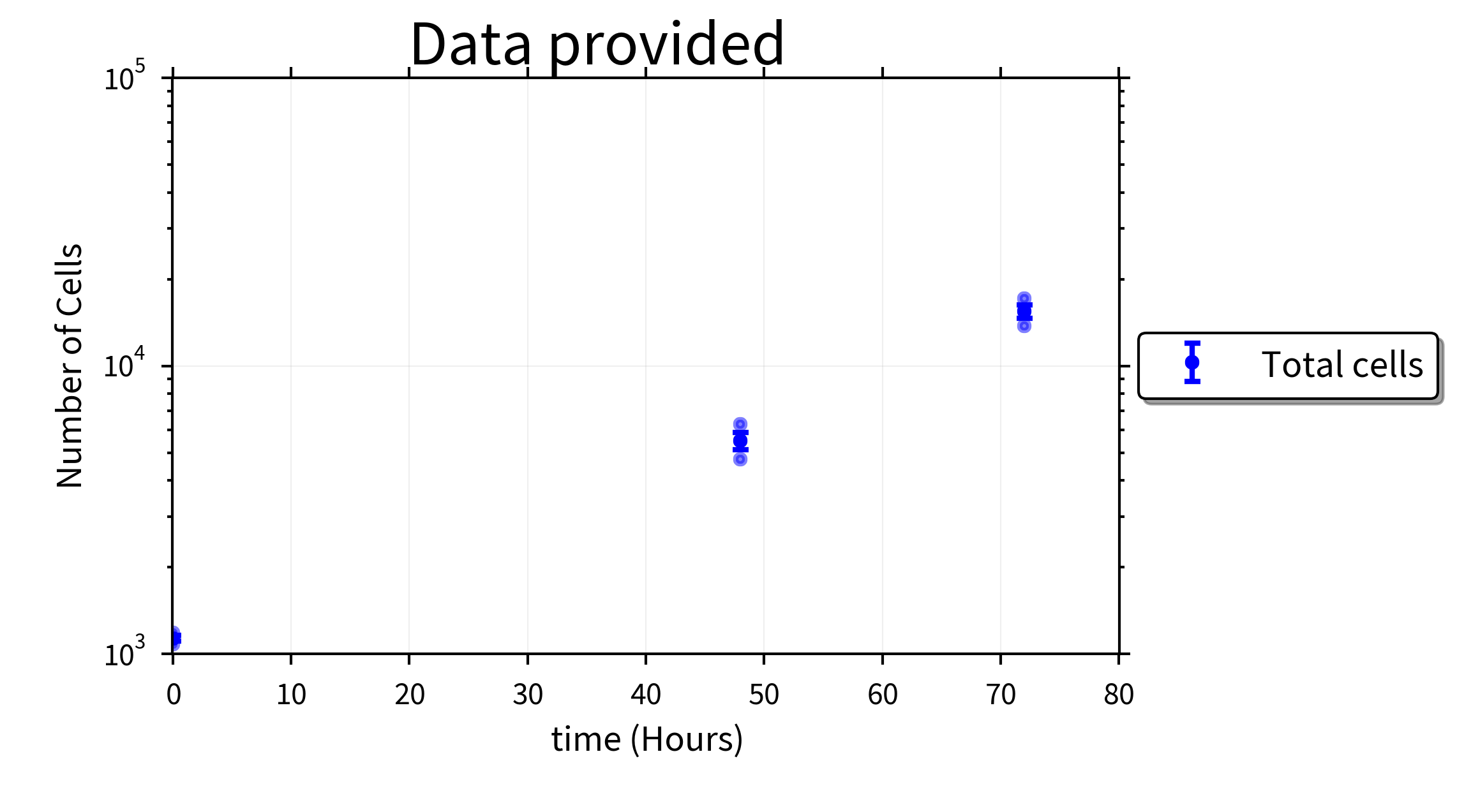

Supplement: Additional file 1: — Example of CellPD’s outputs. This folder contains two examples of the outputs generated by CellPD (using the data from Fig. 2 and Additional file 6). (ZIP 36462 kb) [file 12918_2016_337_MOESM1_ESM.zip › USC/output/data/Data_loglinear.tiff]

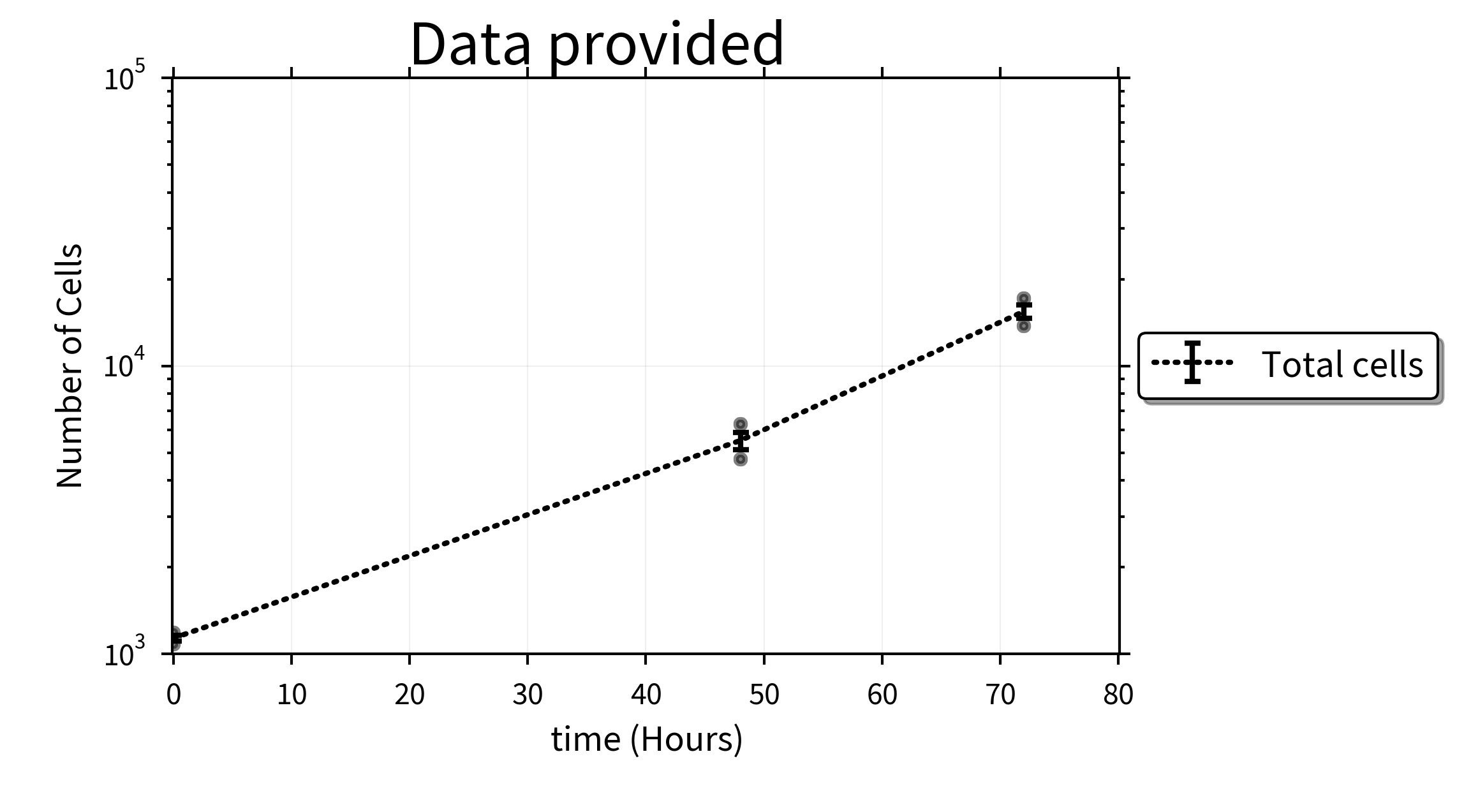

Supplement: Additional file 1: — Example of CellPD’s outputs. This folder contains two examples of the outputs generated by CellPD (using the data from Fig. 2 and Additional file 6). (ZIP 36462 kb) [file 12918_2016_337_MOESM1_ESM.zip › USC/output/data/Data_loglinear_BW.jpg]

# Data provided

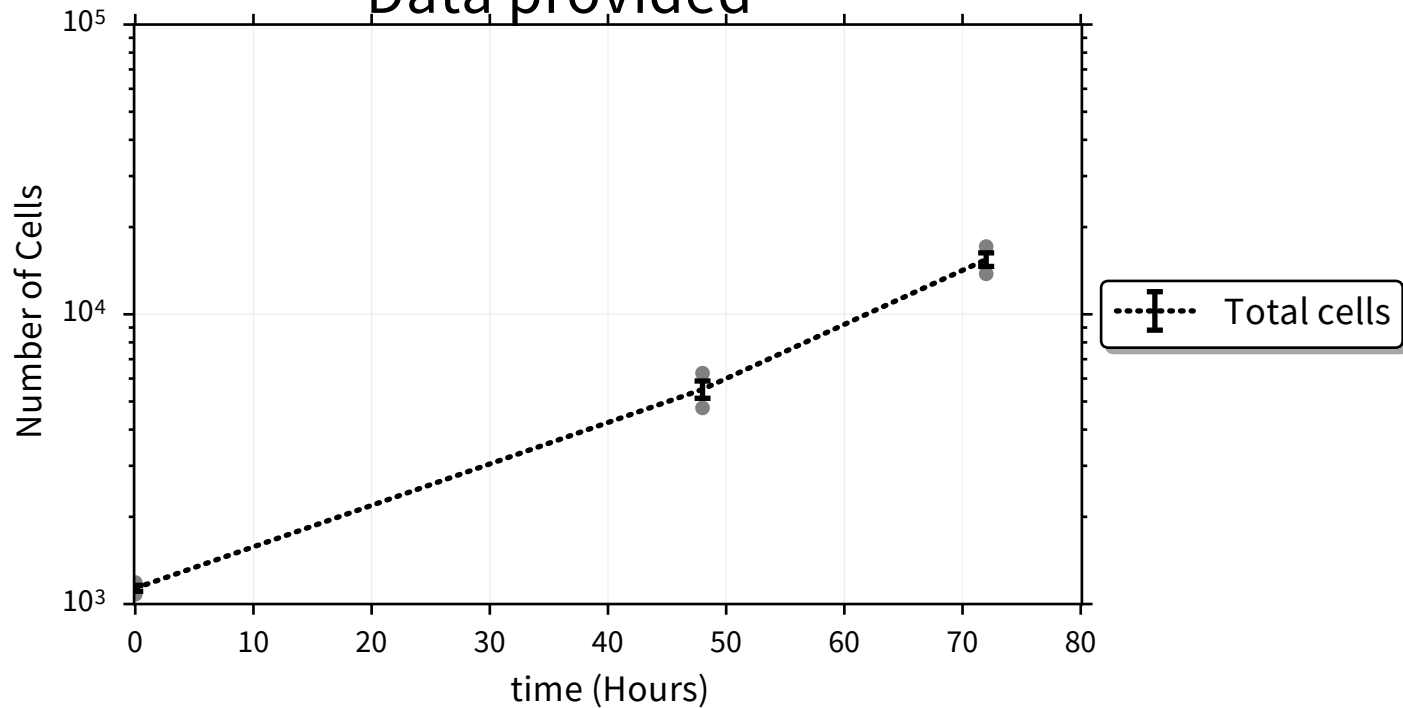

Supplement: Additional file 1: — Example of CellPD’s outputs. This folder contains two examples of the outputs generated by CellPD (using the data from Fig. 2 and Additional file 6). (ZIP 36462 kb) [file 12918_2016_337_MOESM1_ESM.zip › USC/output/data/Data_loglinear_BW.pdf]

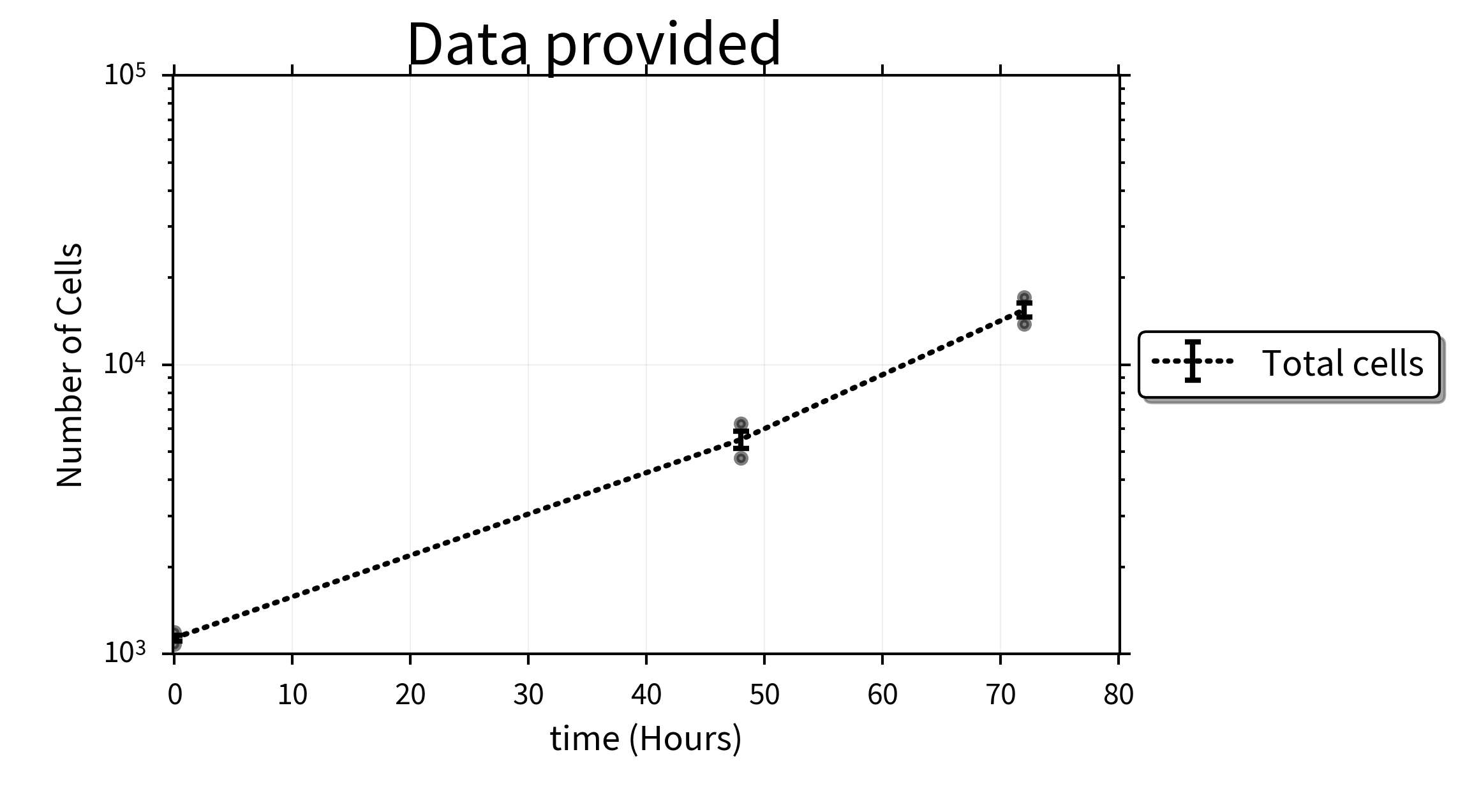

Supplement: Additional file 1: — Example of CellPD’s outputs. This folder contains two examples of the outputs generated by CellPD (using the data from Fig. 2 and Additional file 6). (ZIP 36462 kb) [file 12918_2016_337_MOESM1_ESM.zip › USC/output/data/Data_loglinear_BW.png]

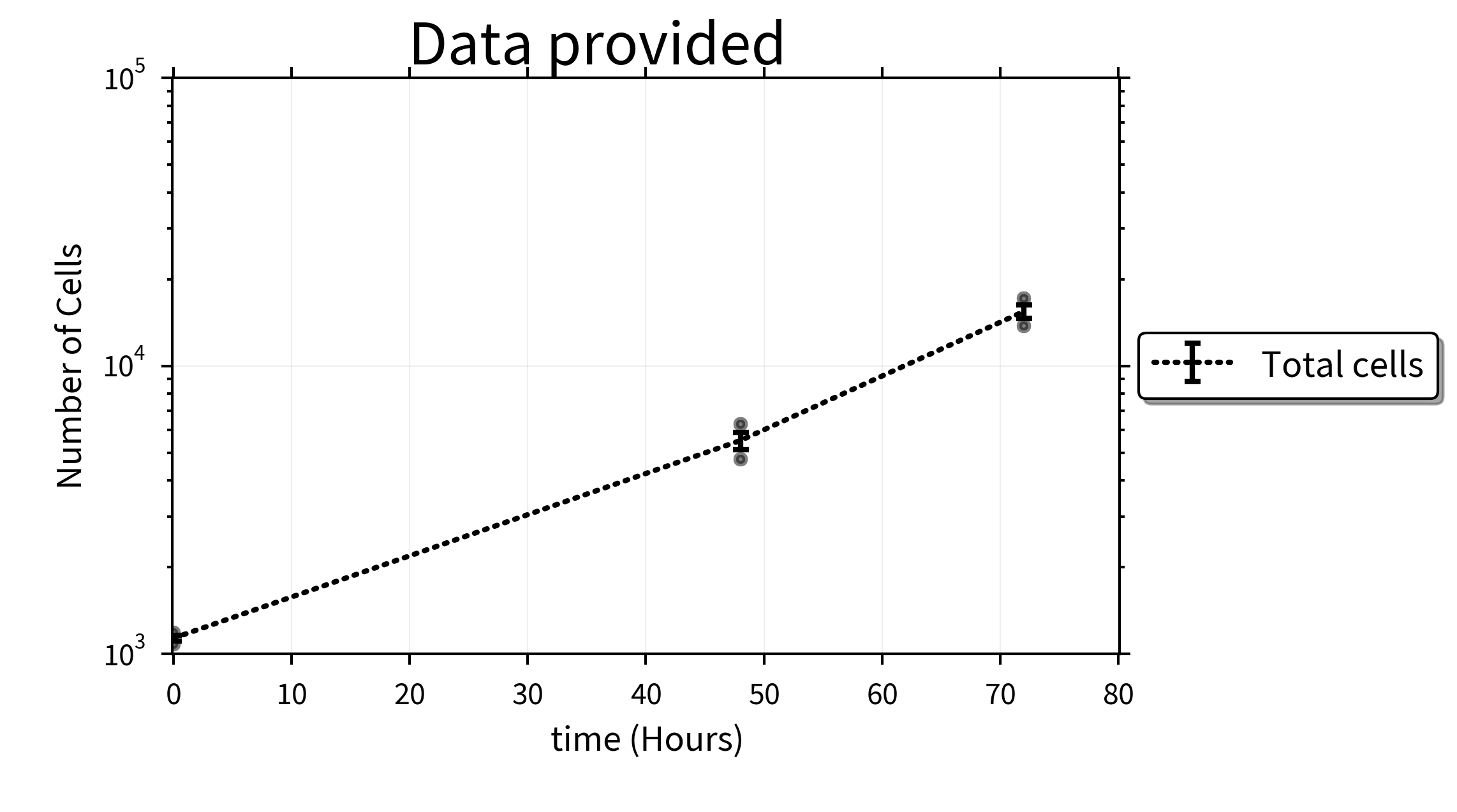

Supplement: Additional file 1: — Example of CellPD’s outputs. This folder contains two examples of the outputs generated by CellPD (using the data from Fig. 2 and Additional file 6). (ZIP 36462 kb) [file 12918_2016_337_MOESM1_ESM.zip › USC/output/data/Data_loglinear_BW.tiff]

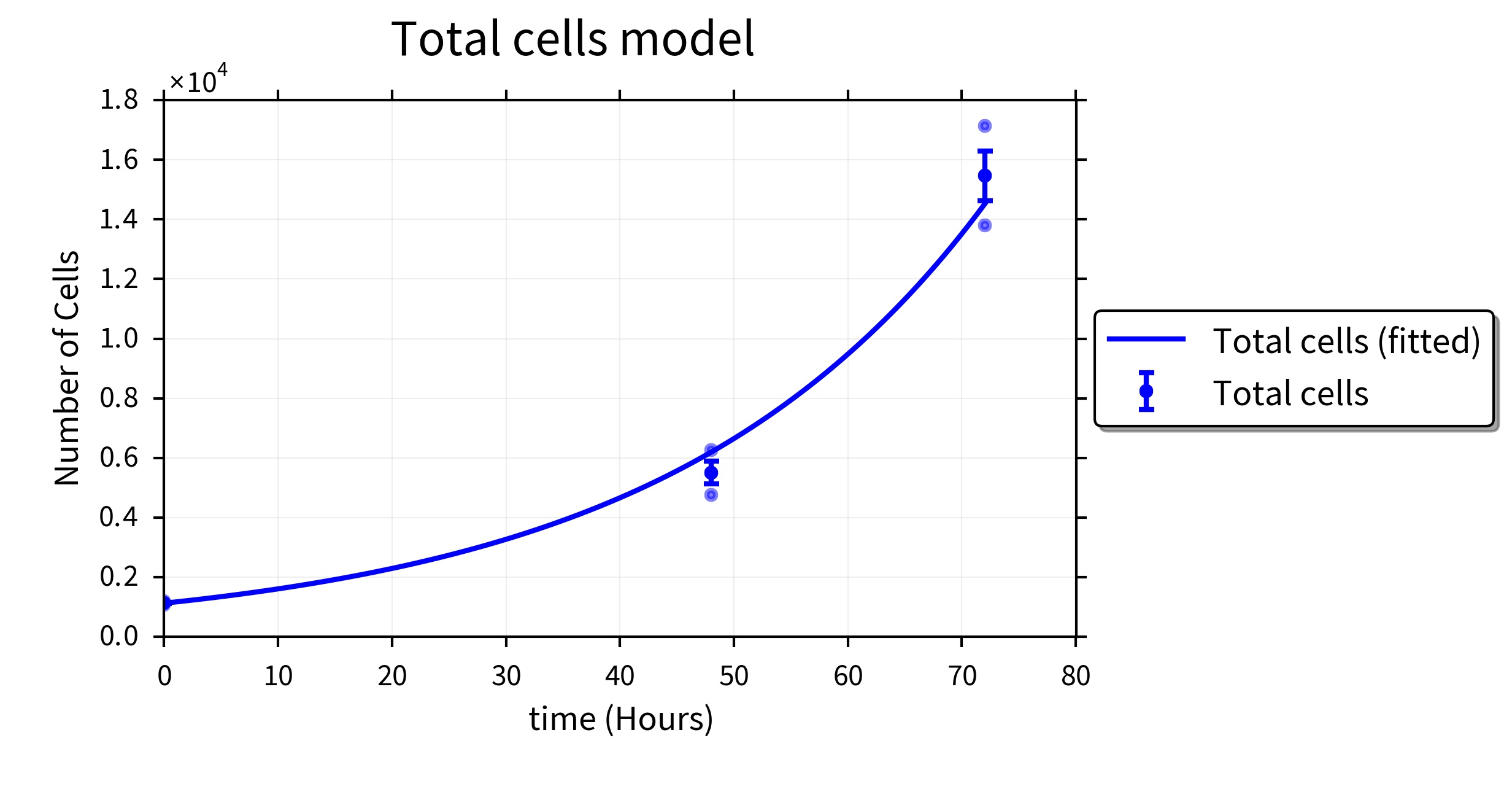

Supplement: Additional file 1: — Example of CellPD’s outputs. This folder contains two examples of the outputs generated by CellPD (using the data from Fig. 2 and Additional file 6). (ZIP 36462 kb) [file 12918_2016_337_MOESM1_ESM.zip › USC/output/total/total.jpg]

# Total cells model

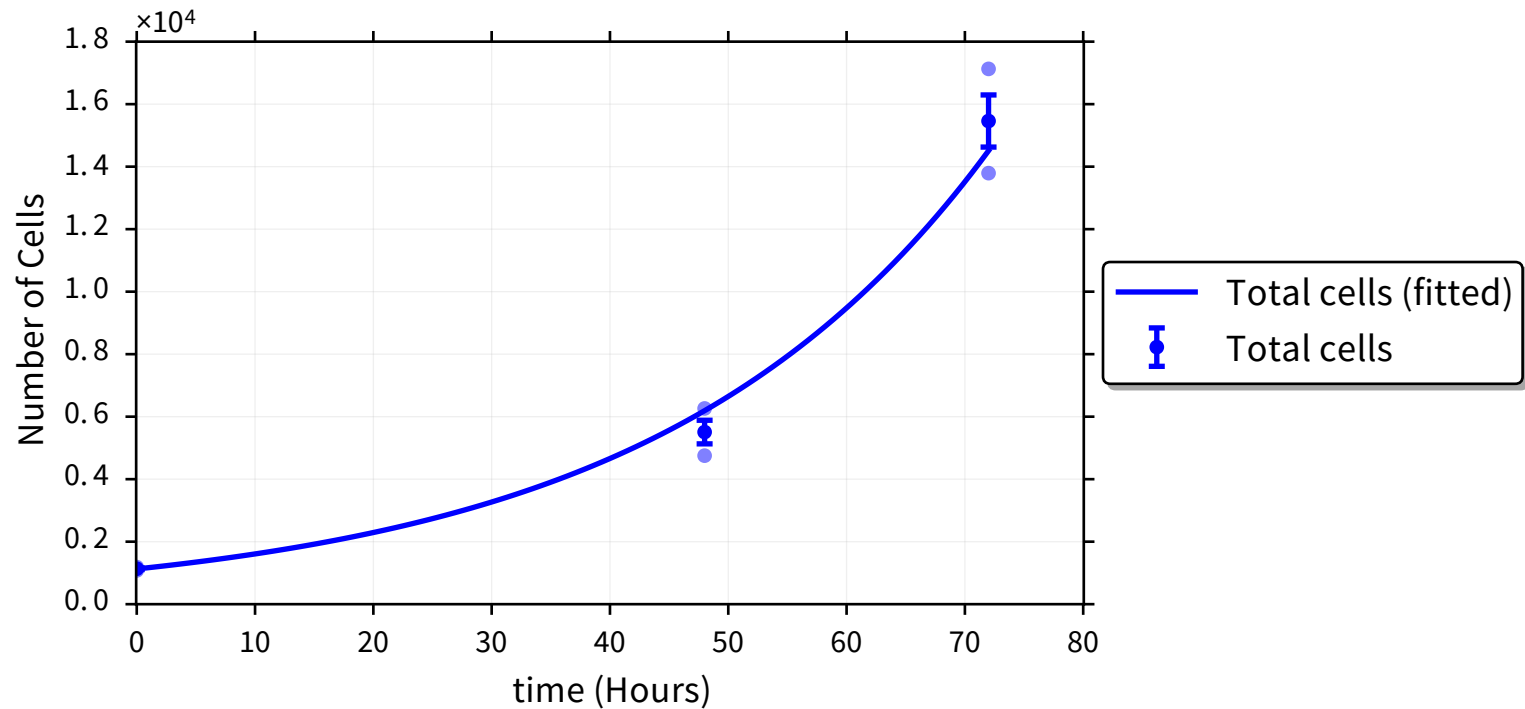

Supplement: Additional file 1: — Example of CellPD’s outputs. This folder contains two examples of the outputs generated by CellPD (using the data from Fig. 2 and Additional file 6). (ZIP 36462 kb) [file 12918_2016_337_MOESM1_ESM.zip › USC/output/total/total.pdf]

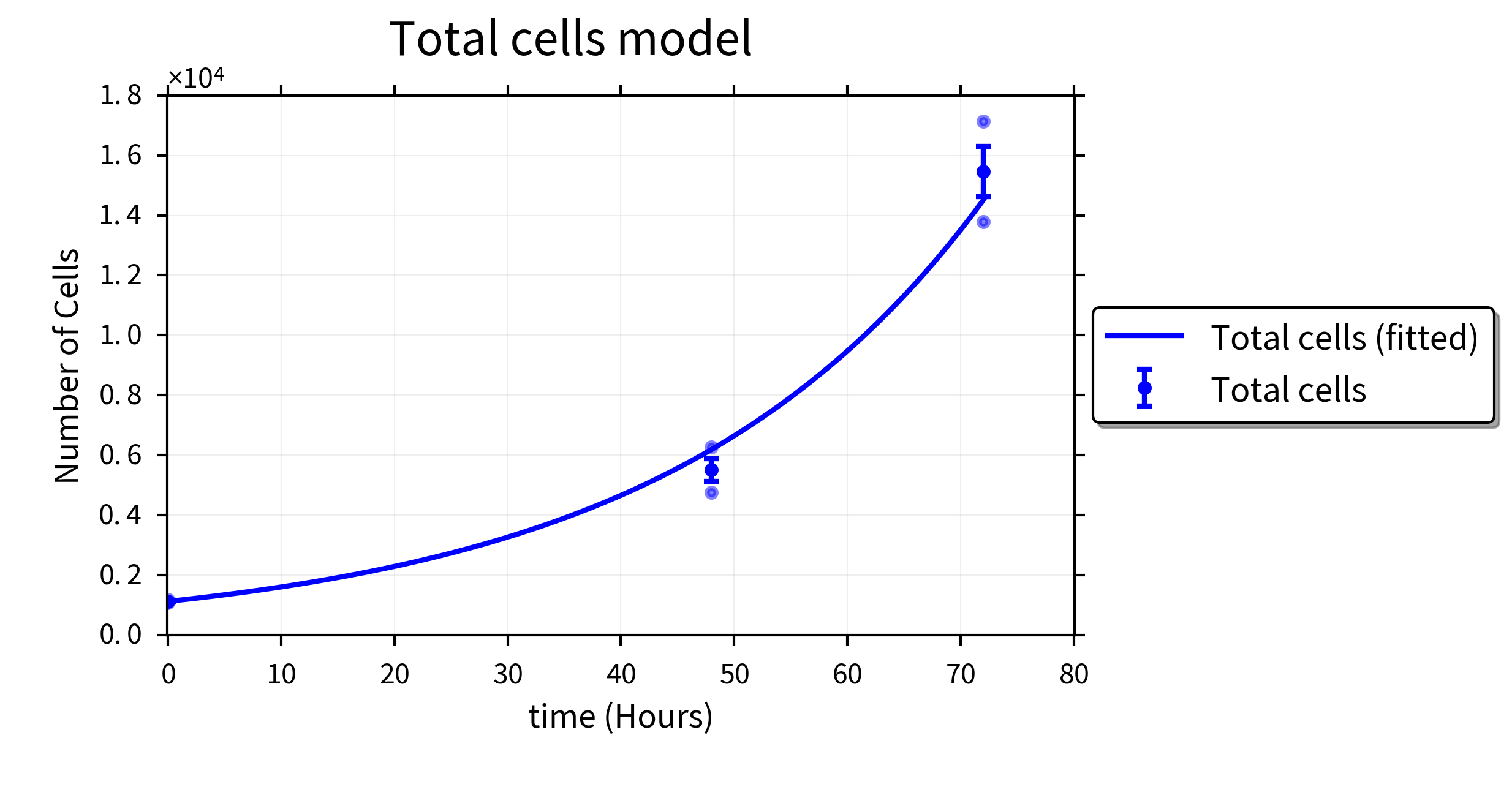

Supplement: Additional file 1: — Example of CellPD’s outputs. This folder contains two examples of the outputs generated by CellPD (using the data from Fig. 2 and Additional file 6). (ZIP 36462 kb) [file 12918_2016_337_MOESM1_ESM.zip › USC/output/total/total.png]

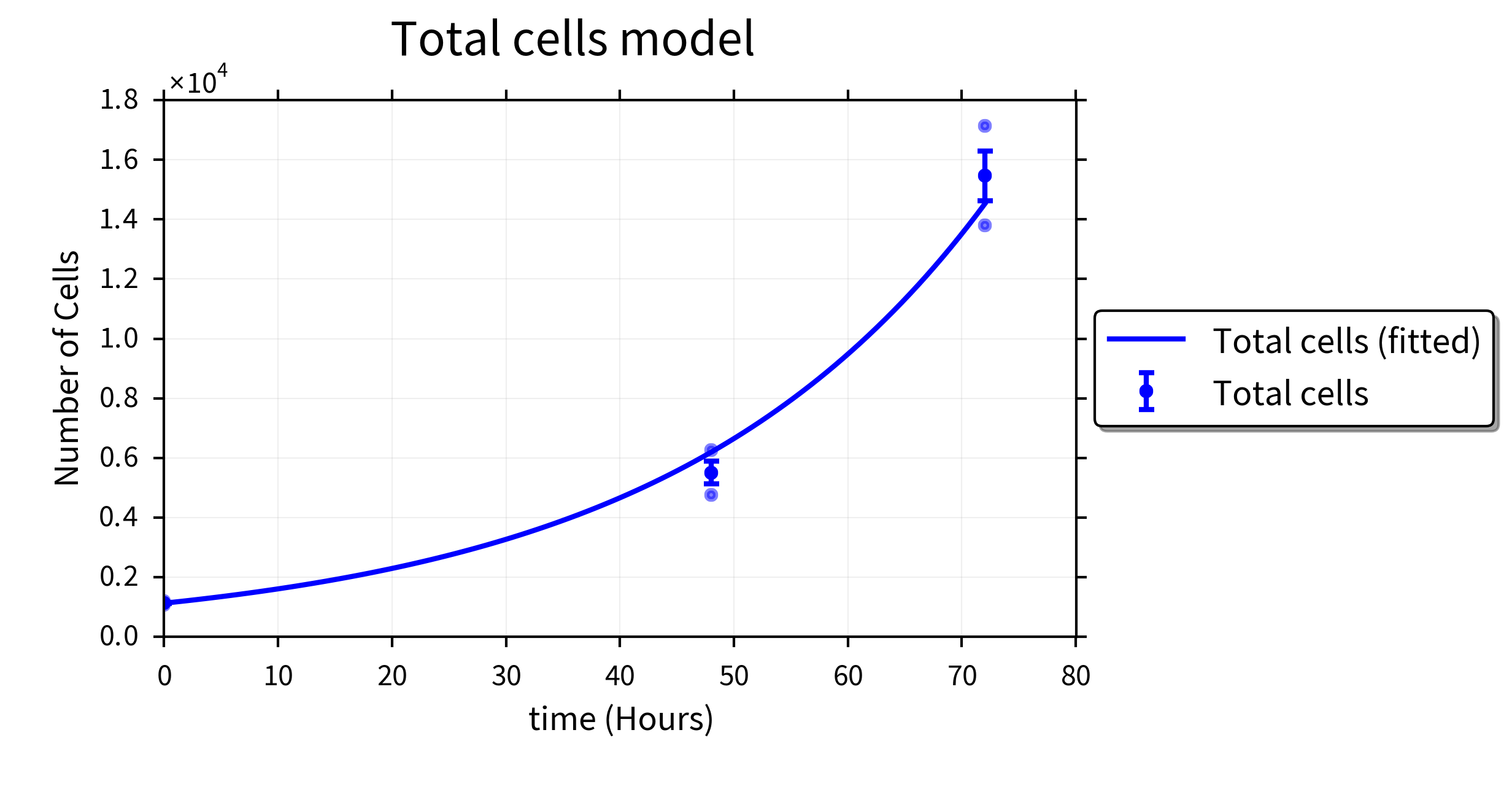

Supplement: Additional file 1: — Example of CellPD’s outputs. This folder contains two examples of the outputs generated by CellPD (using the data from Fig. 2 and Additional file 6). (ZIP 36462 kb) [file 12918_2016_337_MOESM1_ESM.zip › USC/output/total/total.tiff]

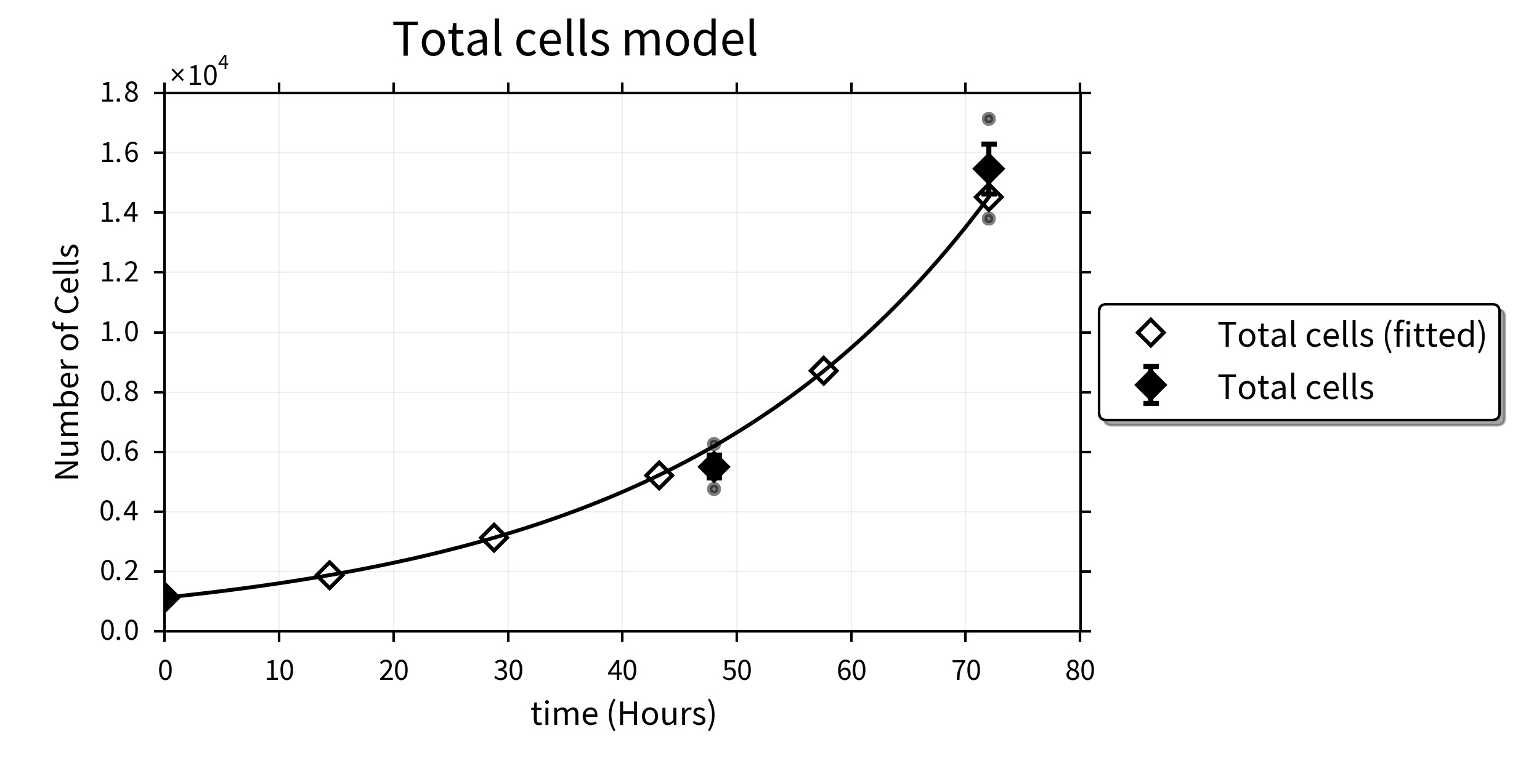

Supplement: Additional file 1: — Example of CellPD’s outputs. This folder contains two examples of the outputs generated by CellPD (using the data from Fig. 2 and Additional file 6). (ZIP 36462 kb) [file 12918_2016_337_MOESM1_ESM.zip › USC/output/total/total_BW.jpg]

# Total cells model

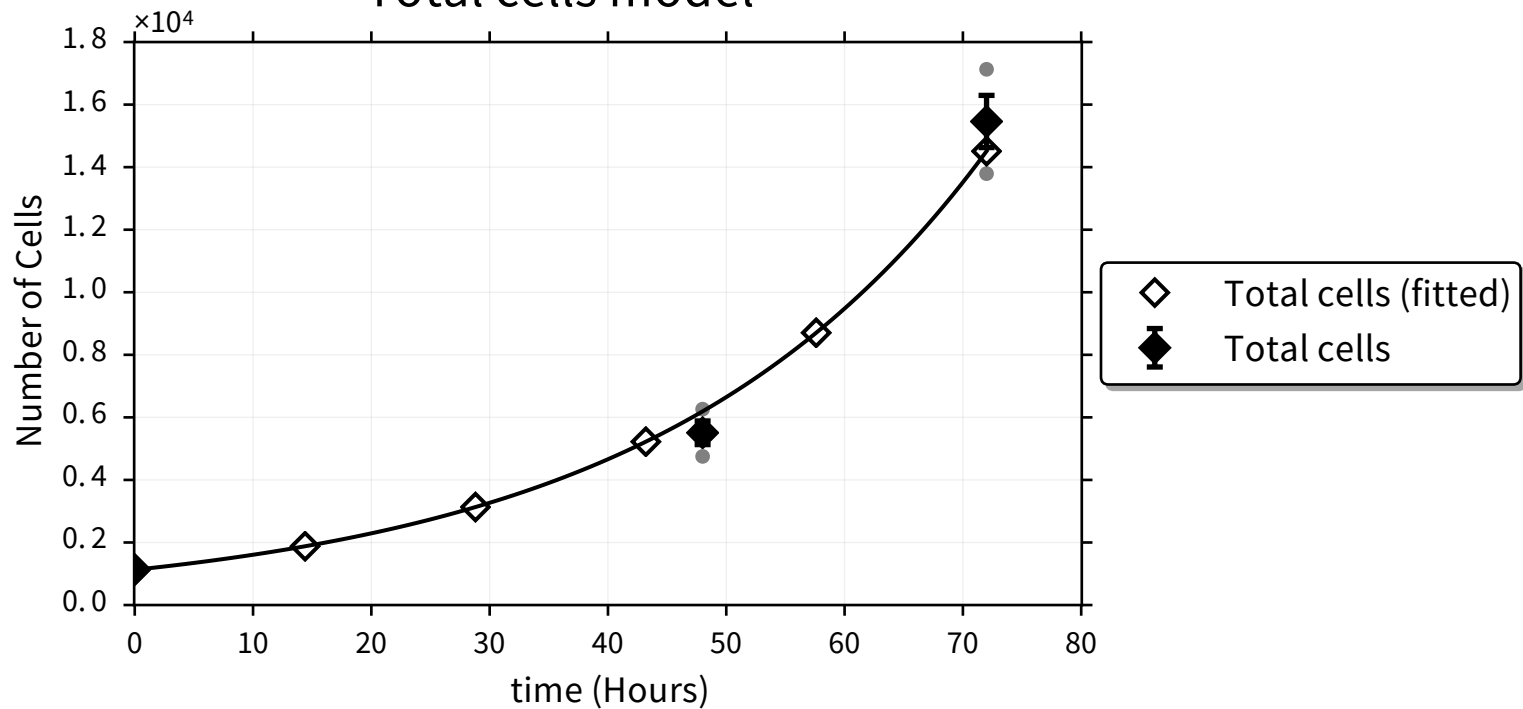

Supplement: Additional file 1: — Example of CellPD’s outputs. This folder contains two examples of the outputs generated by CellPD (using the data from Fig. 2 and Additional file 6). (ZIP 36462 kb) [file 12918_2016_337_MOESM1_ESM.zip › USC/output/total/total_BW.pdf]

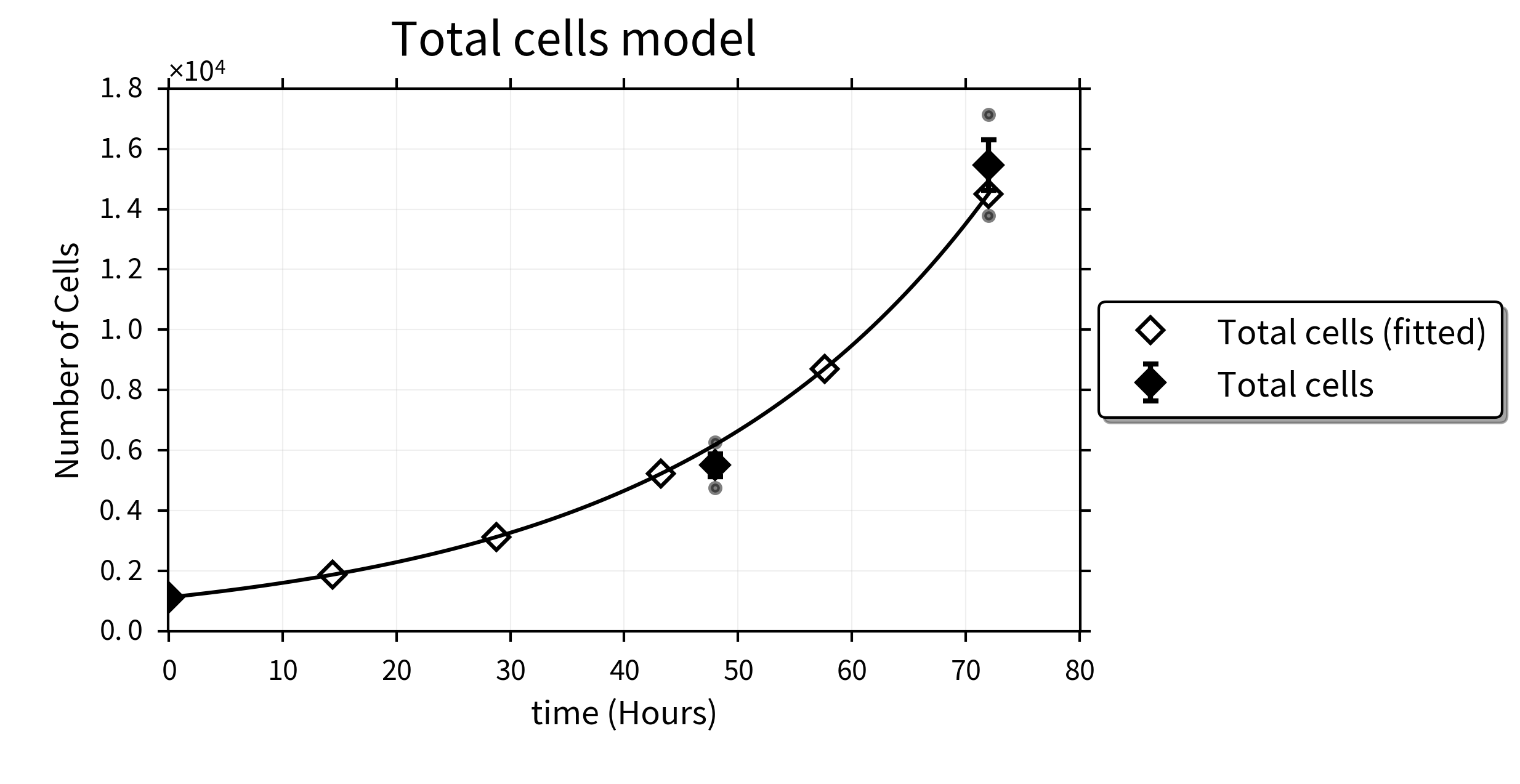

Supplement: Additional file 1: — Example of CellPD’s outputs. This folder contains two examples of the outputs generated by CellPD (using the data from Fig. 2 and Additional file 6). (ZIP 36462 kb) [file 12918_2016_337_MOESM1_ESM.zip › USC/output/total/total_BW.png]

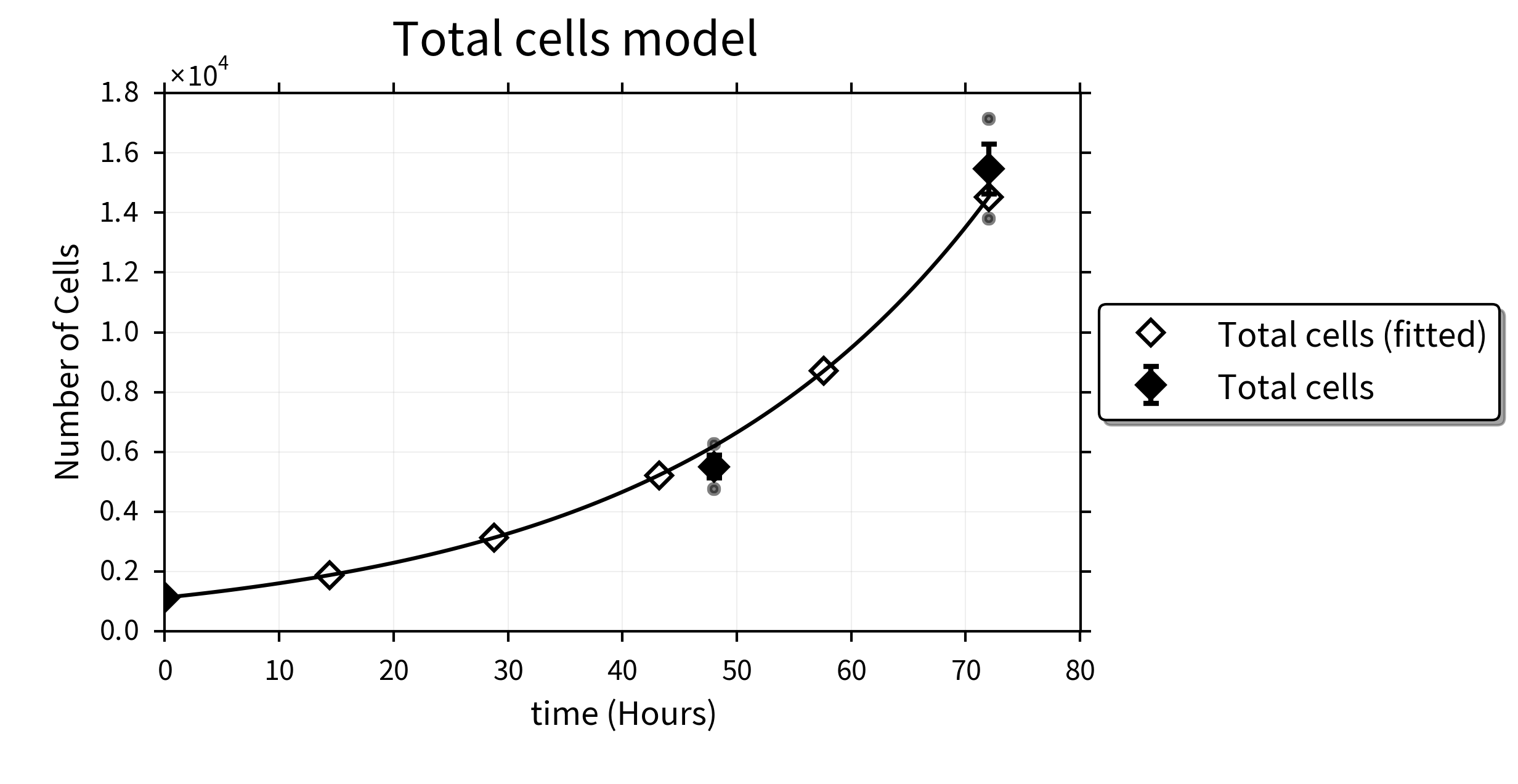

Supplement: Additional file 1: — Example of CellPD’s outputs. This folder contains two examples of the outputs generated by CellPD (using the data from Fig. 2 and Additional file 6). (ZIP 36462 kb) [file 12918_2016_337_MOESM1_ESM.zip › USC/output/total/total_BW.tiff]

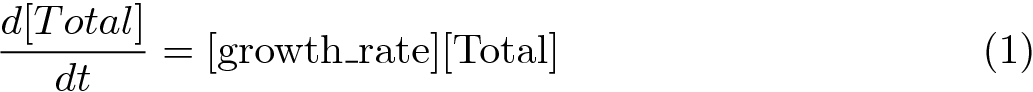

Supplement: Additional file 1: — Example of CellPD’s outputs. This folder contains two examples of the outputs generated by CellPD (using the data from Fig. 2 and Additional file 6). (ZIP 36462 kb) [file 12918_2016_337_MOESM1_ESM.zip › USC/output/total/total_eqn.png]

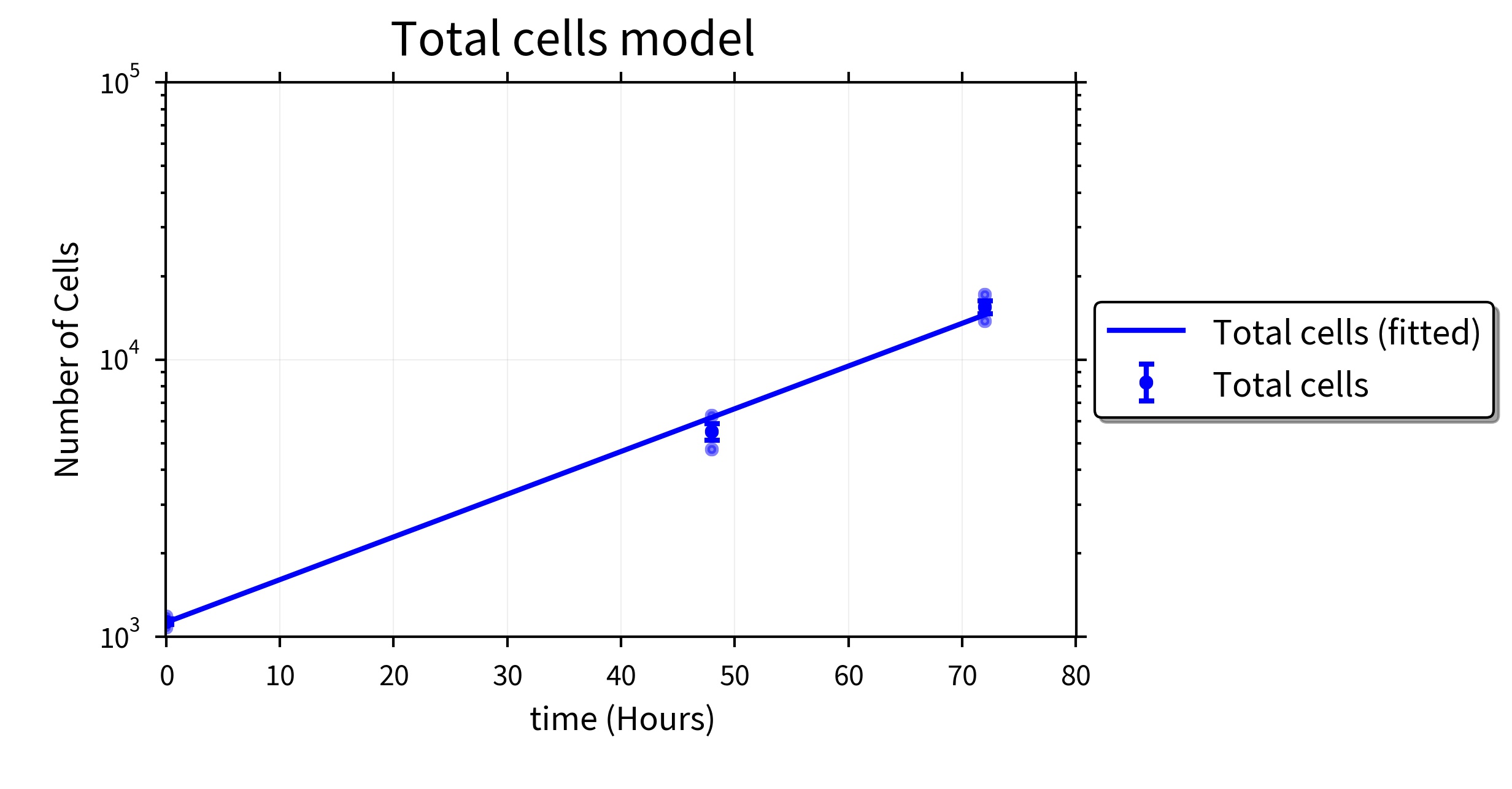

Supplement: Additional file 1: — Example of CellPD’s outputs. This folder contains two examples of the outputs generated by CellPD (using the data from Fig. 2 and Additional file 6). (ZIP 36462 kb) [file 12918_2016_337_MOESM1_ESM.zip › USC/output/total/total_loglinear.jpg]

# Total cells model

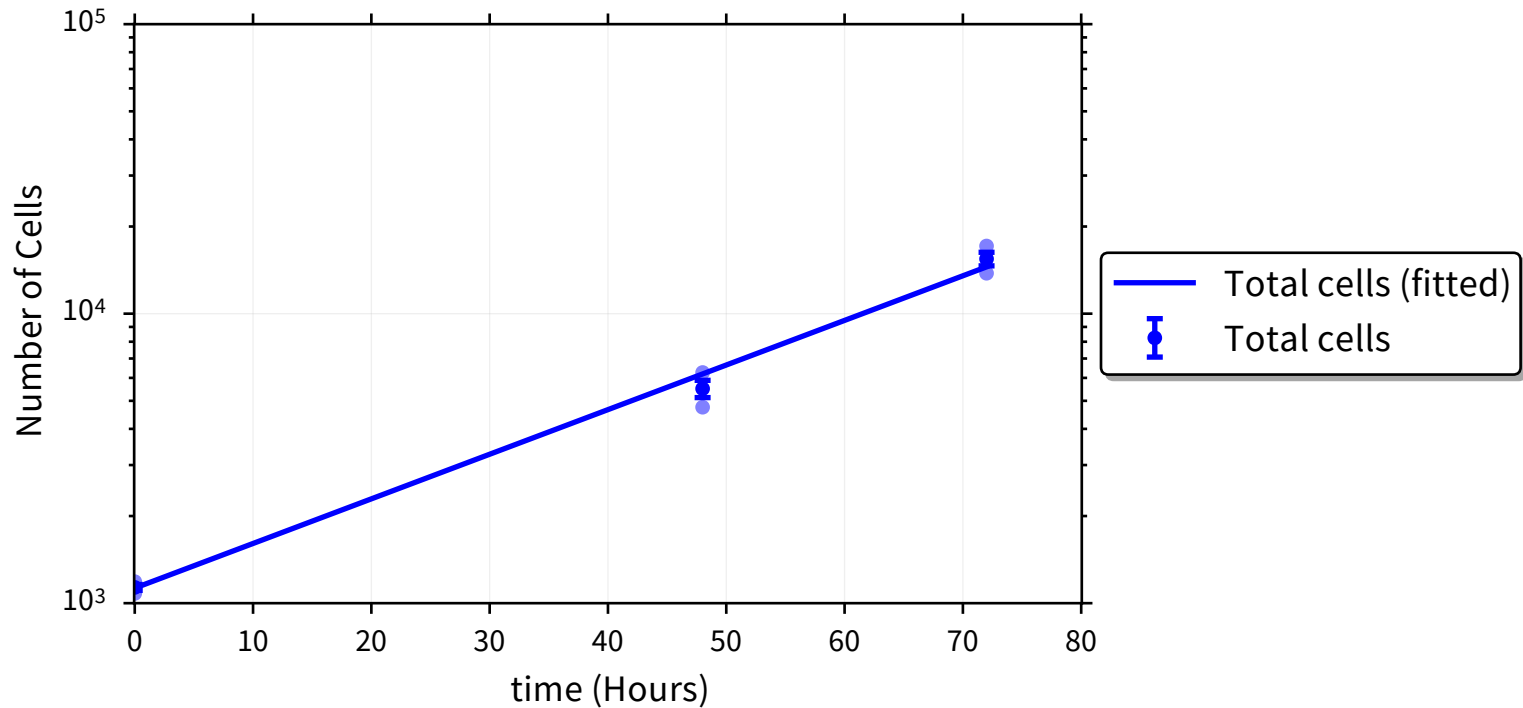

Supplement: Additional file 1: — Example of CellPD’s outputs. This folder contains two examples of the outputs generated by CellPD (using the data from Fig. 2 and Additional file 6). (ZIP 36462 kb) [file 12918_2016_337_MOESM1_ESM.zip › USC/output/total/total_loglinear.pdf]

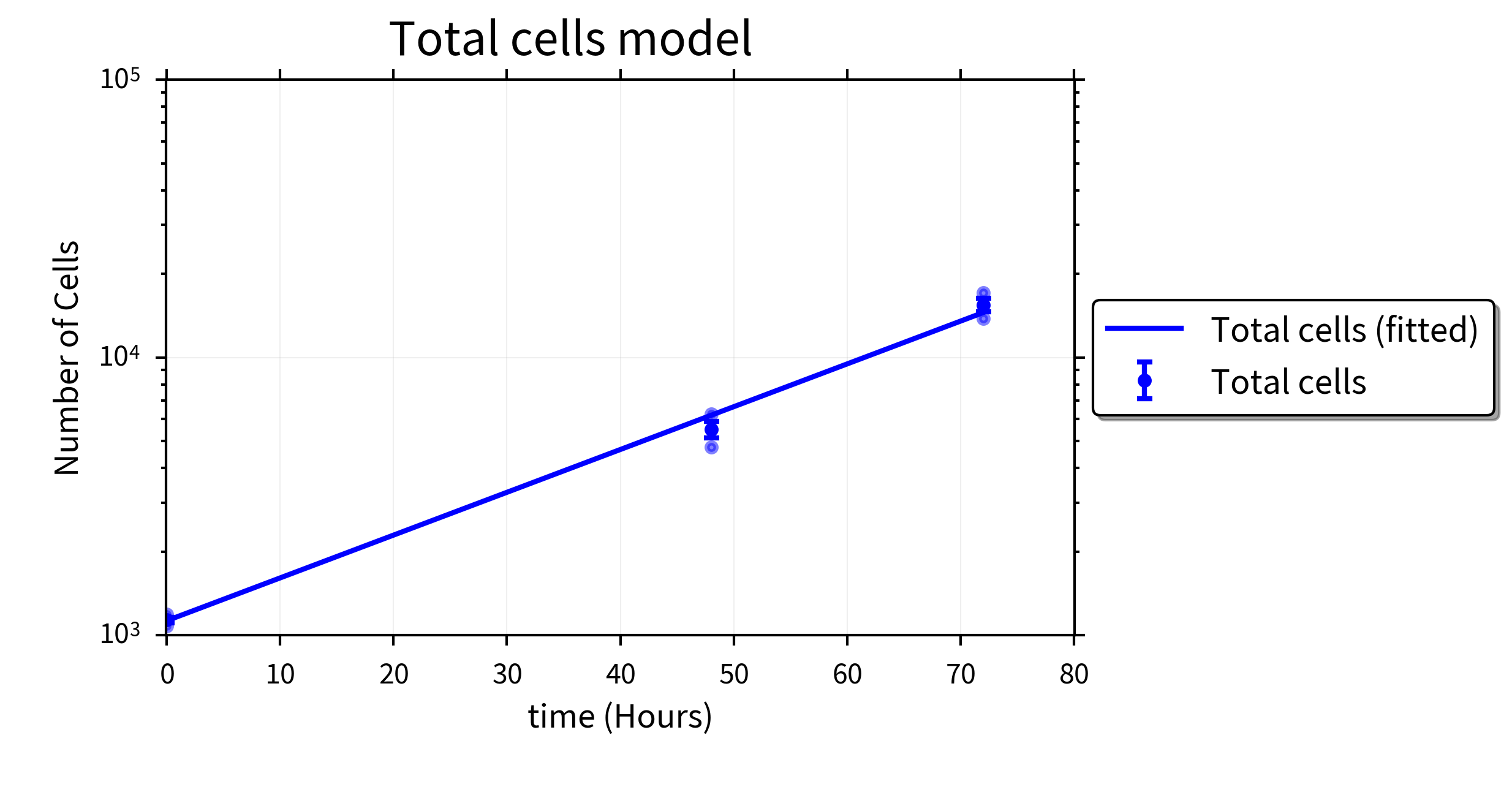

Supplement: Additional file 1: — Example of CellPD’s outputs. This folder contains two examples of the outputs generated by CellPD (using the data from Fig. 2 and Additional file 6). (ZIP 36462 kb) [file 12918_2016_337_MOESM1_ESM.zip › USC/output/total/total_loglinear.png]

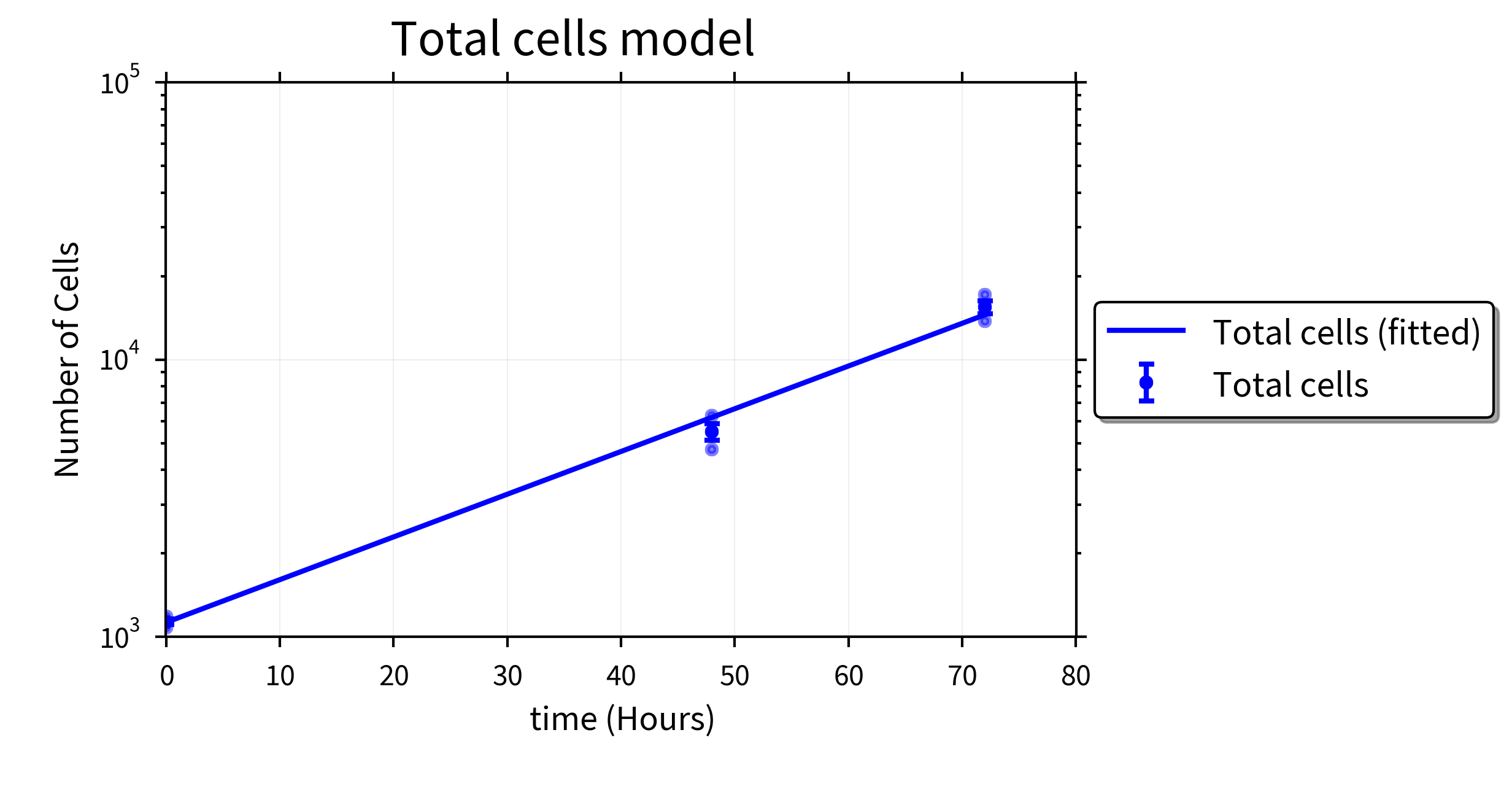

Supplement: Additional file 1: — Example of CellPD’s outputs. This folder contains two examples of the outputs generated by CellPD (using the data from Fig. 2 and Additional file 6). (ZIP 36462 kb) [file 12918_2016_337_MOESM1_ESM.zip › USC/output/total/total_loglinear.tiff]

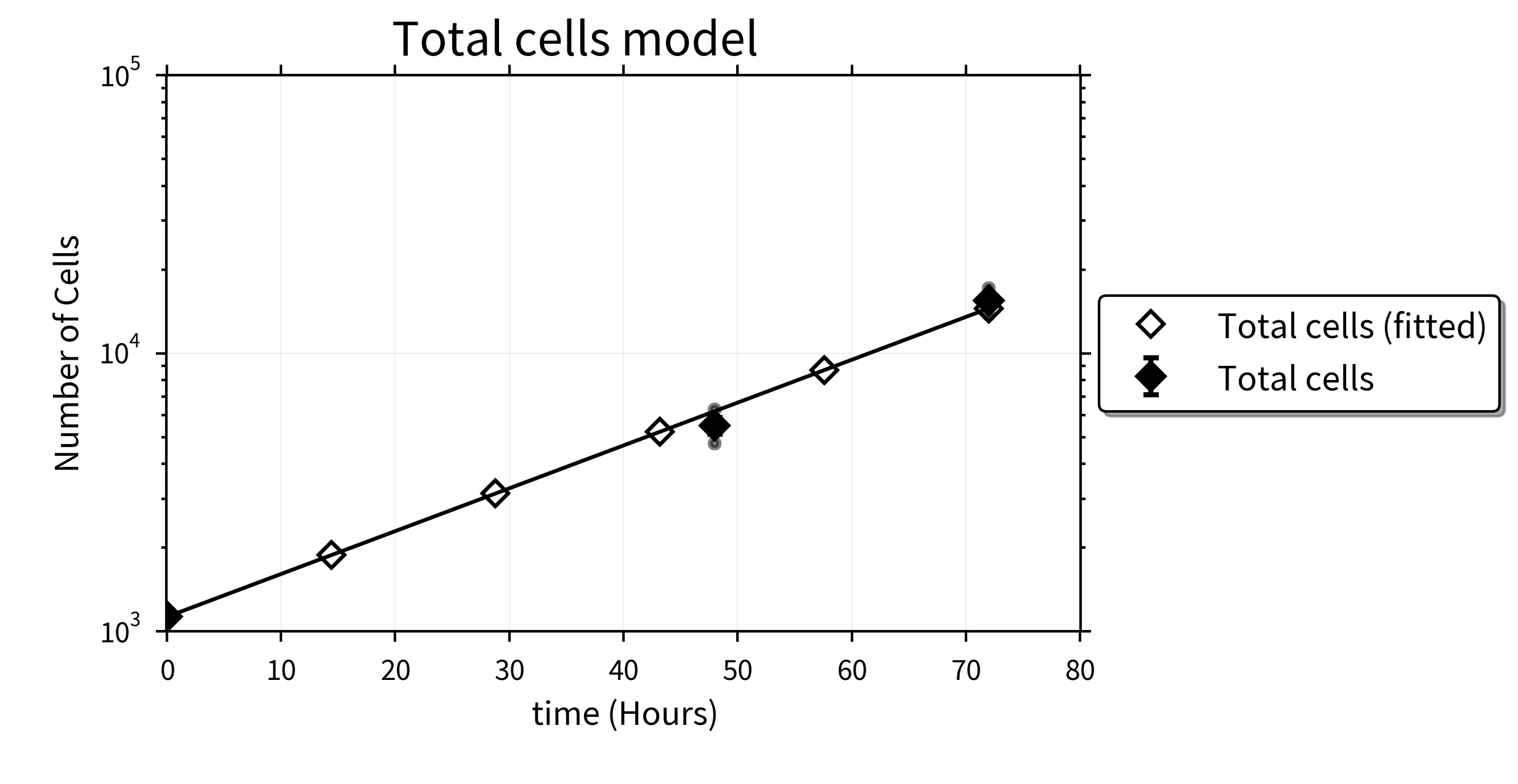

Supplement: Additional file 1: — Example of CellPD’s outputs. This folder contains two examples of the outputs generated by CellPD (using the data from Fig. 2 and Additional file 6). (ZIP 36462 kb) [file 12918_2016_337_MOESM1_ESM.zip › USC/output/total/total_loglinear_BW.jpg]

# Total cells model

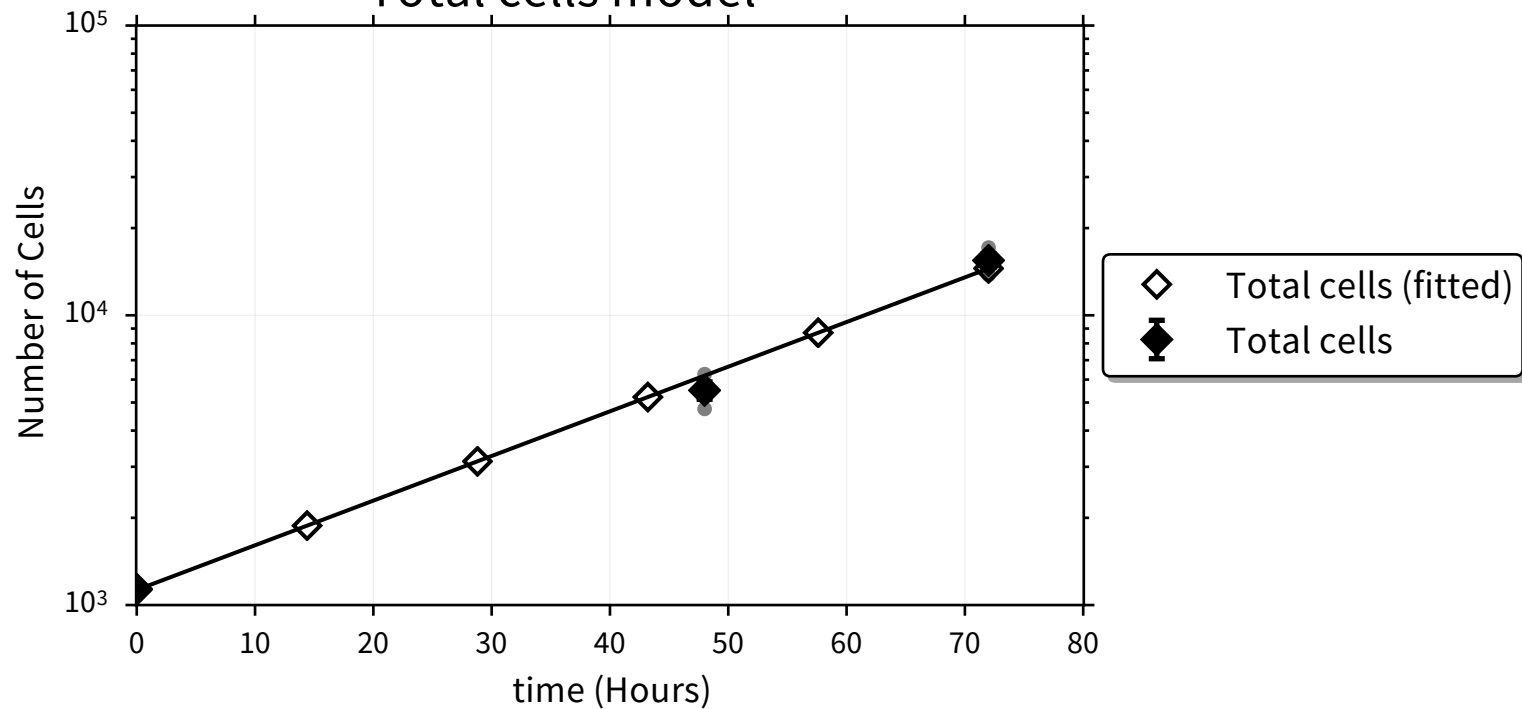

Supplement: Additional file 1: — Example of CellPD’s outputs. This folder contains two examples of the outputs generated by CellPD (using the data from Fig. 2 and Additional file 6). (ZIP 36462 kb) [file 12918_2016_337_MOESM1_ESM.zip › USC/output/total/total_loglinear_BW.pdf]

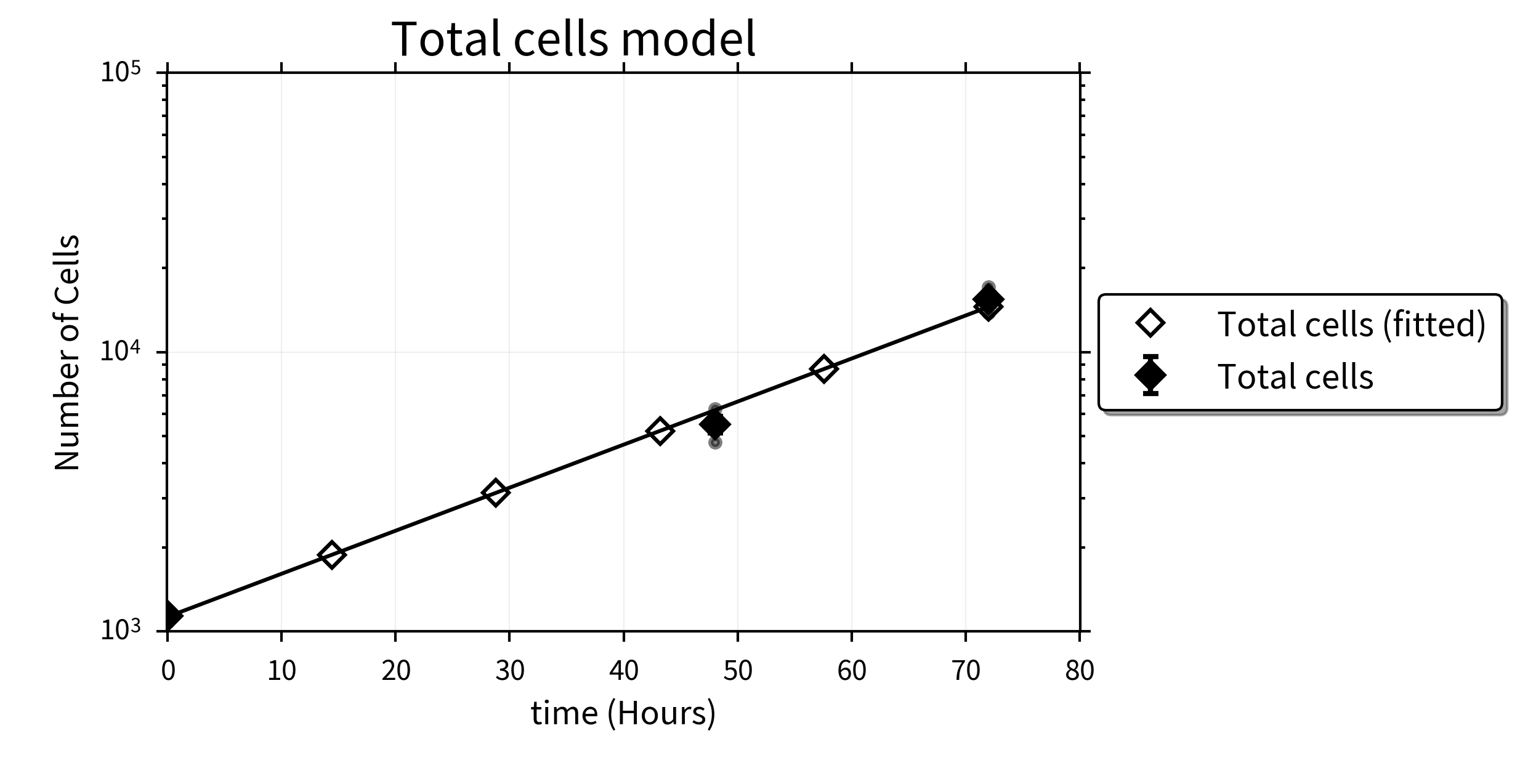

Supplement: Additional file 1: — Example of CellPD’s outputs. This folder contains two examples of the outputs generated by CellPD (using the data from Fig. 2 and Additional file 6). (ZIP 36462 kb) [file 12918_2016_337_MOESM1_ESM.zip › USC/output/total/total_loglinear_BW.png]

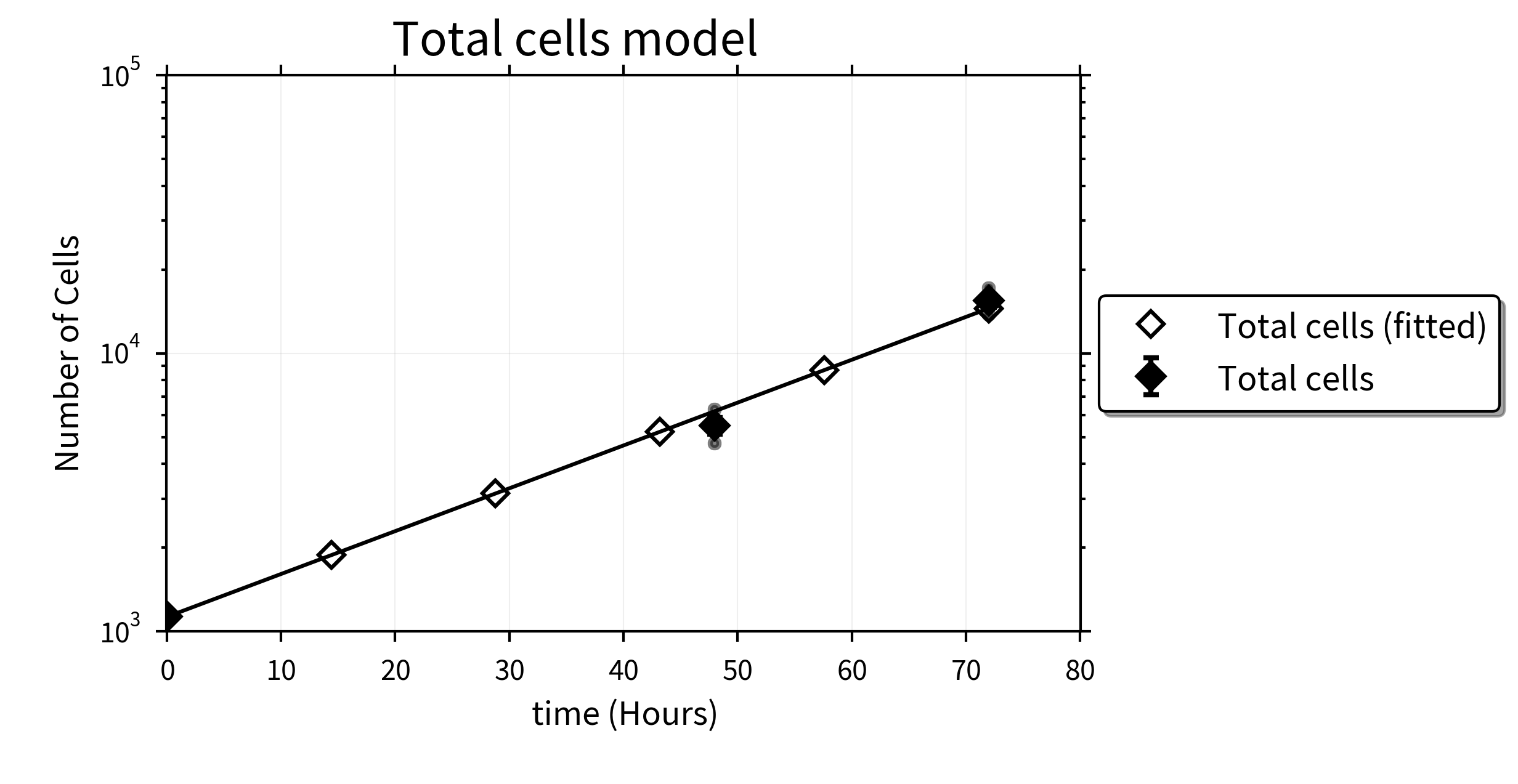

Supplement: Additional file 1: — Example of CellPD’s outputs. This folder contains two examples of the outputs generated by CellPD (using the data from Fig. 2 and Additional file 6). (ZIP 36462 kb) [file 12918_2016_337_MOESM1_ESM.zip › USC/output/total/total_loglinear_BW.tiff]

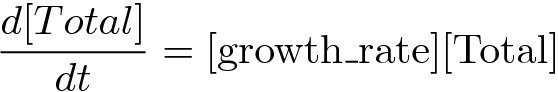

Supplement: Additional file 1: — Example of CellPD’s outputs. This folder contains two examples of the outputs generated by CellPD (using the data from Fig. 2 and Additional file 6). (ZIP 36462 kb) [file 12918_2016_337_MOESM1_ESM.zip › USC/output/total/total_plane.png]

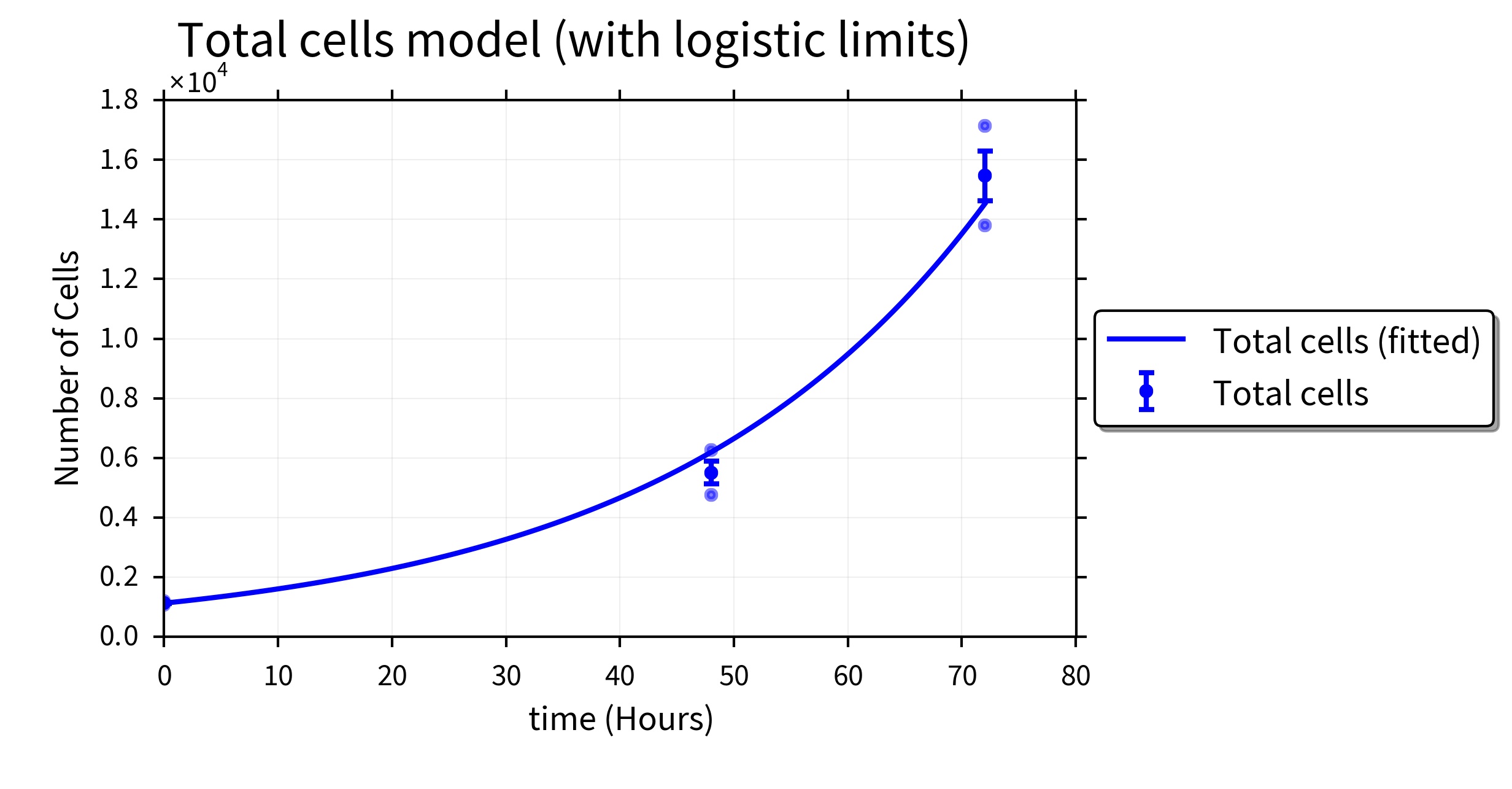

Supplement: Additional file 1: — Example of CellPD’s outputs. This folder contains two examples of the outputs generated by CellPD (using the data from Fig. 2 and Additional file 6). (ZIP 36462 kb) [file 12918_2016_337_MOESM1_ESM.zip › USC/output/total_logistic/total_logistic.jpg]

Total cells model (with logistic limits)

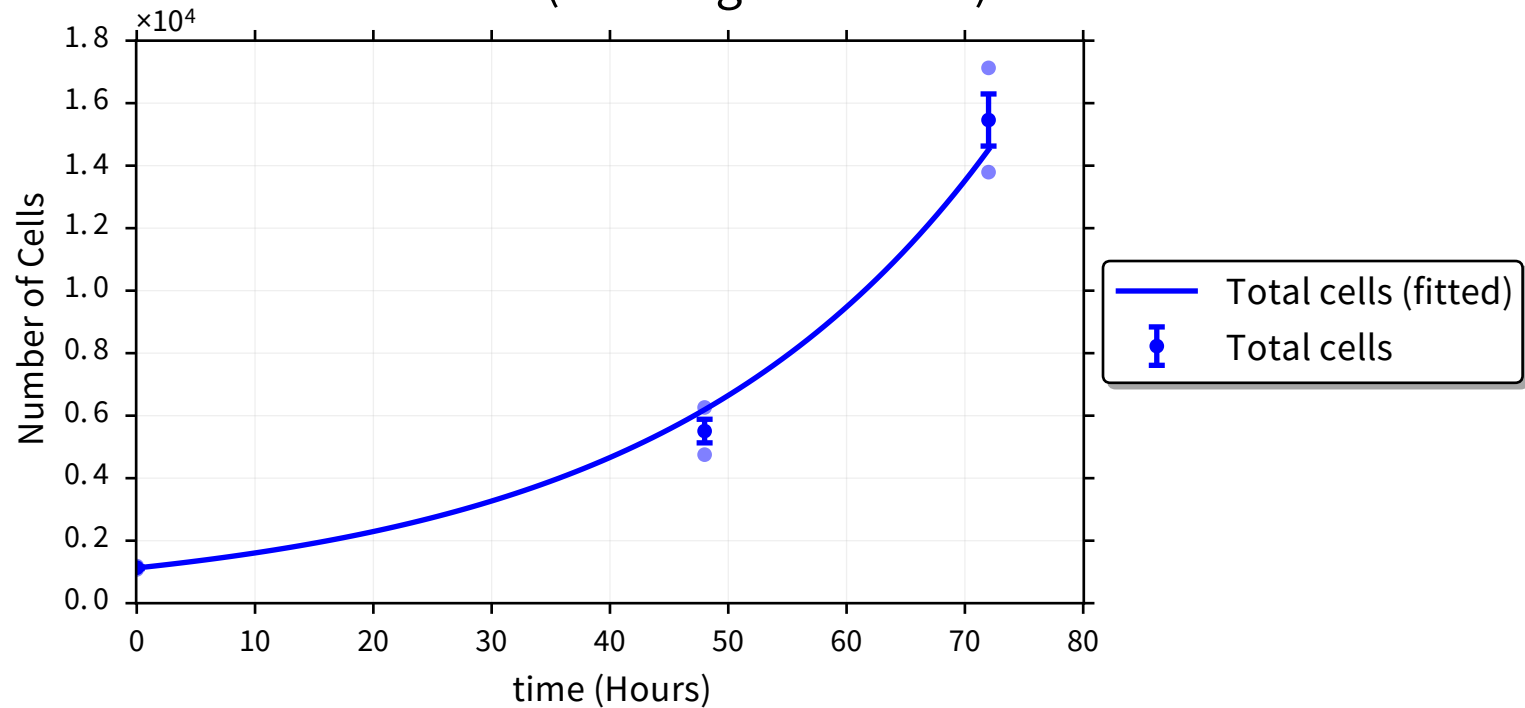

Supplement: Additional file 1: — Example of CellPD’s outputs. This folder contains two examples of the outputs generated by CellPD (using the data from Fig. 2 and Additional file 6). (ZIP 36462 kb) [file 12918_2016_337_MOESM1_ESM.zip › USC/output/total_logistic/total_logistic.pdf]

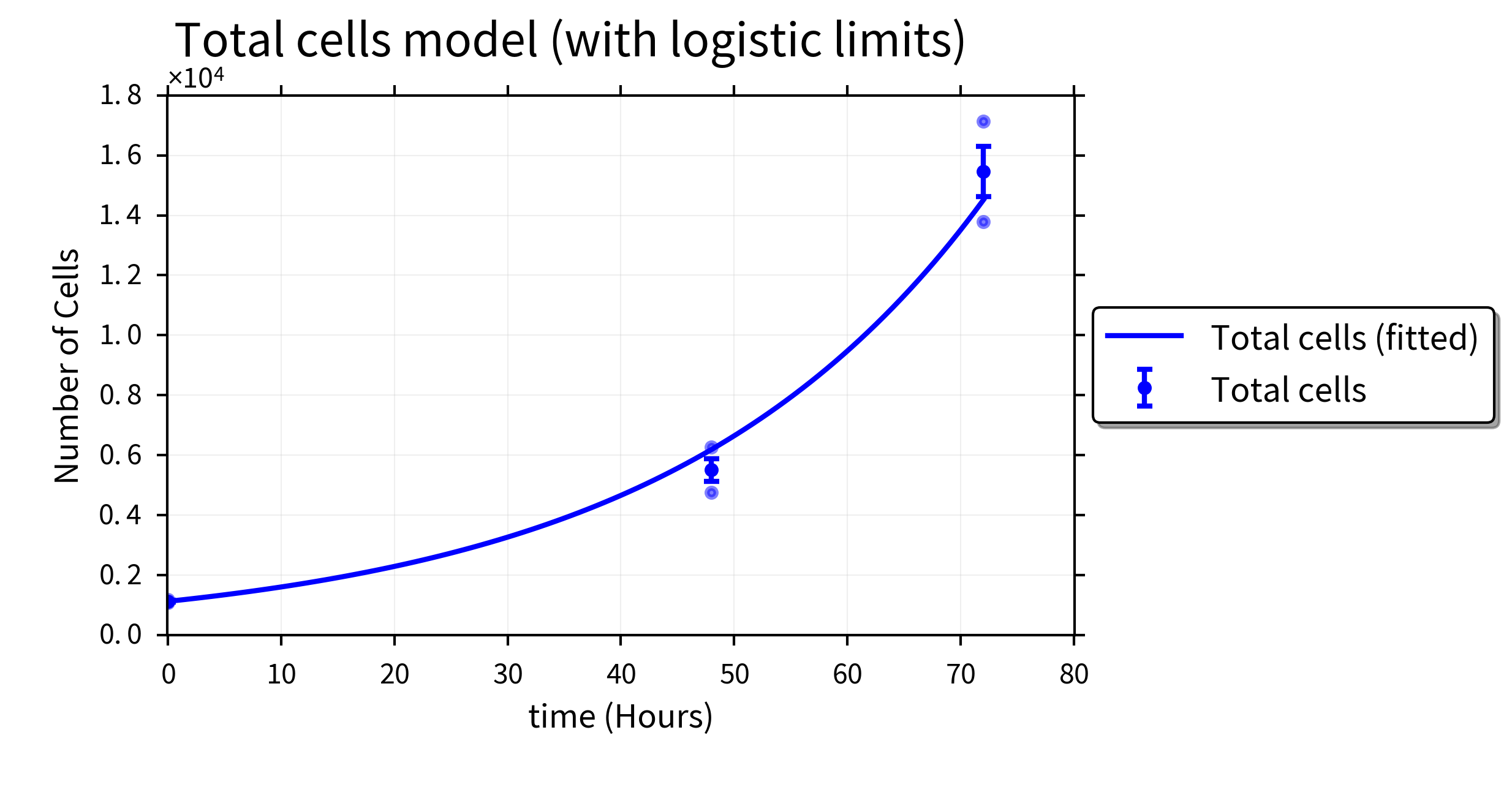

Supplement: Additional file 1: — Example of CellPD’s outputs. This folder contains two examples of the outputs generated by CellPD (using the data from Fig. 2 and Additional file 6). (ZIP 36462 kb) [file 12918_2016_337_MOESM1_ESM.zip › USC/output/total_logistic/total_logistic.png]

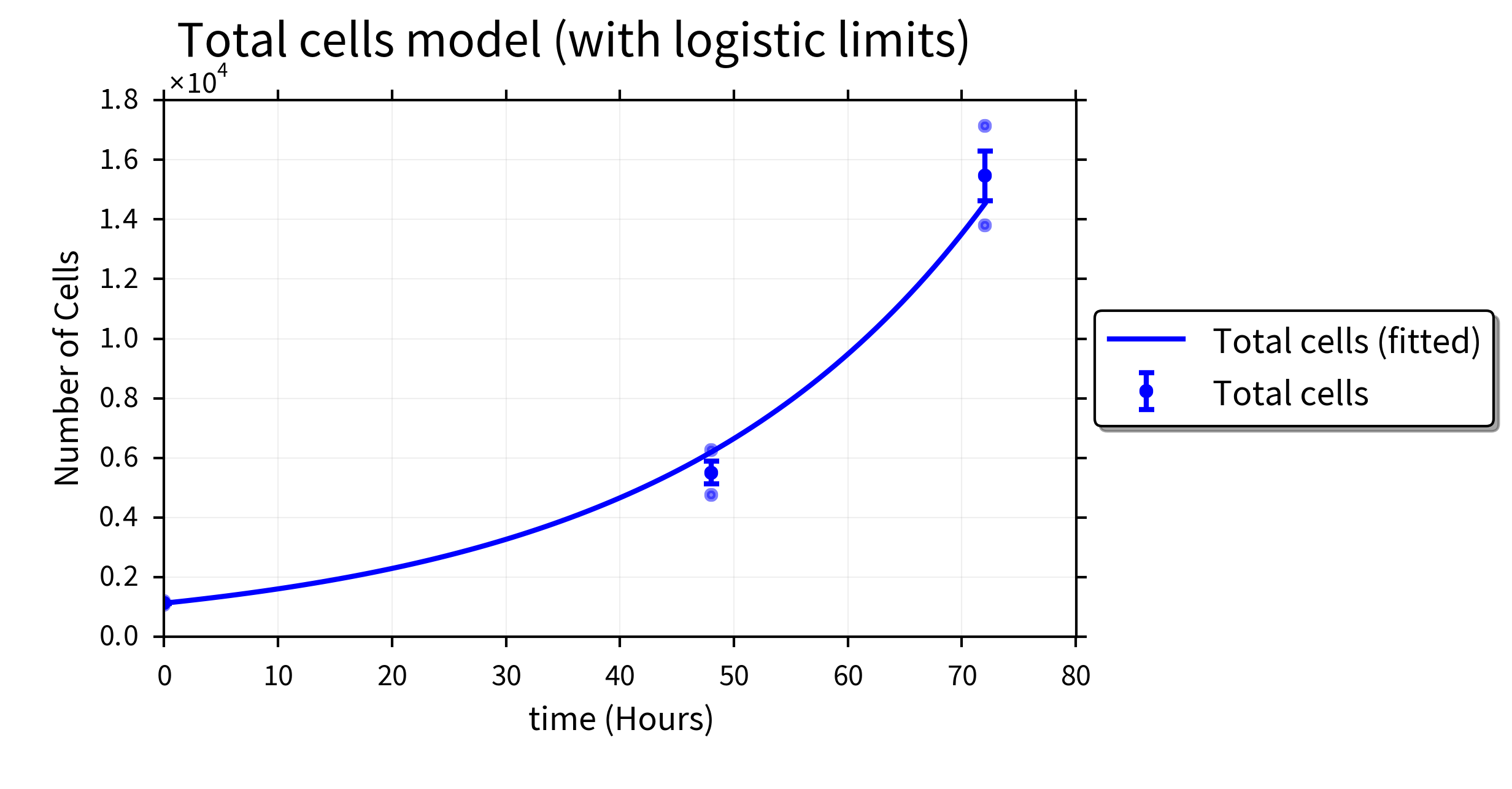

Supplement: Additional file 1: — Example of CellPD’s outputs. This folder contains two examples of the outputs generated by CellPD (using the data from Fig. 2 and Additional file 6). (ZIP 36462 kb) [file 12918_2016_337_MOESM1_ESM.zip › USC/output/total_logistic/total_logistic.tiff]

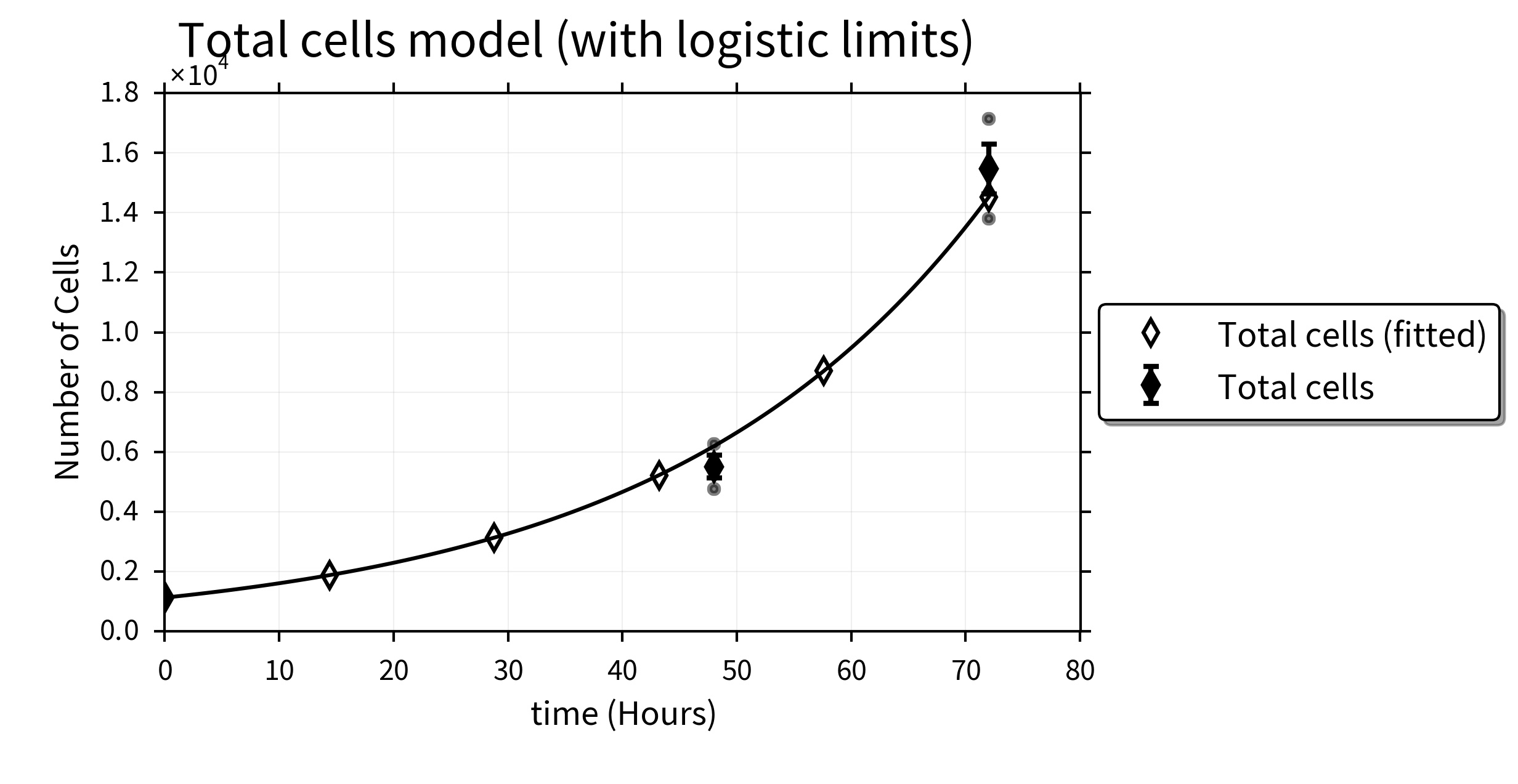

Supplement: Additional file 1: — Example of CellPD’s outputs. This folder contains two examples of the outputs generated by CellPD (using the data from Fig. 2 and Additional file 6). (ZIP 36462 kb) [file 12918_2016_337_MOESM1_ESM.zip › USC/output/total_logistic/total_logistic_BW.jpg]

# Total cells model (with logistic limits)

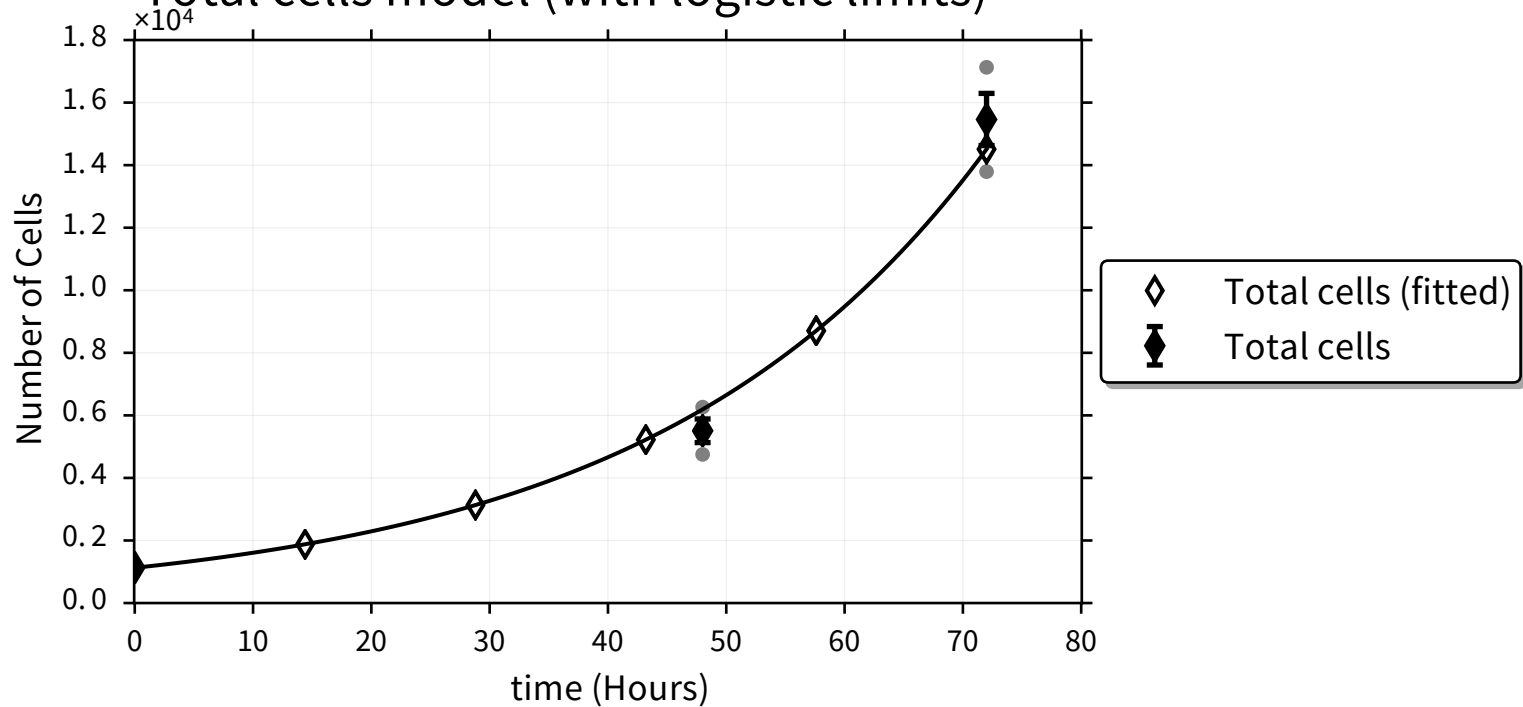

Supplement: Additional file 1: — Example of CellPD’s outputs. This folder contains two examples of the outputs generated by CellPD (using the data from Fig. 2 and Additional file 6). (ZIP 36462 kb) [file 12918_2016_337_MOESM1_ESM.zip › USC/output/total_logistic/total_logistic_BW.pdf]

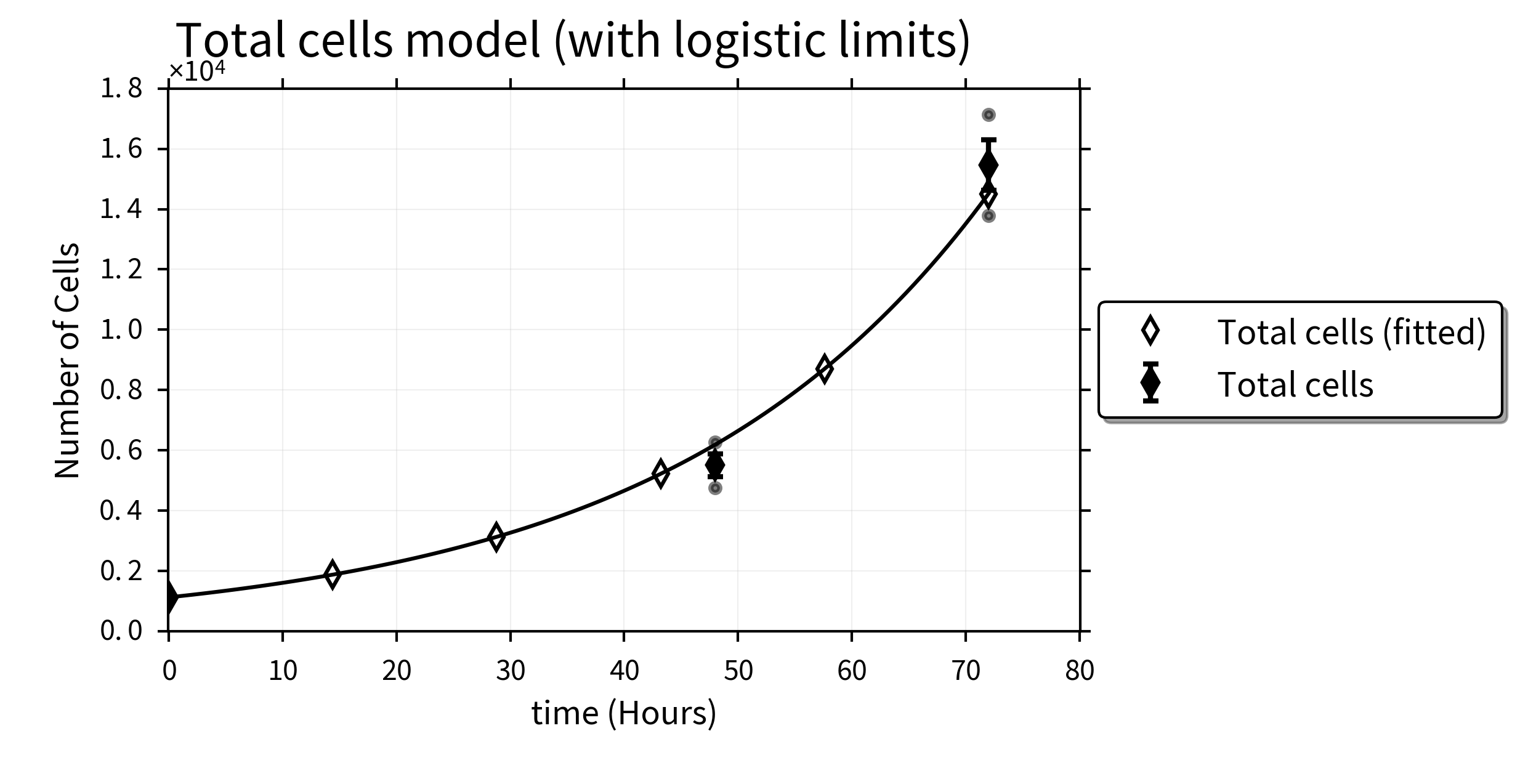

Supplement: Additional file 1: — Example of CellPD’s outputs. This folder contains two examples of the outputs generated by CellPD (using the data from Fig. 2 and Additional file 6). (ZIP 36462 kb) [file 12918_2016_337_MOESM1_ESM.zip › USC/output/total_logistic/total_logistic_BW.png]

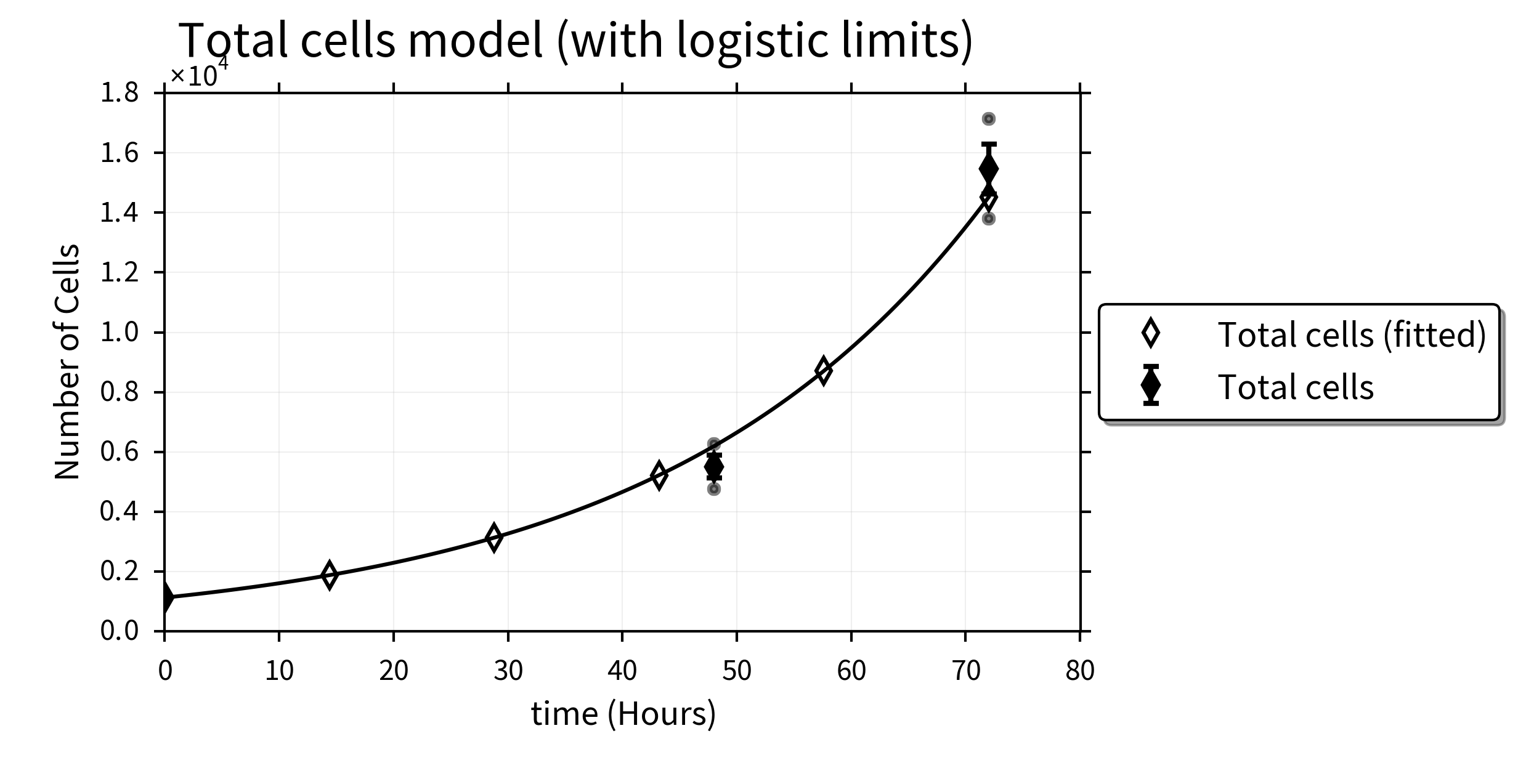

Supplement: Additional file 1: — Example of CellPD’s outputs. This folder contains two examples of the outputs generated by CellPD (using the data from Fig. 2 and Additional file 6). (ZIP 36462 kb) [file 12918_2016_337_MOESM1_ESM.zip › USC/output/total_logistic/total_logistic_BW.tiff]

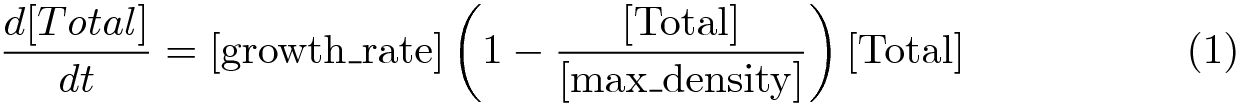

Supplement: Additional file 1: — Example of CellPD’s outputs. This folder contains two examples of the outputs generated by CellPD (using the data from Fig. 2 and Additional file 6). (ZIP 36462 kb) [file 12918_2016_337_MOESM1_ESM.zip › USC/output/total_logistic/total_logistic_eqn.png]

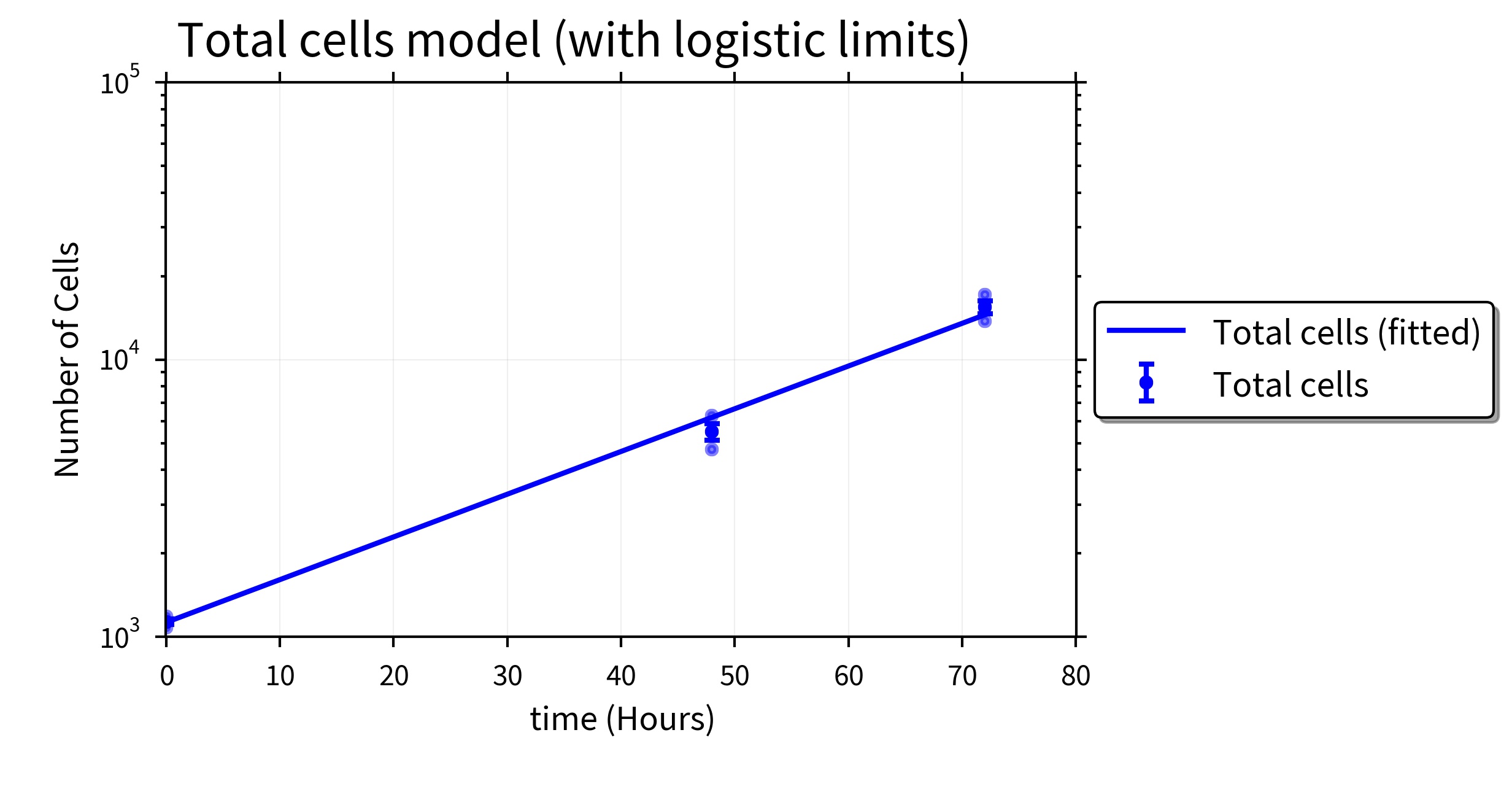

Supplement: Additional file 1: — Example of CellPD’s outputs. This folder contains two examples of the outputs generated by CellPD (using the data from Fig. 2 and Additional file 6). (ZIP 36462 kb) [file 12918_2016_337_MOESM1_ESM.zip › USC/output/total_logistic/total_logistic_loglinear.jpg]

Total cells model (with logistic limits)

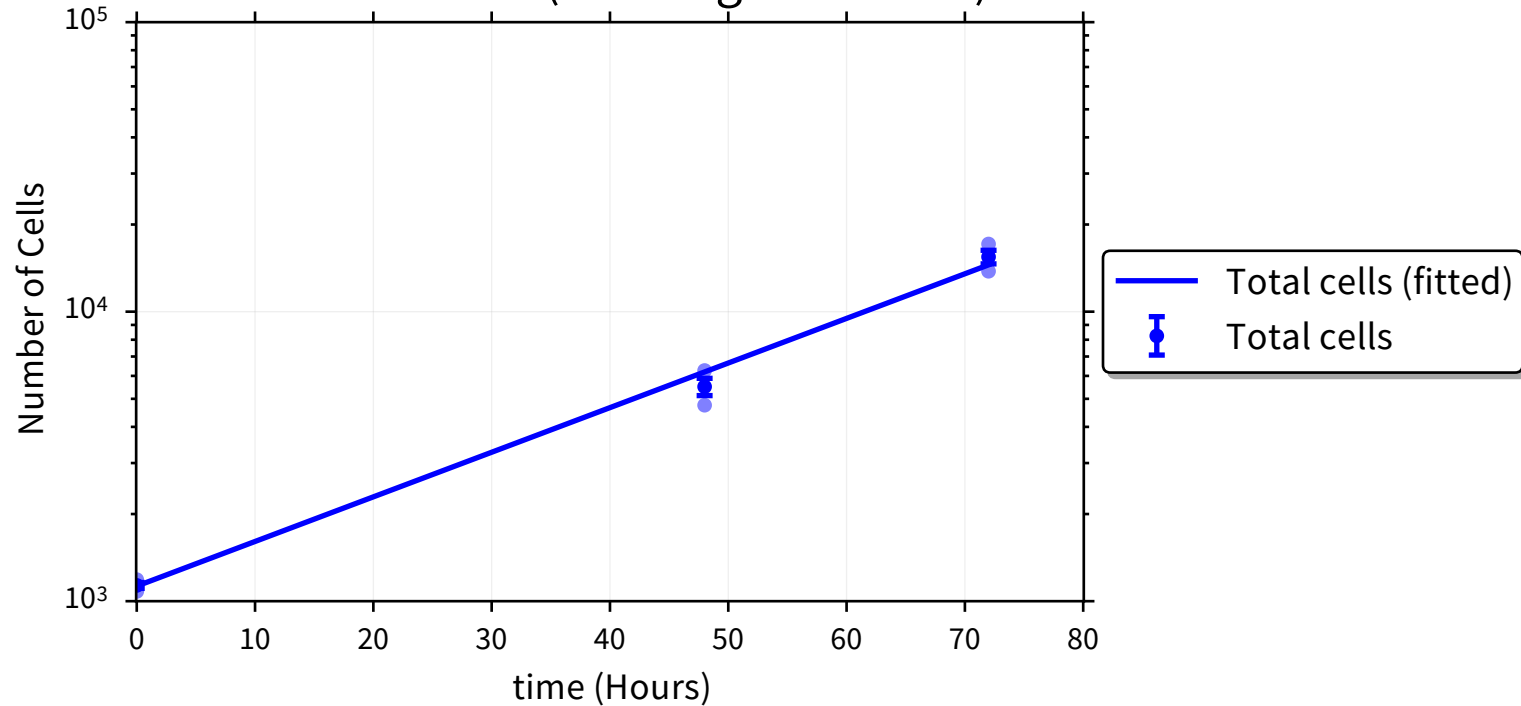

Supplement: Additional file 1: — Example of CellPD’s outputs. This folder contains two examples of the outputs generated by CellPD (using the data from Fig. 2 and Additional file 6). (ZIP 36462 kb) [file 12918_2016_337_MOESM1_ESM.zip › USC/output/total_logistic/total_logistic_loglinear.pdf]

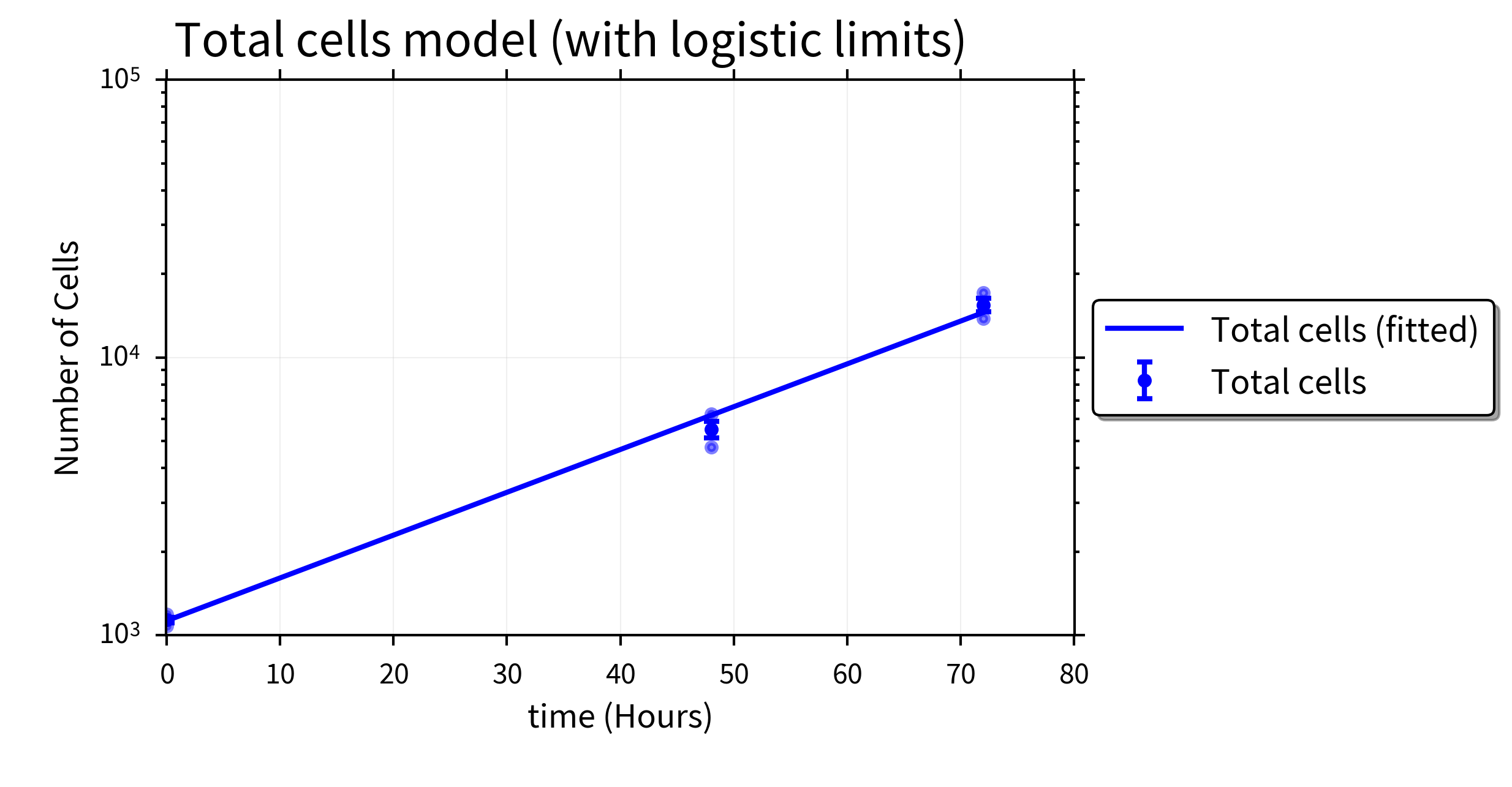

Supplement: Additional file 1: — Example of CellPD’s outputs. This folder contains two examples of the outputs generated by CellPD (using the data from Fig. 2 and Additional file 6). (ZIP 36462 kb) [file 12918_2016_337_MOESM1_ESM.zip › USC/output/total_logistic/total_logistic_loglinear.png]

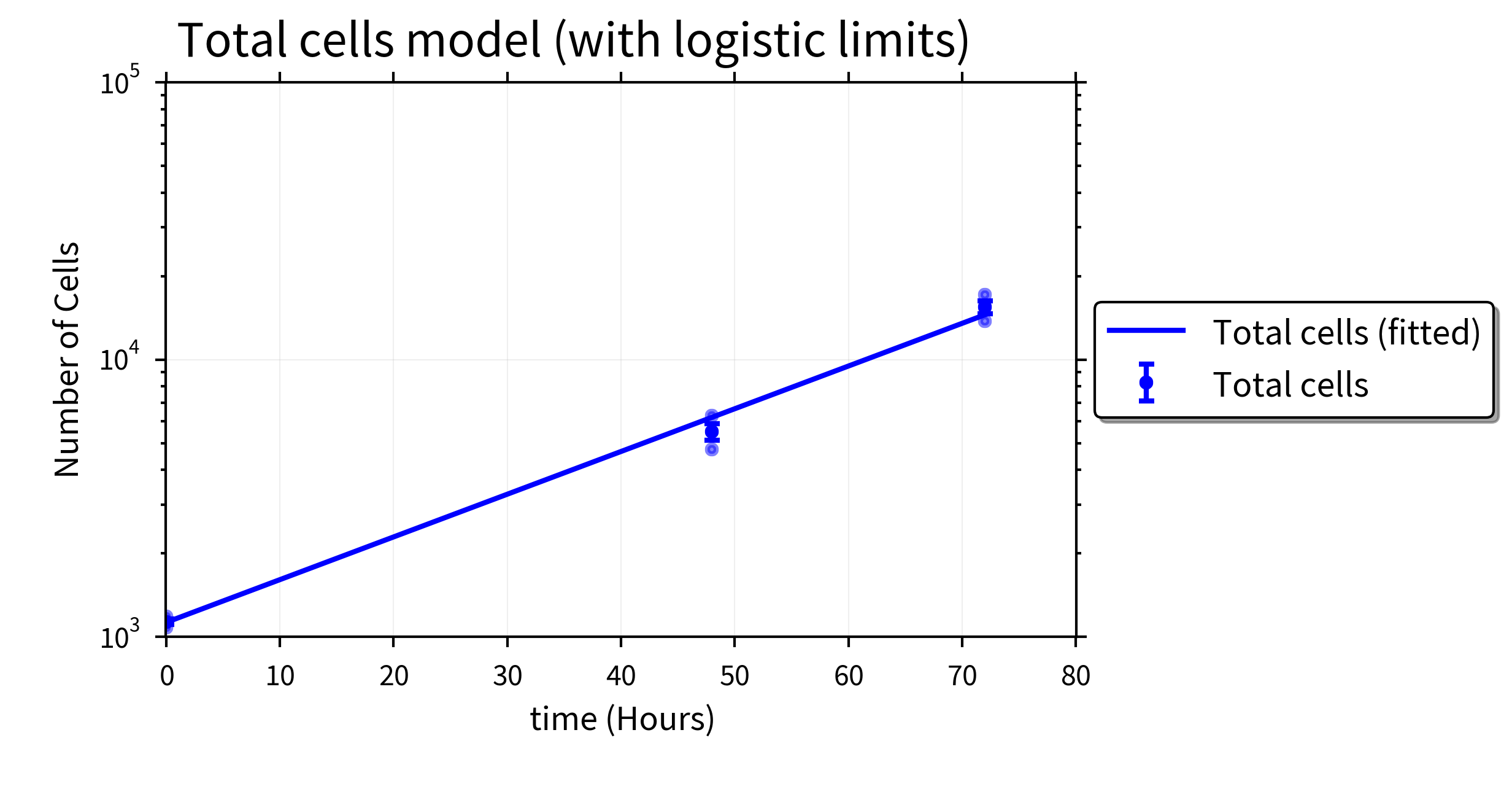

Supplement: Additional file 1: — Example of CellPD’s outputs. This folder contains two examples of the outputs generated by CellPD (using the data from Fig. 2 and Additional file 6). (ZIP 36462 kb) [file 12918_2016_337_MOESM1_ESM.zip › USC/output/total_logistic/total_logistic_loglinear.tiff]

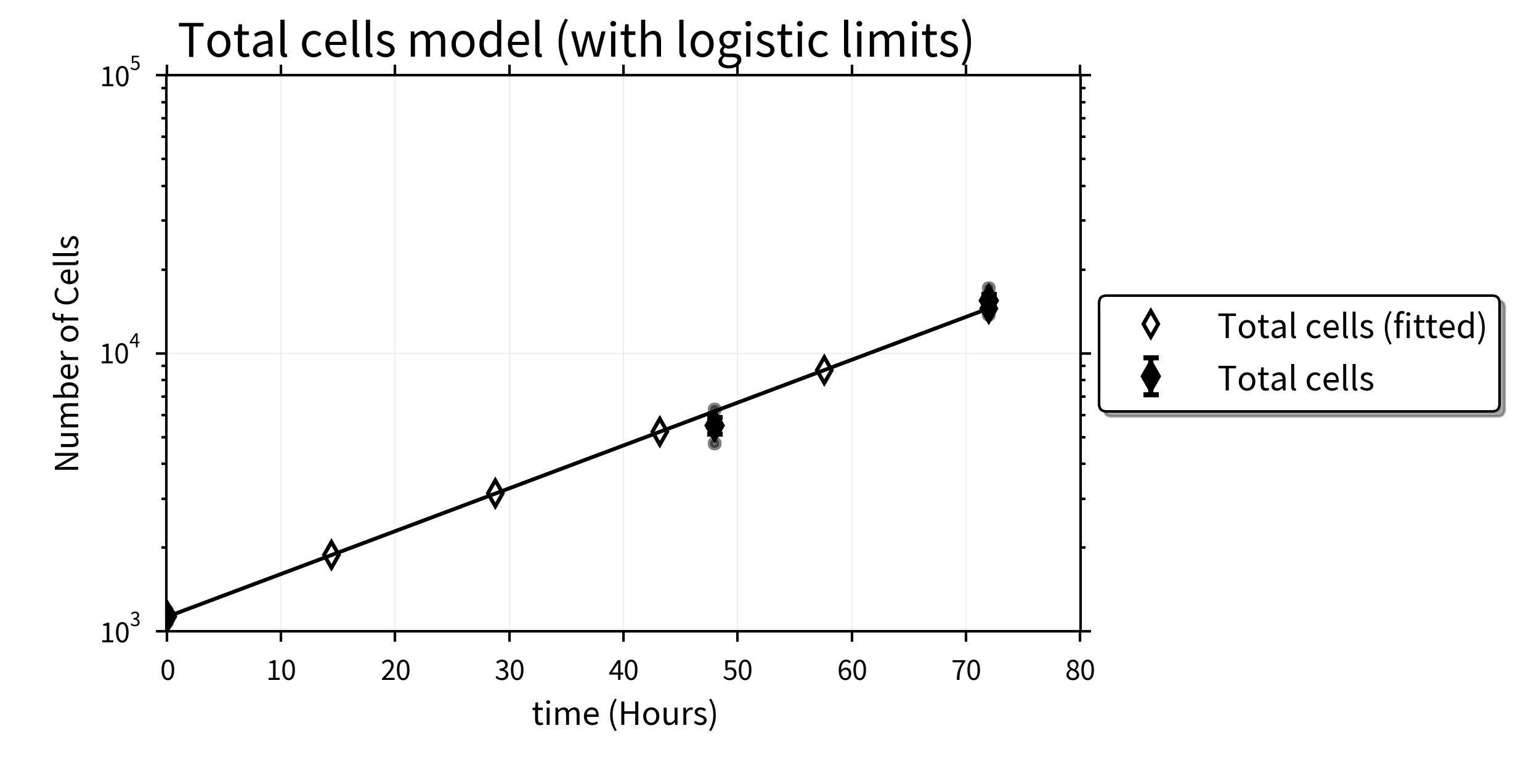

Supplement: Additional file 1: — Example of CellPD’s outputs. This folder contains two examples of the outputs generated by CellPD (using the data from Fig. 2 and Additional file 6). (ZIP 36462 kb) [file 12918_2016_337_MOESM1_ESM.zip › USC/output/total_logistic/total_logistic_loglinear_BW.jpg]

Total cells model (with logistic limits)

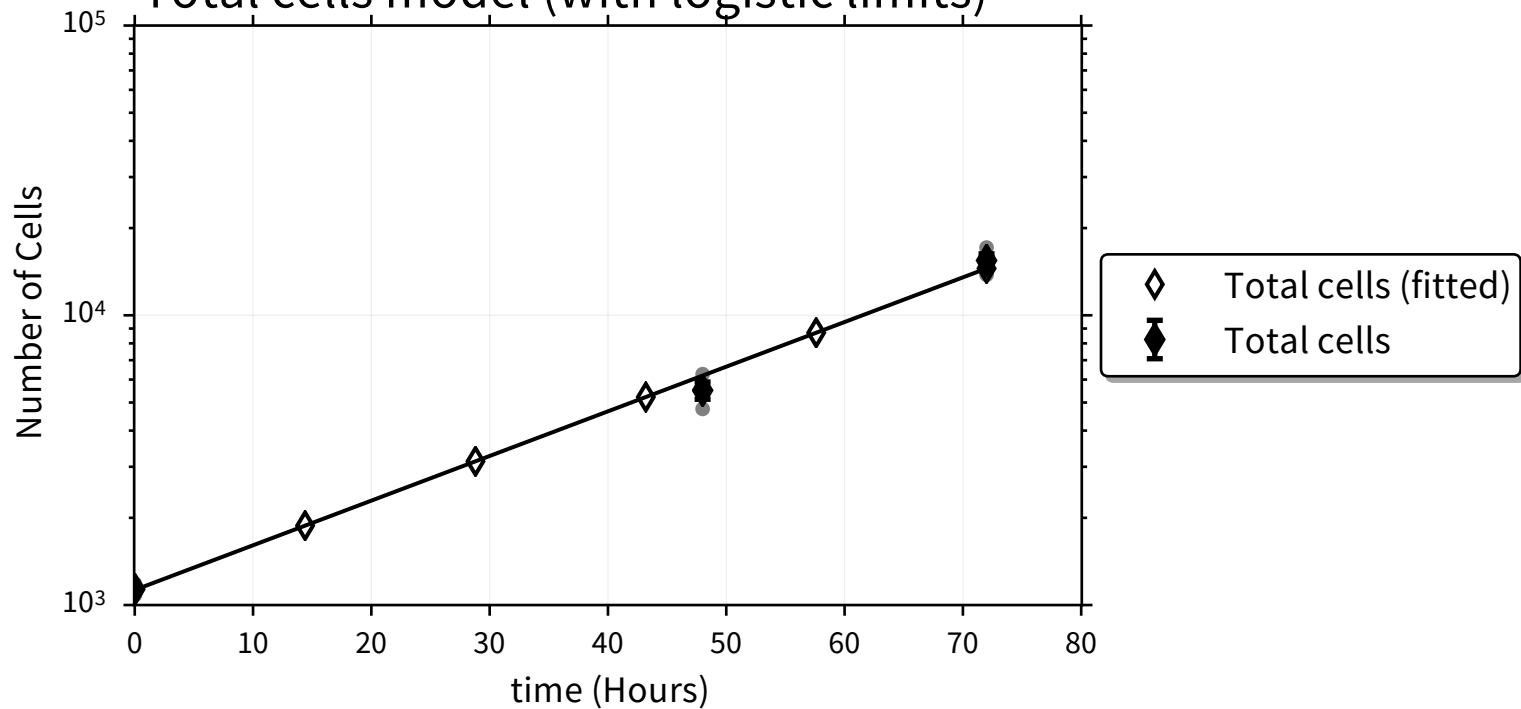

Supplement: Additional file 1: — Example of CellPD’s outputs. This folder contains two examples of the outputs generated by CellPD (using the data from Fig. 2 and Additional file 6). (ZIP 36462 kb) [file 12918_2016_337_MOESM1_ESM.zip › USC/output/total_logistic/total_logistic_loglinear_BW.pdf]

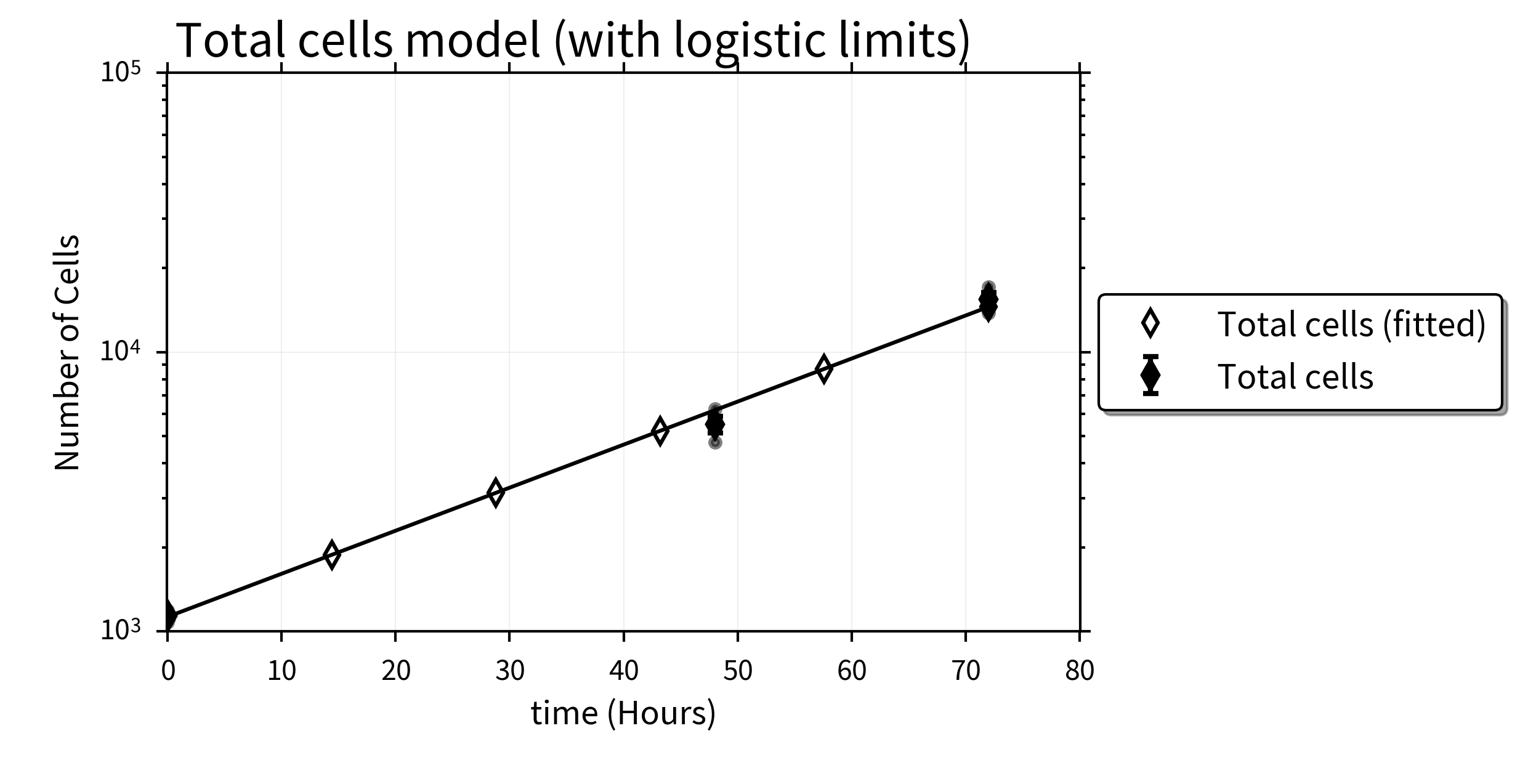

Supplement: Additional file 1: — Example of CellPD’s outputs. This folder contains two examples of the outputs generated by CellPD (using the data from Fig. 2 and Additional file 6). (ZIP 36462 kb) [file 12918_2016_337_MOESM1_ESM.zip › USC/output/total_logistic/total_logistic_loglinear_BW.png]

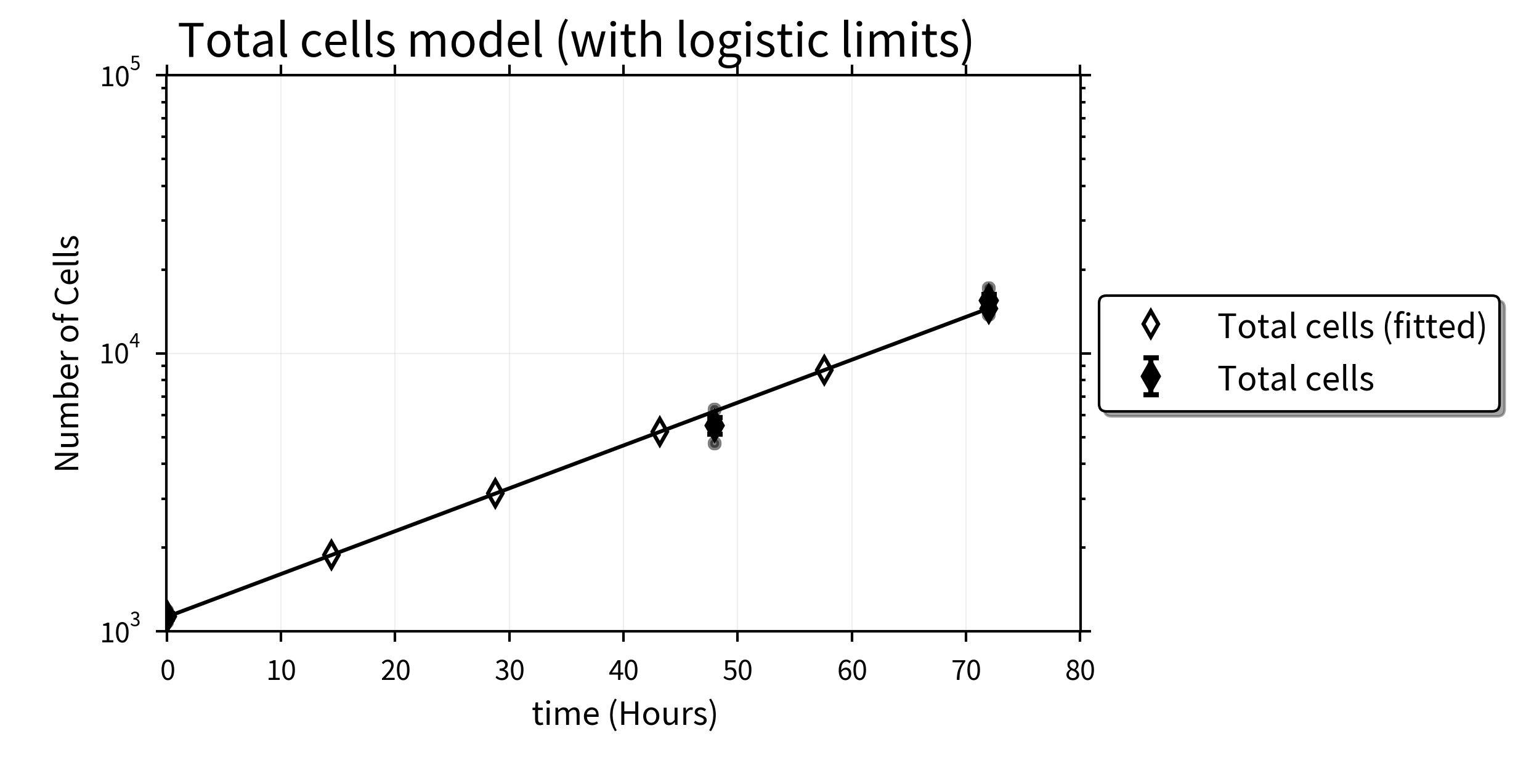

Supplement: Additional file 1: — Example of CellPD’s outputs. This folder contains two examples of the outputs generated by CellPD (using the data from Fig. 2 and Additional file 6). (ZIP 36462 kb) [file 12918_2016_337_MOESM1_ESM.zip › USC/output/total_logistic/total_logistic_loglinear_BW.tiff]

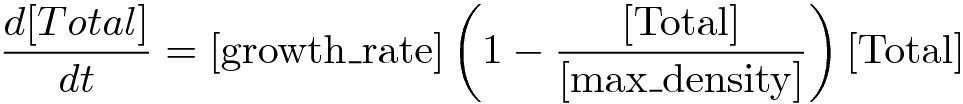

Supplement: Additional file 1: — Example of CellPD’s outputs. This folder contains two examples of the outputs generated by CellPD (using the data from Fig. 2 and Additional file 6). (ZIP 36462 kb) [file 12918_2016_337_MOESM1_ESM.zip › USC/output/total_logistic/total_logistic_plane.png]

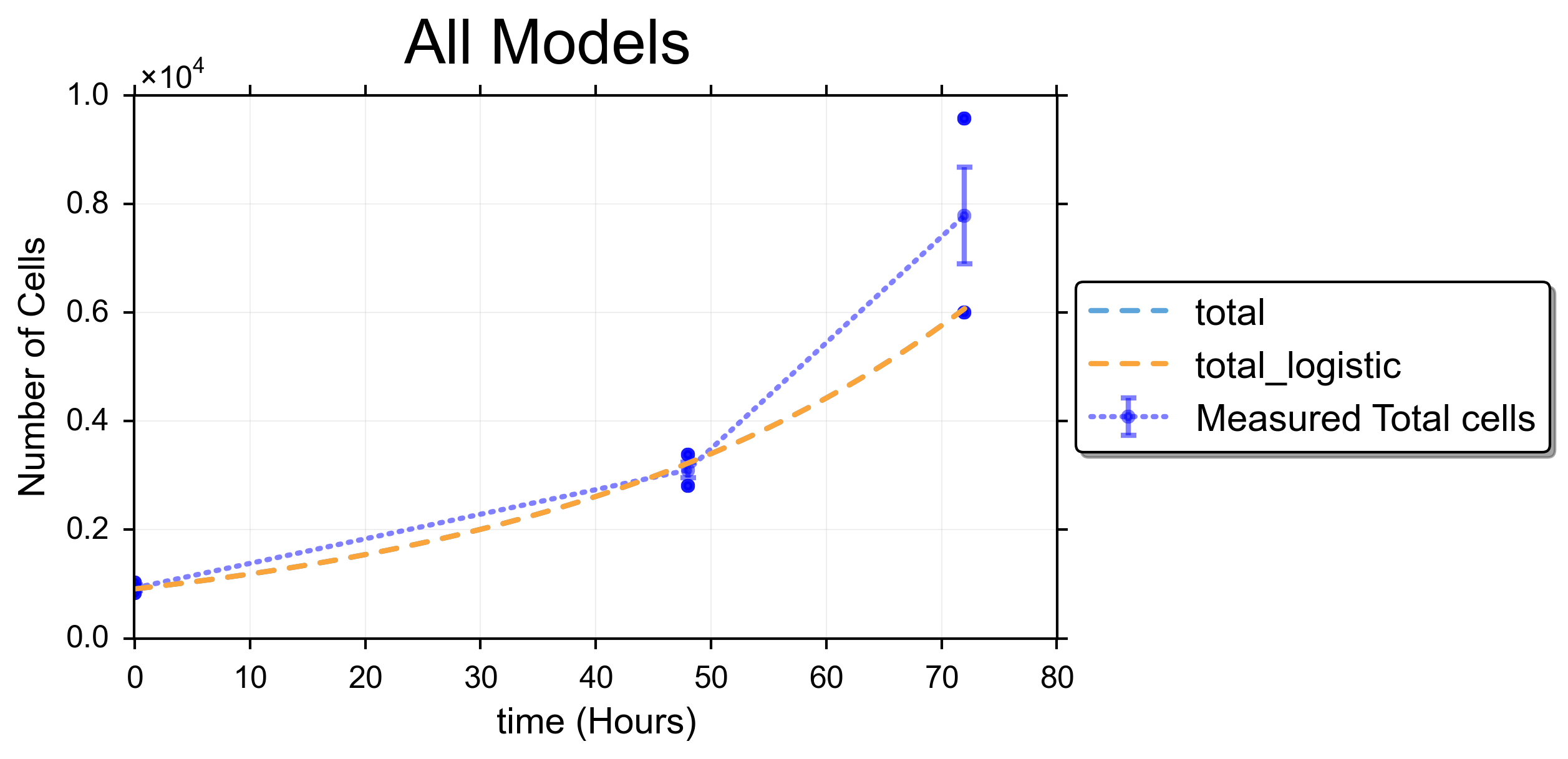

Supplement: Additional file 1: — Example of CellPD’s outputs. This folder contains two examples of the outputs generated by CellPD (using the data from Fig. 2 and Additional file 6). (ZIP 36462 kb) [file 12918_2016_337_MOESM1_ESM.zip › WFU/output/data/All_Models.png]

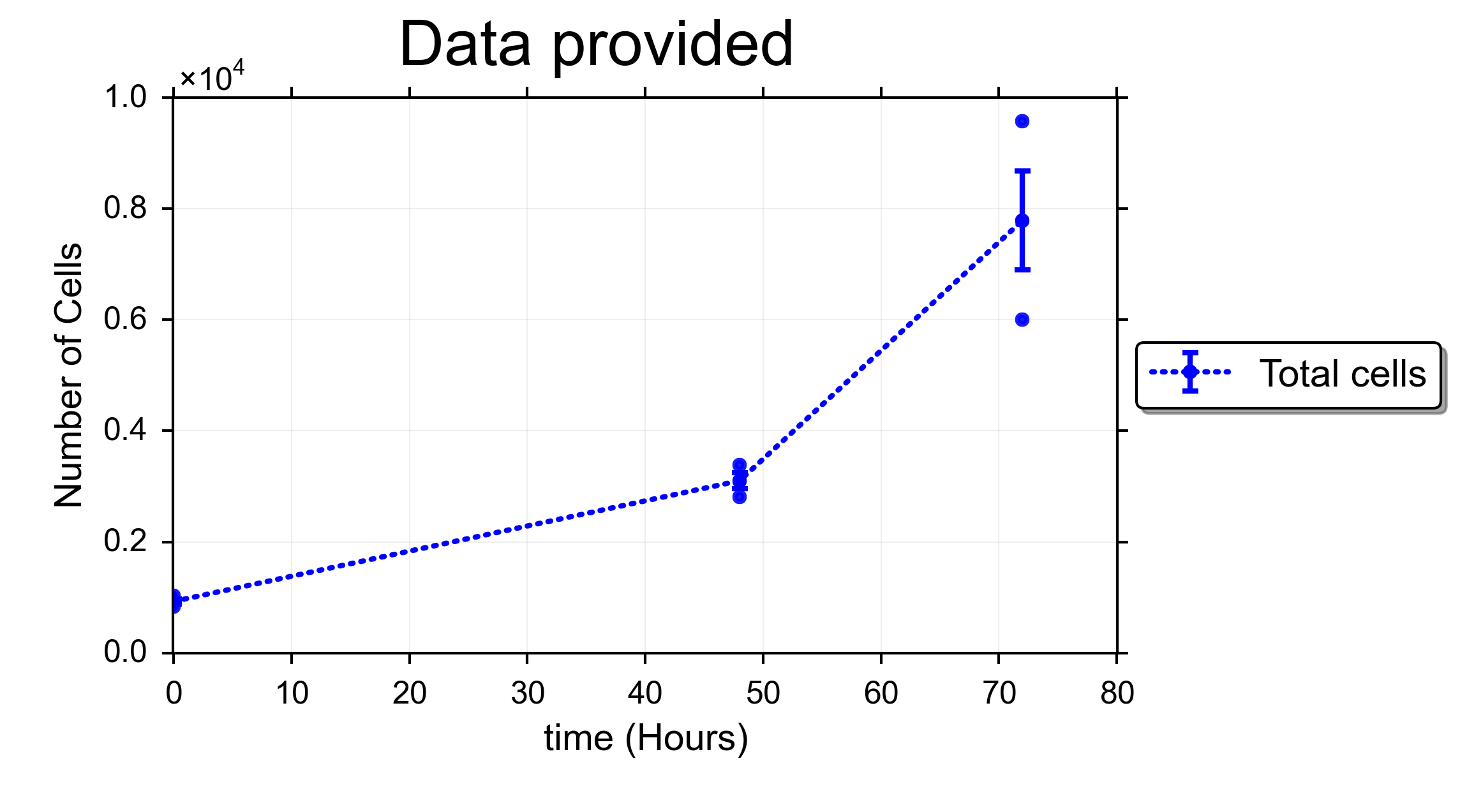

Supplement: Additional file 1: — Example of CellPD’s outputs. This folder contains two examples of the outputs generated by CellPD (using the data from Fig. 2 and Additional file 6). (ZIP 36462 kb) [file 12918_2016_337_MOESM1_ESM.zip › WFU/output/data/Data.png]

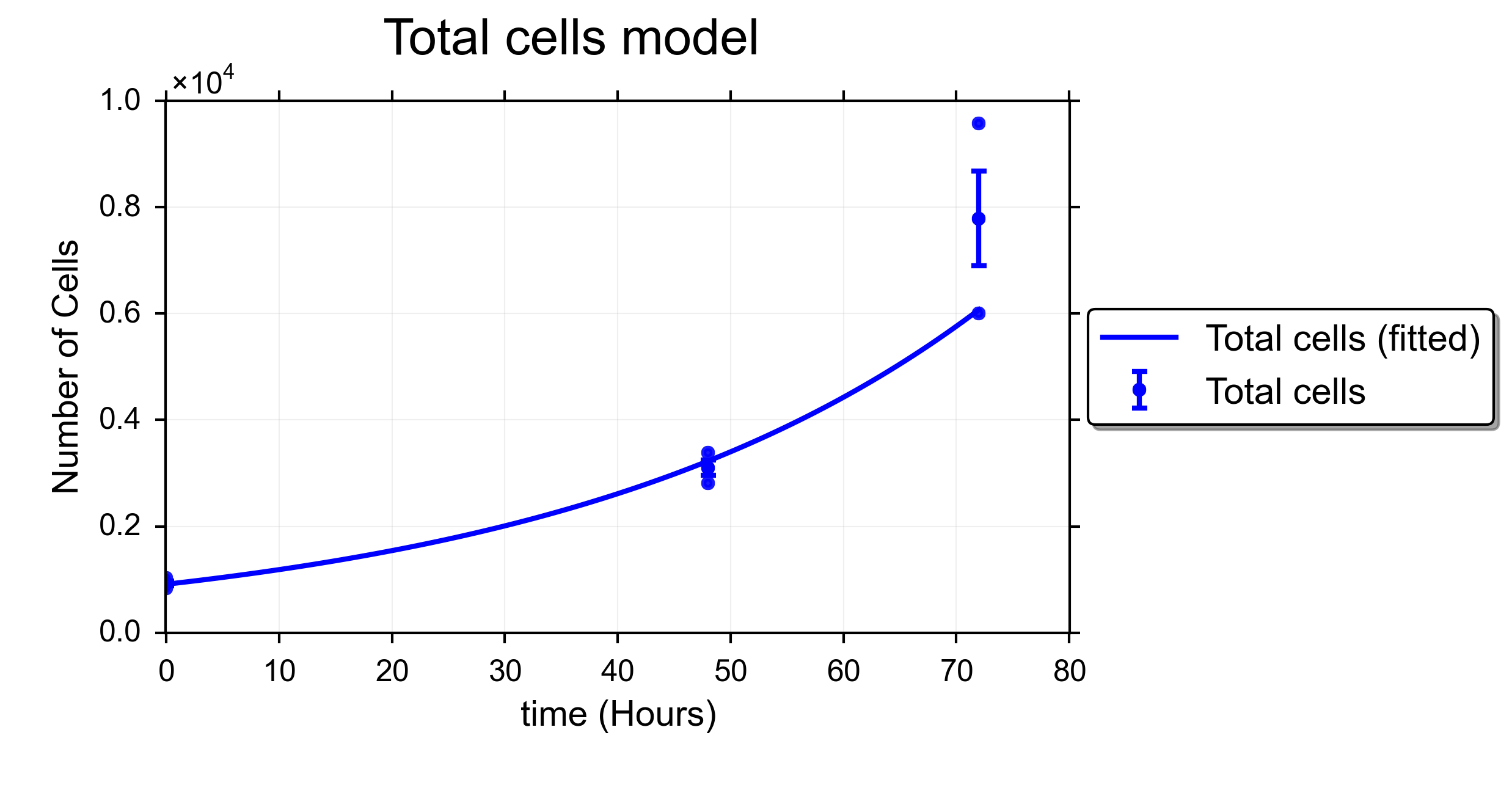

Supplement: Additional file 1: — Example of CellPD’s outputs. This folder contains two examples of the outputs generated by CellPD (using the data from Fig. 2 and Additional file 6). (ZIP 36462 kb) [file 12918_2016_337_MOESM1_ESM.zip › WFU/output/total/total.png]

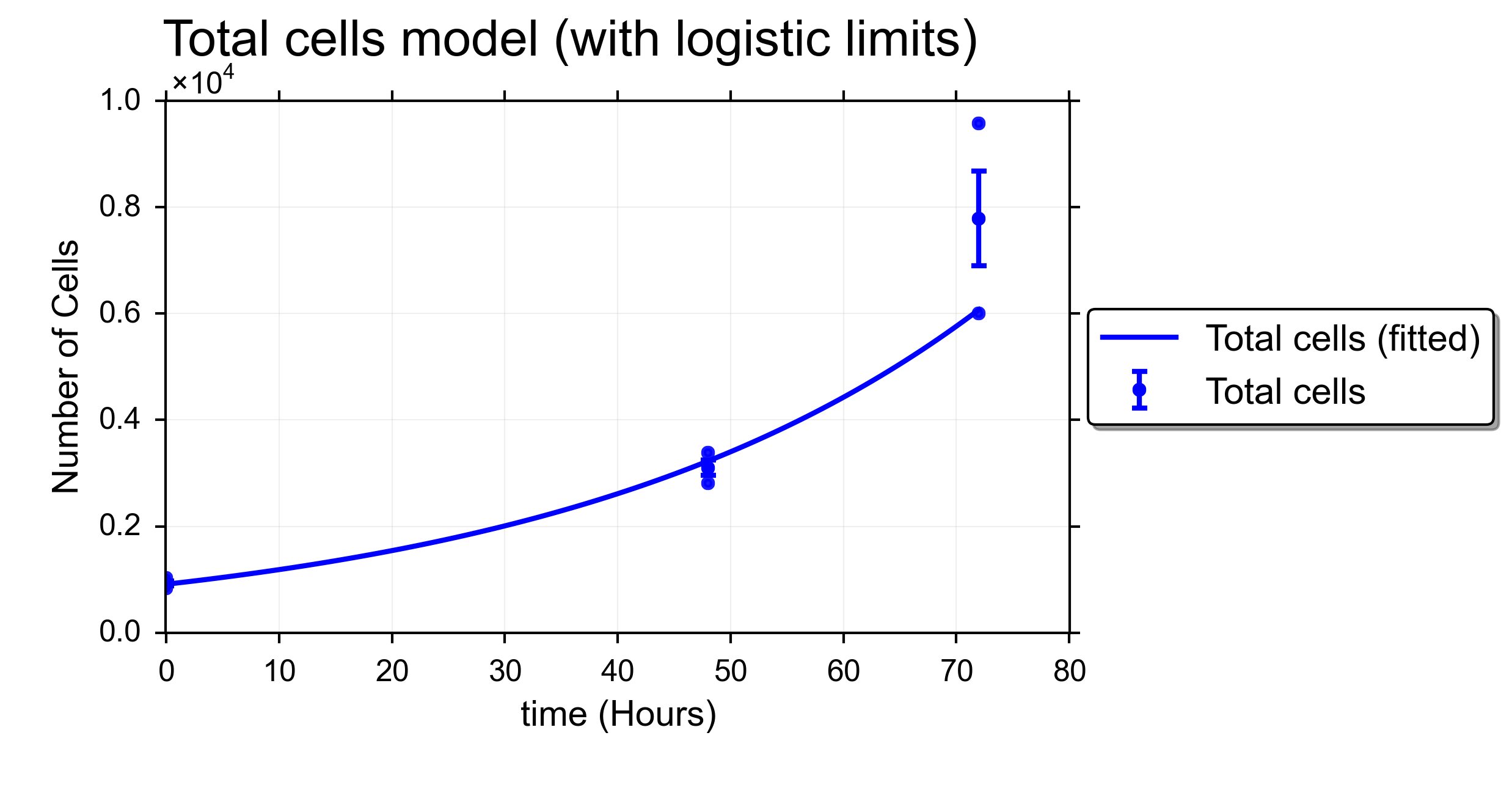

Supplement: Additional file 1: — Example of CellPD’s outputs. This folder contains two examples of the outputs generated by CellPD (using the data from Fig. 2 and Additional file 6). (ZIP 36462 kb) [file 12918_2016_337_MOESM1_ESM.zip › WFU/output/total_logistic/total_logistic.png]

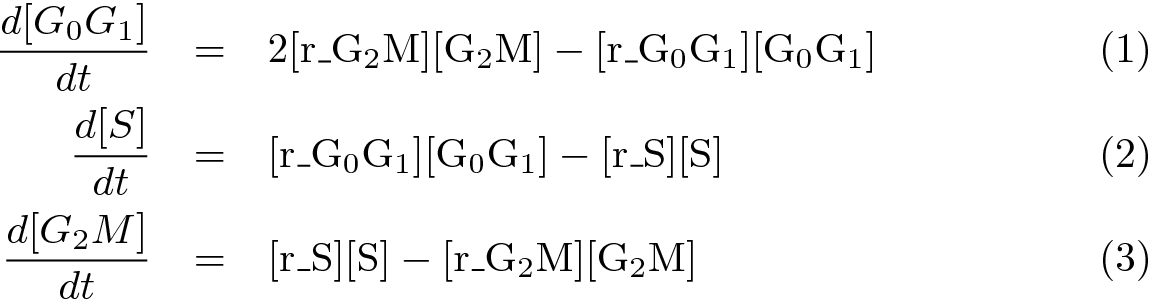

Supplement: Additional file 5: — Synthetic data. This folder contains the synthetic data used for Fig. 3 and Additional file 4. Additionally, it contains the python scripts that were used to make the synthetic data, Fig. 3, and Additional file 4: Figure S9-1. (ZIP 890 kb) [file 12918_2016_337_MOESM5_ESM.zip › synthetic_data/files/flow_cytometry/flow_cytometry_eqn.png]

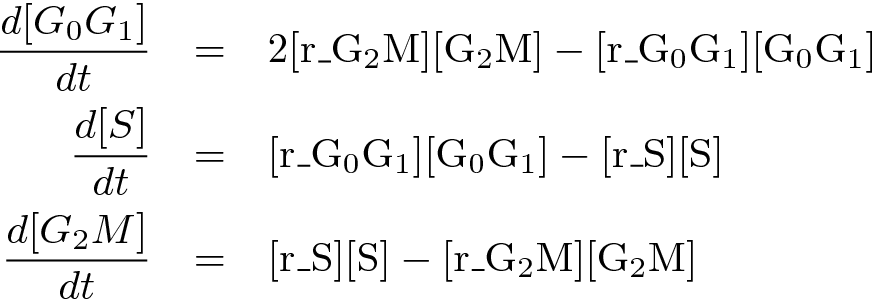

Supplement: Additional file 5: — Synthetic data. This folder contains the synthetic data used for Fig. 3 and Additional file 4. Additionally, it contains the python scripts that were used to make the synthetic data, Fig. 3, and Additional file 4: Figure S9-1. (ZIP 890 kb) [file 12918_2016_337_MOESM5_ESM.zip › synthetic_data/files/flow_cytometry/flow_cytometry_plane.png]

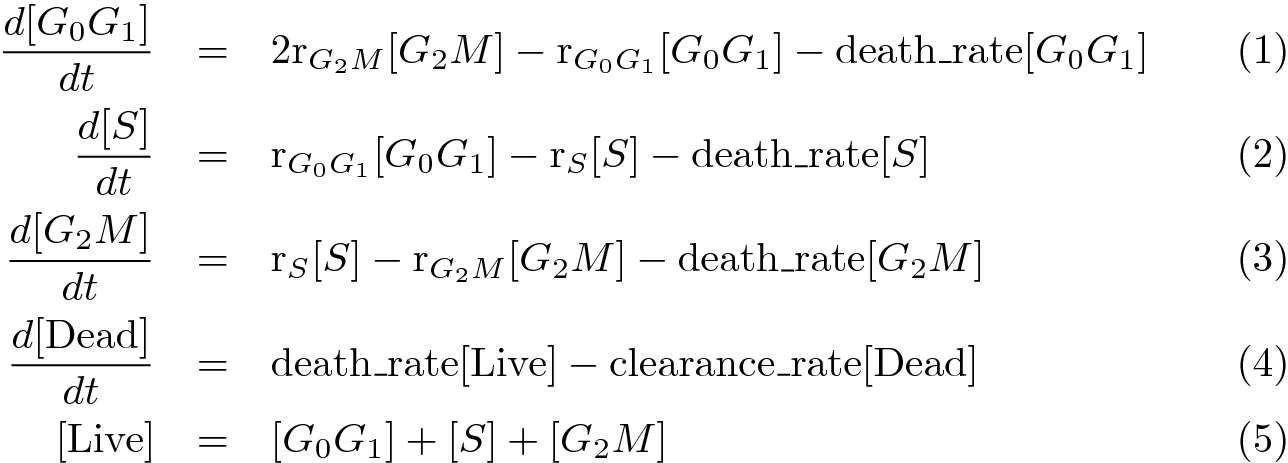

Supplement: Additional file 5: — Synthetic data. This folder contains the synthetic data used for Fig. 3 and Additional file 4. Additionally, it contains the python scripts that were used to make the synthetic data, Fig. 3, and Additional file 4: Figure S9-1. (ZIP 890 kb) [file 12918_2016_337_MOESM5_ESM.zip › synthetic_data/files/flow_cytometry_dead/flow_cytometry_dead.png]

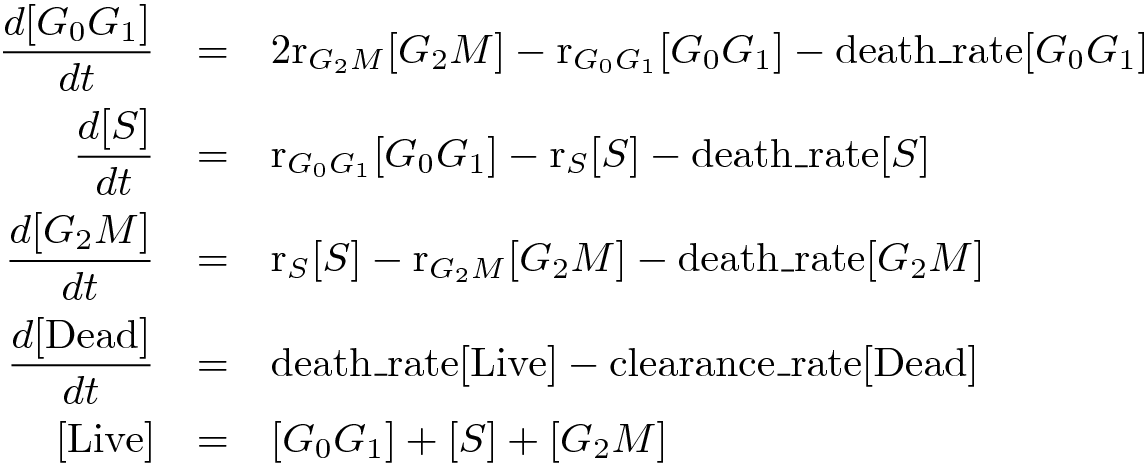

Supplement: Additional file 5: — Synthetic data. This folder contains the synthetic data used for Fig. 3 and Additional file 4. Additionally, it contains the python scripts that were used to make the synthetic data, Fig. 3, and Additional file 4: Figure S9-1. (ZIP 890 kb) [file 12918_2016_337_MOESM5_ESM.zip › synthetic_data/files/flow_cytometry_dead/flow_cytometry_dead_plane.png]

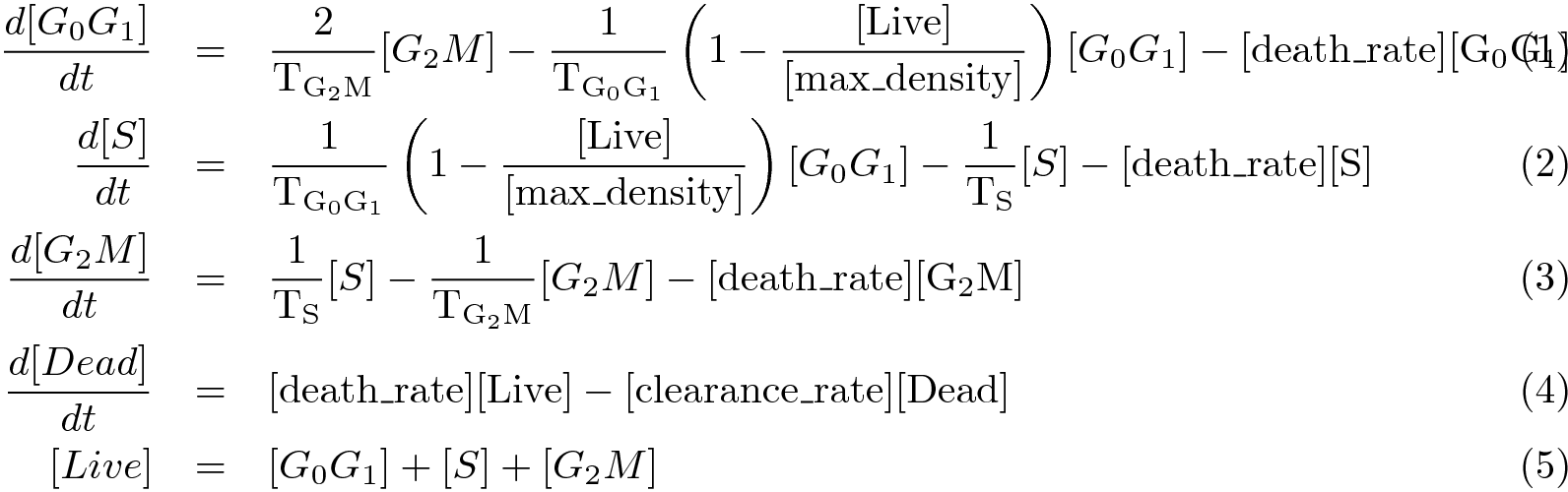

Supplement: Additional file 5: — Synthetic data. This folder contains the synthetic data used for Fig. 3 and Additional file 4. Additionally, it contains the python scripts that were used to make the synthetic data, Fig. 3, and Additional file 4: Figure S9-1. (ZIP 890 kb) [file 12918_2016_337_MOESM5_ESM.zip › synthetic_data/files/flow_cytometry_dead_logistic/flow_cytometry_dead_logistic_eqn.png]

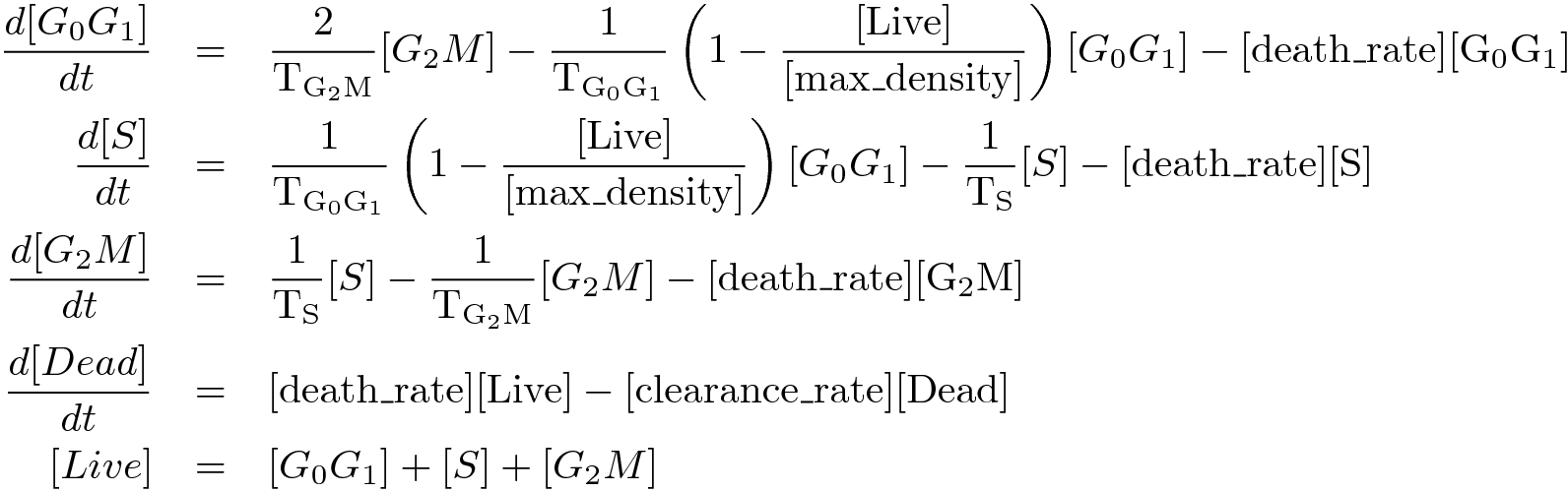

Supplement: Additional file 5: — Synthetic data. This folder contains the synthetic data used for Fig. 3 and Additional file 4. Additionally, it contains the python scripts that were used to make the synthetic data, Fig. 3, and Additional file 4: Figure S9-1. (ZIP 890 kb) [file 12918_2016_337_MOESM5_ESM.zip › synthetic_data/files/flow_cytometry_dead_logistic/flow_cytometry_dead_logistic_plane.png]

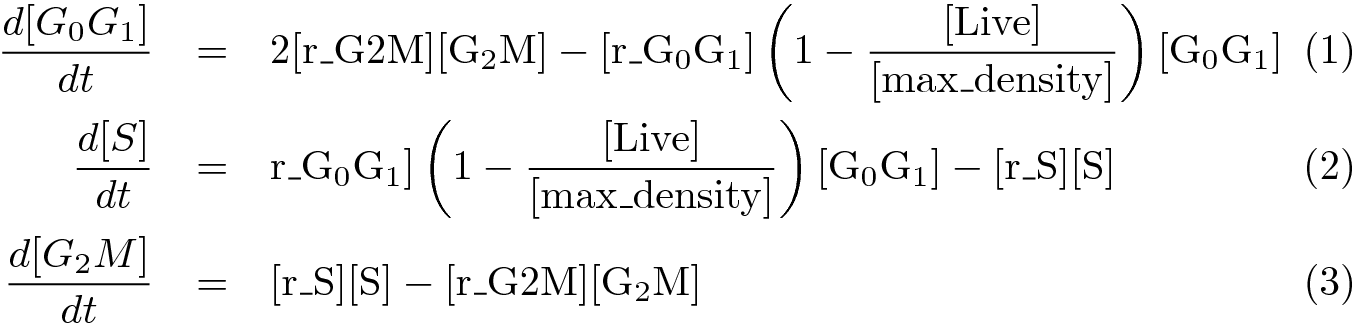

Supplement: Additional file 5: — Synthetic data. This folder contains the synthetic data used for Fig. 3 and Additional file 4. Additionally, it contains the python scripts that were used to make the synthetic data, Fig. 3, and Additional file 4: Figure S9-1. (ZIP 890 kb) [file 12918_2016_337_MOESM5_ESM.zip › synthetic_data/files/flow_cytometry_logistic/flow_cytometry_logistic_eqn.png]

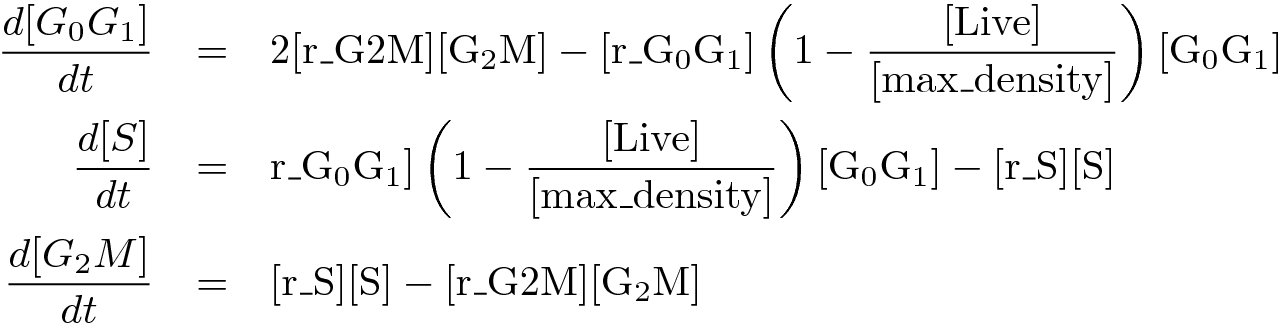

Supplement: Additional file 5: — Synthetic data. This folder contains the synthetic data used for Fig. 3 and Additional file 4. Additionally, it contains the python scripts that were used to make the synthetic data, Fig. 3, and Additional file 4: Figure S9-1. (ZIP 890 kb) [file 12918_2016_337_MOESM5_ESM.zip › synthetic_data/files/flow_cytometry_logistic/flow_cytometry_logistic_plane.png]

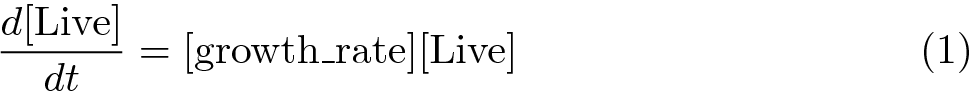

Supplement: Additional file 5: — Synthetic data. This folder contains the synthetic data used for Fig. 3 and Additional file 4. Additionally, it contains the python scripts that were used to make the synthetic data, Fig. 3, and Additional file 4: Figure S9-1. (ZIP 890 kb) [file 12918_2016_337_MOESM5_ESM.zip › synthetic_data/files/live/live_eqn.png]

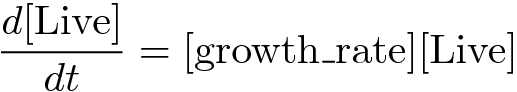

Supplement: Additional file 5: — Synthetic data. This folder contains the synthetic data used for Fig. 3 and Additional file 4. Additionally, it contains the python scripts that were used to make the synthetic data, Fig. 3, and Additional file 4: Figure S9-1. (ZIP 890 kb) [file 12918_2016_337_MOESM5_ESM.zip › synthetic_data/files/live/live_plane.png]

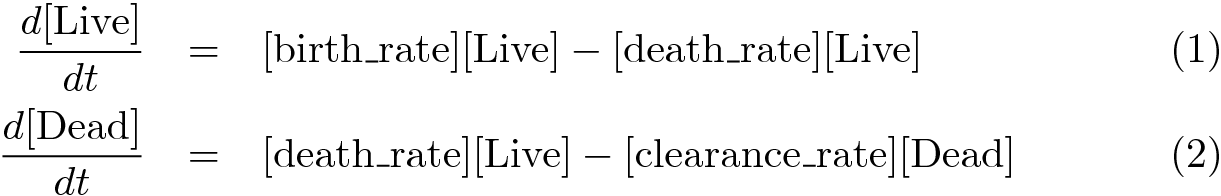

Supplement: Additional file 5: — Synthetic data. This folder contains the synthetic data used for Fig. 3 and Additional file 4. Additionally, it contains the python scripts that were used to make the synthetic data, Fig. 3, and Additional file 4: Figure S9-1. (ZIP 890 kb) [file 12918_2016_337_MOESM5_ESM.zip › synthetic_data/files/live_dead/live_dead_eqn.png]

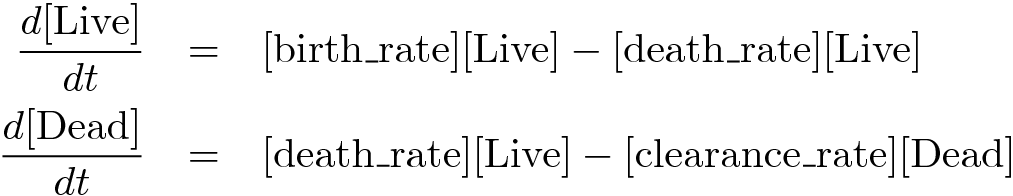

Supplement: Additional file 5: — Synthetic data. This folder contains the synthetic data used for Fig. 3 and Additional file 4. Additionally, it contains the python scripts that were used to make the synthetic data, Fig. 3, and Additional file 4: Figure S9-1. (ZIP 890 kb) [file 12918_2016_337_MOESM5_ESM.zip › synthetic_data/files/live_dead/live_dead_plane.png]

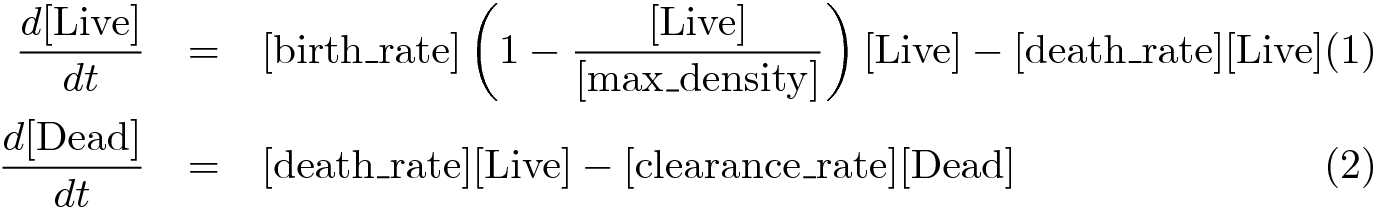

Supplement: Additional file 5: — Synthetic data. This folder contains the synthetic data used for Fig. 3 and Additional file 4. Additionally, it contains the python scripts that were used to make the synthetic data, Fig. 3, and Additional file 4: Figure S9-1. (ZIP 890 kb) [file 12918_2016_337_MOESM5_ESM.zip › synthetic_data/files/live_dead_logistic/live_dead_logistic_eqn.png]

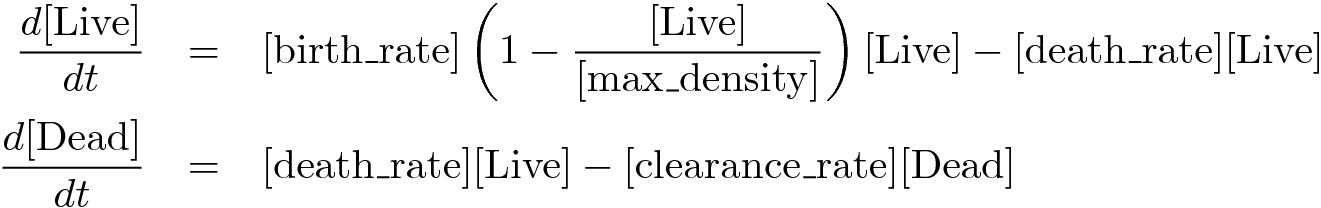

Supplement: Additional file 5: — Synthetic data. This folder contains the synthetic data used for Fig. 3 and Additional file 4. Additionally, it contains the python scripts that were used to make the synthetic data, Fig. 3, and Additional file 4: Figure S9-1. (ZIP 890 kb) [file 12918_2016_337_MOESM5_ESM.zip › synthetic_data/files/live_dead_logistic/live_dead_logistic_plane.png]

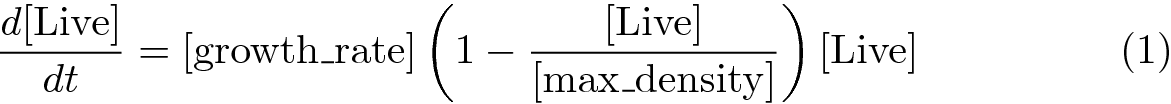

Supplement: Additional file 5: — Synthetic data. This folder contains the synthetic data used for Fig. 3 and Additional file 4. Additionally, it contains the python scripts that were used to make the synthetic data, Fig. 3, and Additional file 4: Figure S9-1. (ZIP 890 kb) [file 12918_2016_337_MOESM5_ESM.zip › synthetic_data/files/live_logistic/live_logistic_eqn.png]

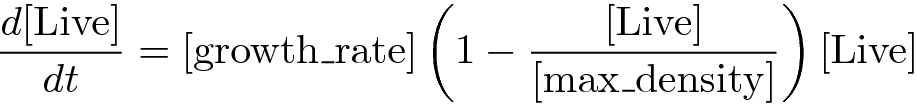

Supplement: Additional file 5: — Synthetic data. This folder contains the synthetic data used for Fig. 3 and Additional file 4. Additionally, it contains the python scripts that were used to make the synthetic data, Fig. 3, and Additional file 4: Figure S9-1. (ZIP 890 kb) [file 12918_2016_337_MOESM5_ESM.zip › synthetic_data/files/live_logistic/live_logistic_plane.png]

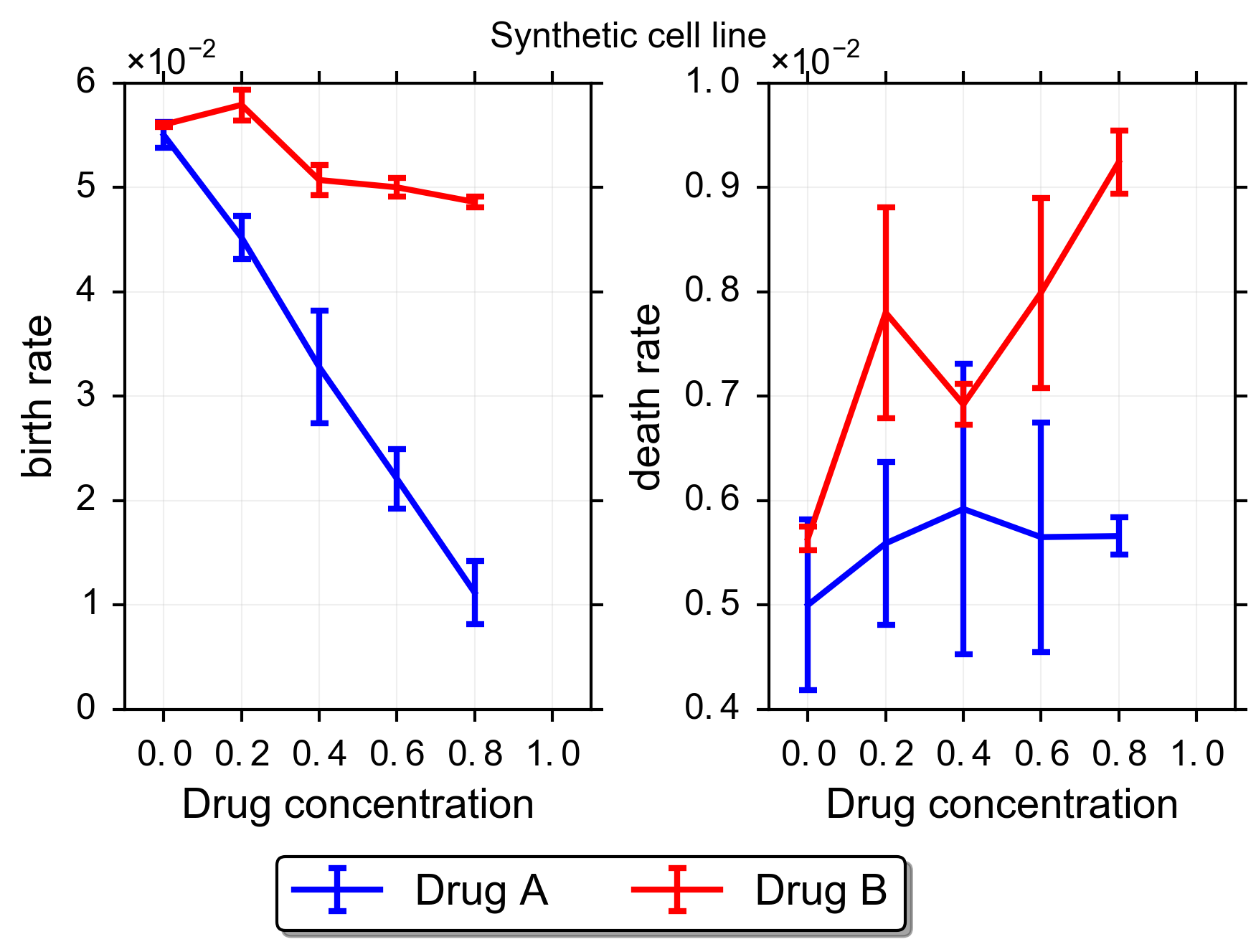

Supplement: Additional file 5: — Synthetic data. This folder contains the synthetic data used for Fig. 3 and Additional file 4. Additionally, it contains the python scripts that were used to make the synthetic data, Fig. 3, and Additional file 4: Figure S9-1. (ZIP 890 kb) [file 12918_2016_337_MOESM5_ESM.zip › synthetic_data/Synthetic_live_dead.png]

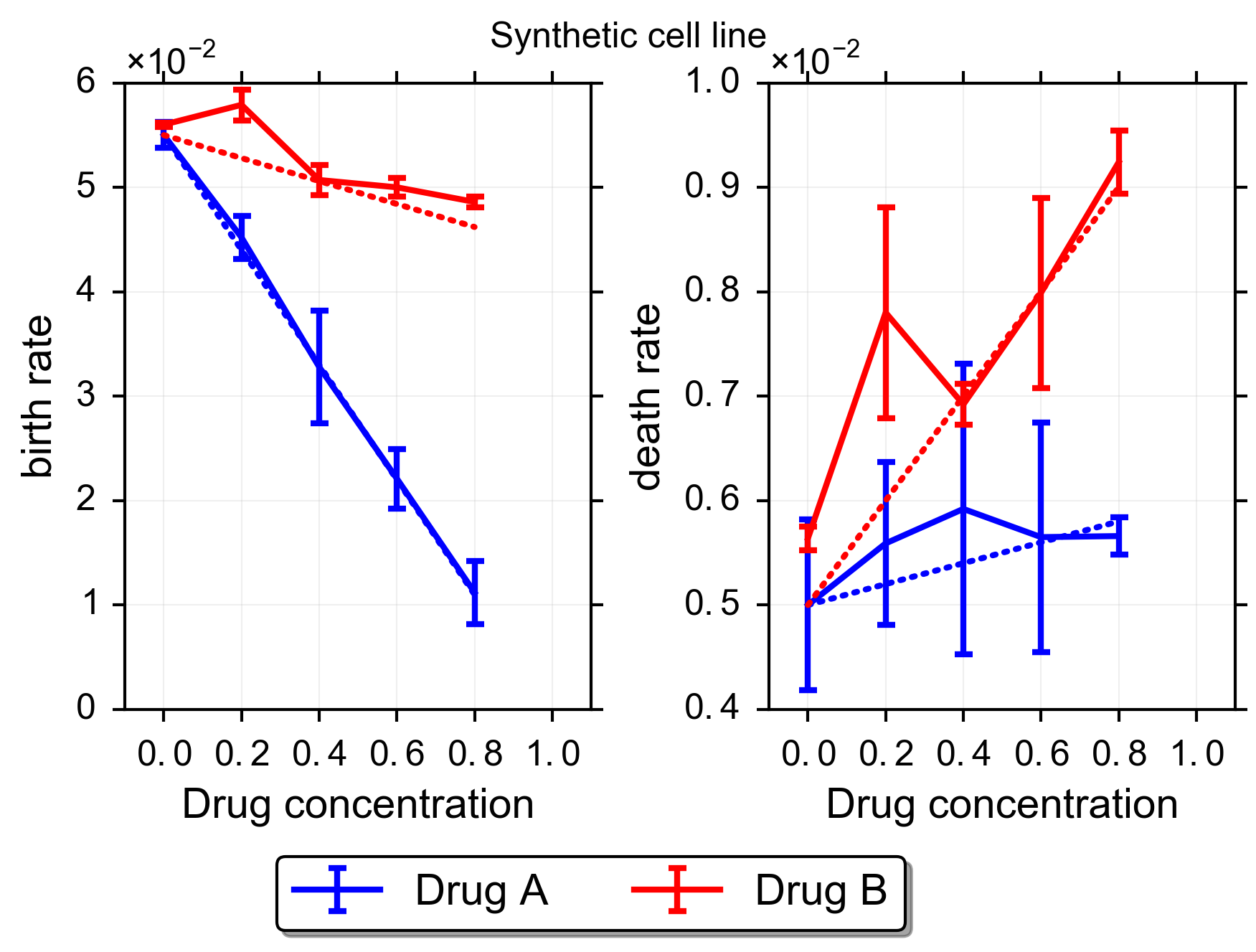

Supplement: Additional file 5: — Synthetic data. This folder contains the synthetic data used for Fig. 3 and Additional file 4. Additionally, it contains the python scripts that were used to make the synthetic data, Fig. 3, and Additional file 4: Figure S9-1. (ZIP 890 kb) [file 12918_2016_337_MOESM5_ESM.zip › synthetic_data/Synthetic_live_dead_supplementary.png]

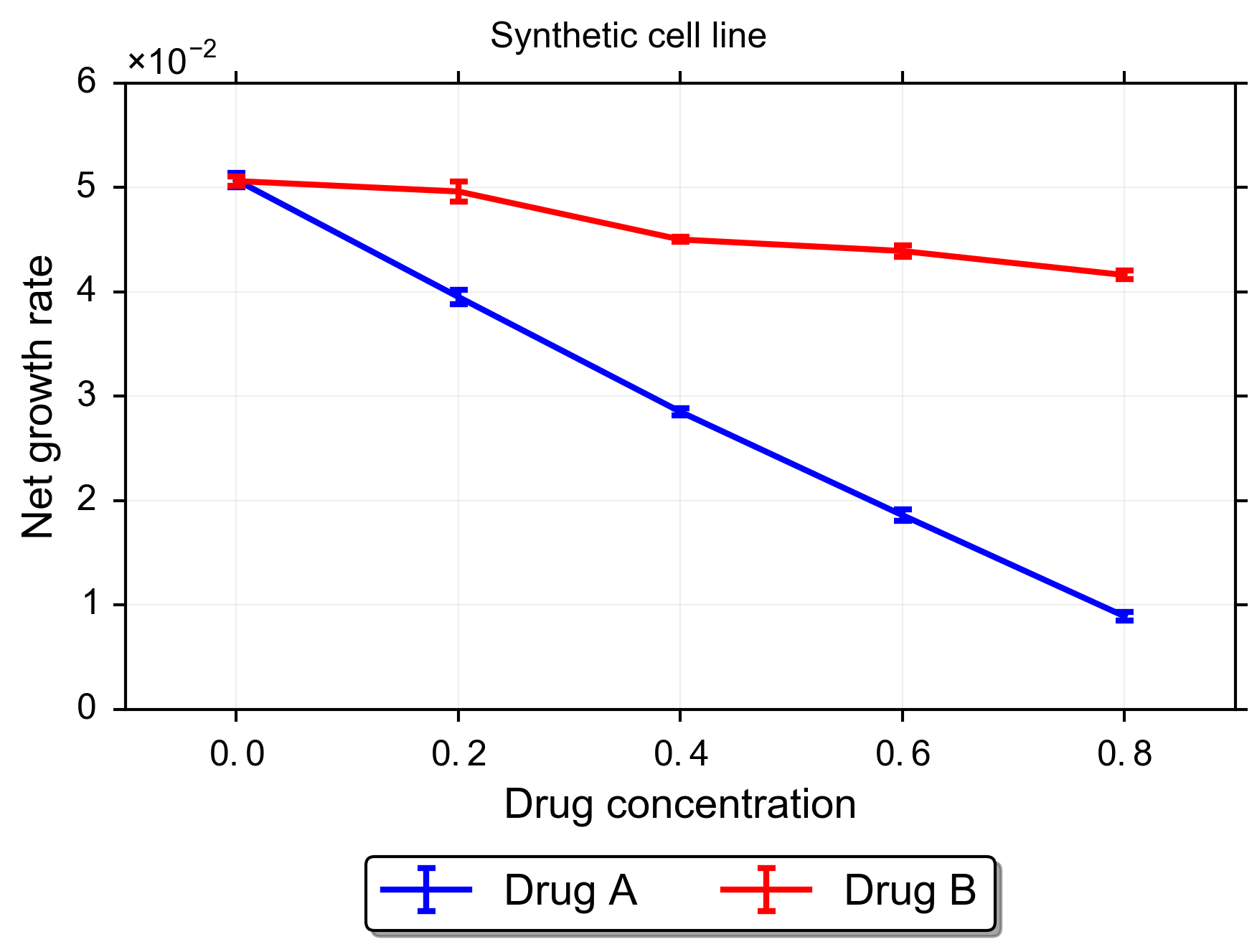

Supplement: Additional file 5: — Synthetic data. This folder contains the synthetic data used for Fig. 3 and Additional file 4. Additionally, it contains the python scripts that were used to make the synthetic data, Fig. 3, and Additional file 4: Figure S9-1. (ZIP 890 kb) [file 12918_2016_337_MOESM5_ESM.zip › synthetic_data/Synthetic_net_growth_rate.png]
