# Supplementary material for: Quantifying differences in cell line population dynamics using CellPD
Source: BMC Syst Biol. 2016 Sep 21;10:92. doi: 10.1186/s12918-016-0337-5 (PMC5031291; doi:10.1186/s12918-016-0337-5)
Supplement: Additional file 2: — Other tools that we examined. This file contains a list of five other tools we attempted to use and the reasons why we did not use them. (PDF 120 kb) [file 12918_2016_337_MOESM2_ESM.pdf]

Other tools that we examined

Additional file 2 for "*Quantifying Differences in Cell Line Population Dynamics Using CellPD*"

Authors: Edwin F. Juarez[1,2], Roy Lau[1], Samuel H. Friedman[1], Ahmadreza Ghaffarizadeh[1], Edmond Jonckheere[2], David B. Agus[1], Shannon M. Mumenthaler[1], and Paul Macklin[1].

Authors' affiliations:
1: Lawrence J. Ellison Institute for Transformative Medicine, University of Southern California.
2: Department of Electrical Engineering, Viterbi School of Engineering, University of Southern California.

Contact information: Paul Macklin: Paul.Macklin@usc.edu
Edwin F. Juarez: juarezro@usc.edu

grofit: An open source tool written in R. Performs parametric and nonparametric fits on data measured with a high content screening (HCS) microscope, but also with custom data. It works well with short sampling times, but it can also estimate growth rates with longer sampling times (but it becomes inaccurate with low number of samples). This tool was not used to validate CellPD because the parametric models it uses are modifications of their canonical forms [1].

GATHODE: Estimates growth rates on data measured with a HCS microscope. This tool was not used to validate CellPD because it only reads documents created by a HCS microscope [2].

GrowthRates: Estimates growth rates on data measured with a HCS microscope, for all 96 wells at the same time. This tool was not used to validate CellPD because it offers no flexibility on the format [3].

PHANTAST: An open source image processing toolset to, among other things, estimate growth and death rates [4]. This tool was not used to validate CellPD because it can only estimate growth rates when the data start as an image.

SBaddon: extension package to the Systems Biology Toolbox for MATLAB for parameter estimation [5]. This tool was not used to validate CellPD because it is designed for subcellular (i.e., single-cell) modeling rather than population-level modeling.

[1] M. Kahm, G. Hasenbrink, and F. Lichtenberg, "grofit: fitting biological growth curves with R," *Journal of Statistical Software,* vol. 33, pp. 1--21, 2010.
[2] P. P. Jung, N. Christian, D. P. Kay, A. Skupin, and C. L. Linster, "Protocols and programs for high-throughput growth and aging phenotyping in yeast," *PloS one,* vol. 10, p. e0119807, 2015.
[3] B. G. Hall, H. Acar, A. Nandipati, and M. Barlow, "Growth rates made easy," *Molecular biology and evolution,* vol. 31, pp. 232-238, 2014.
[4] N. Jaccard, L. D. Griffin, A. Keser, R. J. Macown, A. Super, F. S. Veraitch*, et al.*, "Automated method for the rapid and precise estimation of adherent cell culture characteristics from phase contrast microscopy images," *Biotechnology and bioengineering,* vol. 111, pp. 504--17, 2014.
[5] H. Schmidt and M. Jirstrand, "SBaddon: high performance simulation for the Systems Biology Toolbox for MATLAB," *Bioinformatics,* vol. 23, pp. 646--647, 2007.
