# Supplementary material for: Quantifying differences in cell line population dynamics using CellPD
Source: BMC Syst Biol. 2016 Sep 21;10:92. doi: 10.1186/s12918-016-0337-5 (PMC5031291; doi:10.1186/s12918-016-0337-5)
Supplement: Additional file 3: — Growth rate estimates versus sampling frequency. This file contains an alternative representation of the same data from Fig. 1. In this representation, the x-axis is the sampling interval (instead of the log 10 of the number of samples). (PDF 239 kb) [file 12918_2016_337_MOESM3_ESM.pdf]

Growth rate estimates versus sampling frequency

Additional file 3 for "*Quantifying Differences in Cell Line Population Dynamics Using CellPD*"

Authors: Edwin F. Juarez[1,2], Roy Lau[1], Samuel H. Friedman[1], Ahmadreza Ghaffarizadeh[1], Edmond Jonckheere[2], David B. Agus[1], Shannon M. Mumenthaler[1], and Paul Macklin[1].

Authors' affiliations:
1: Lawrence J. Ellison Institute for Transformative Medicine, University of Southern California.
2: Department of Electrical Engineering, Viterbi School of Engineering, University of Southern California.

Contact information: Paul Macklin: Paul.Macklin@usc.edu
Edwin F. Juarez: juarezro@usc.edu

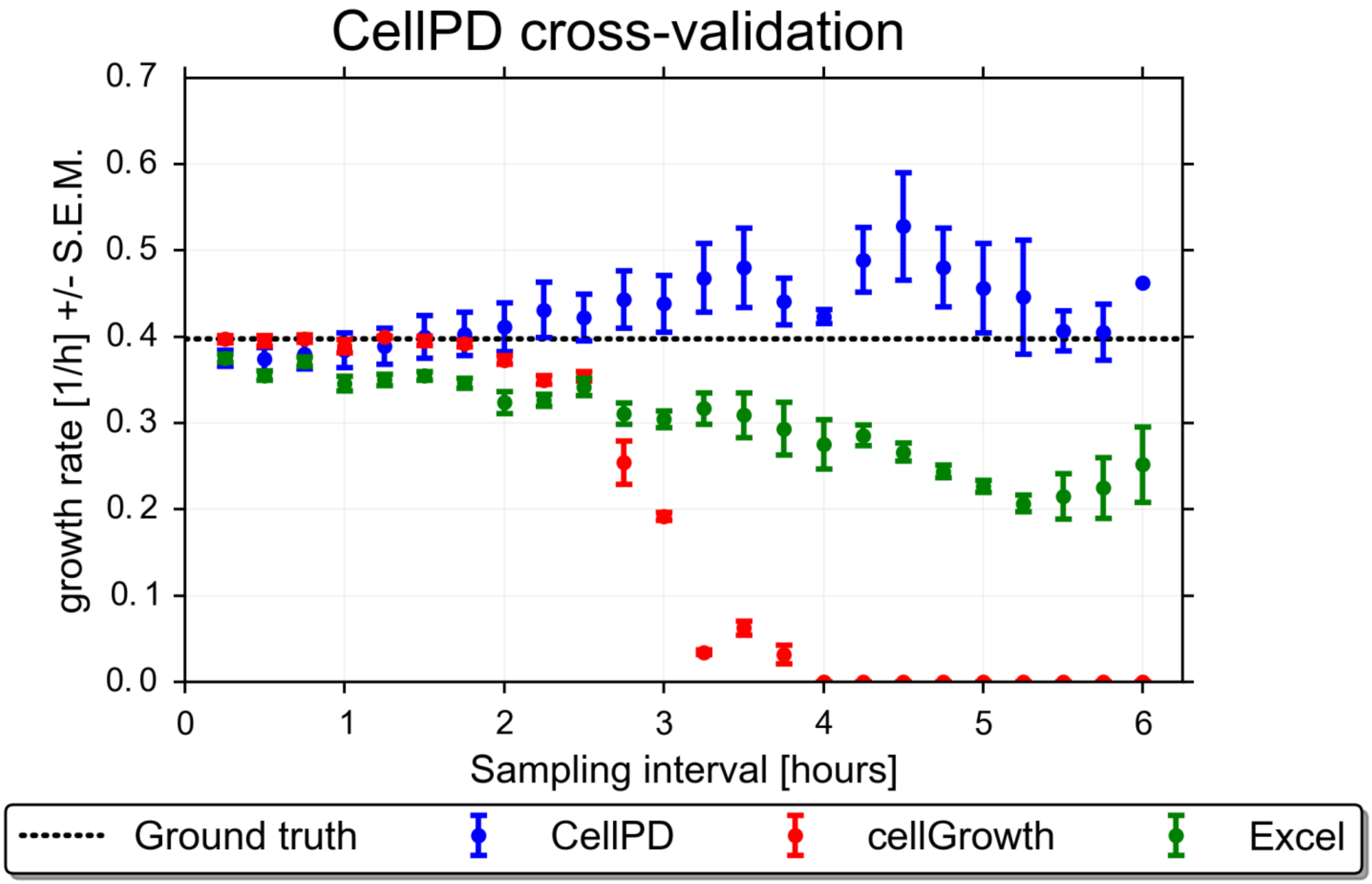

Figure S3-1: Growth rate estimates versus sampling frequency.

Growth rates ± Standard error of the mean (S.E.M) of Yeast strain seg_07A grown in YPD media computed by cellGrowth (red), Excel (green) and CellPD (blue) using different sampling intervals (which correspond to different number of sampling time points). All three tools correctly estimated the maximum growth rate for high sampling rates. For large sampling intervals (approximately 3 hours which corresponds to less than 10 samples), the three tools become less accurate; cellGrowth lacks the necessary number of data points to perform data smoothing, Excel becomes inaccurate, but CellPD continues to estimate reasonable growth rates. Even at the limit case of only 3 sampled data points (a sampling interval of 6 hours), CellPD provides a reasonable estimate (although it can no longer estimate S.E.M of the parameter).
