# Supplementary material for: Quantifying differences in cell line population dynamics using CellPD
Source: BMC Syst Biol. 2016 Sep 21;10:92. doi: 10.1186/s12918-016-0337-5 (PMC5031291; doi:10.1186/s12918-016-0337-5)
Supplement: Additional file 4: — Creation of synthetic data. This file contains a description of how the synthetic data for Fig. 3 was created, as well as the calculation of the estimation error. (PDF 500 kb) [file 12918_2016_337_MOESM4_ESM.pdf]

Creation of synthetic data

Additional file 3 for "*Quantifying Differences in Cell Line Population Dynamics Using CellPD*"

Authors: Edwin F. Juarez[1,2], Roy Lau[1], Samuel H. Friedman[1], Ahmadreza Ghaffarizadeh[1], Edmond Jonckheere[2], David B. Agus[1], Shannon M. Mumenthaler[1], and Paul Macklin[1].

Authors' affiliations:
1: Lawrence J. Ellison Institute for Transformative Medicine, University of Southern California.
2: Department of Electrical Engineering, Viterbi School of Engineering, University of Southern California.

Contact information: Paul Macklin: Paul.Macklin@usc.edu
Edwin F. Juarez: juarezro@usc.edu

In order to test the high-throughput capabilities of CellPD we generated a synthetic dataset. The goal of this dataset was to imitate the output file of a high content screening microscope. We simulated a (basic) drug screening experiment testing two compounds. We predefine the seeding density, birth rate, death rate, clearance rate, and sensitivity of a cell line for the mathematical model shown below:

$$\frac{dL}{dt} = birth\_rate(1 - L/L_{max})(1 - sensitivity\_1 \times cytostaticity \times concentration)L - death\_rate(1 + sensitivity\_2 \times citotoxicity \times concentration)L$$

$$\frac{dD}{dt} = death\_rate(1 + sensitivity_2 \times citotoxicity \times concentration)L - clearance\_rate \times D$$

$$L(0) = seeding\_cells\_live$$

$$D(0) = seeding\_cells\_dead$$

birth_rate = 5.5e-2 h^{-1}
death_rate = 5.0e-3 h^{-1}
clearance_rate = 2.0e-3 h^{-1}
carrying_capacity = 1.0e9 cells
seeding_cells_live = 1.0e3 cells
seeding_cells_dead = 1.0e1 cells
sensitivity_1 = 1.0
sensitivity_2 = 1.0
concentration = 0, 0.2, 0.4, 0.6, 0.8

To simulate a cytostatic drug, we set $cytostaticity = 1.0$ and $citotoxicity = 0.20$, similarly, to simulate a cytotoxic drug, we set $cytostaticity = 0.20$ and $citotoxicity = 1.0$. We simulate three biological replicates for each drug concentration. In order to simulate biological heterogeneity and measurement error, we add a Gaussian noise of 15% to each biological replicate.

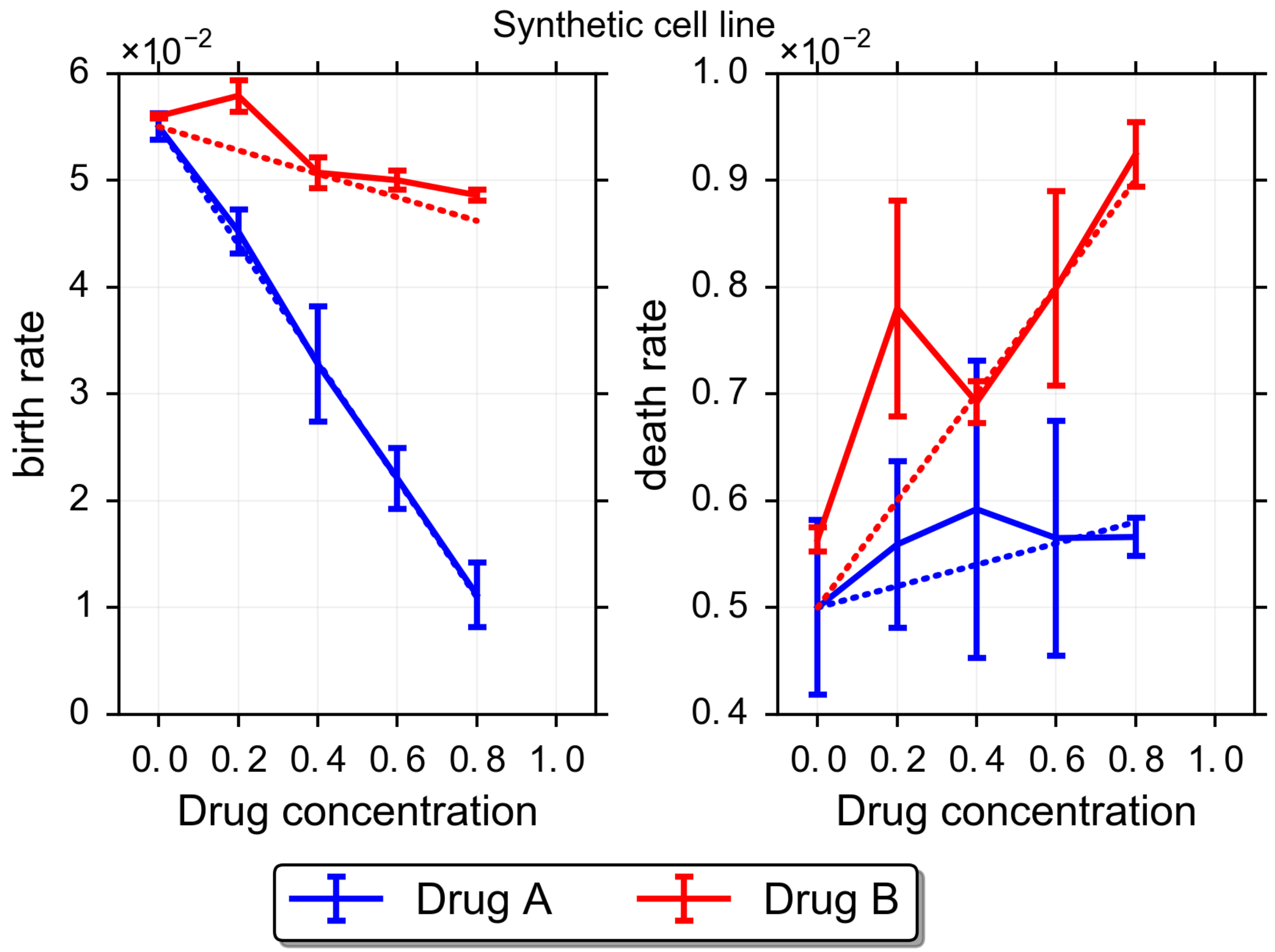

Figure S9-1: CellPD's parameters estimates compared with known true parameters. CellPD correctly estimates the birth rate (left) of a synthetic cell line exposed to two different synthetic drugs and elucidates the general trend of the death rate (right) for both drugs as well. See tables S9-1, S9-2, and S9-3 for the calculation of estimation error.

Table S9-1: Birth rate estimate error					
Drug Name	Concentration	True birth rate	Estimate	Difference	Percent Error
Drug A	0	0.055	0.055	0	0
Drug A	0.2	0.044	0.0452	0.0012	2.727
Drug A	0.4	0.033	0.0328	0.0002	0.606
Drug A	0.6	0.022	0.0221	0.0001	0.455
Drug A	0.8	0.011	0.0112	0.0002	1.818
Drug B	0	0.055	0.056	0.001	1.818
Drug B	0.2	0.0528	0.0579	0.0051	9.659
Drug B	0.4	0.0506	0.0507	0.0001	0.197
Drug B	0.6	0.0484	0.05	0.0016	3.305
Drug B	0.8	0.0462	0.0486	0.0024	5.195
				Mean percent error:	2.578

Table S9-2: Death rate estimate error					
Drug Name	Concentration	True death rate	Estimate	Difference	Percent Error
Drug A	0	0.005	0.005	0	0
Drug A	0.2	0.0052	0.00559	0.00039	7.5
Drug A	0.4	0.0054	0.00592	0.00052	9.630
Drug A	0.6	0.0056	0.00565	5.00E-05	0.893
Drug A	0.8	0.0058	0.00566	0.00014	2.414
Drug B	0	0.005	0.00564	0.00064	12.8
Drug B	0.2	0.006	0.0078	0.0018	30
Drug B	0.4	0.007	0.00692	8.00E-05	1.143
Drug B	0.6	0.008	0.00799	1.00E-05	0.125
Drug B	0.8	0.009	0.00924	0.00024	2.667
				Mean percent error:	6.717

Table S9-3: Death rate estimate error					
Drug Name	Concentration	True net growth rate	Estimate	Difference	Percent Error
Drug A	0	0.05	0.05	0	0
Drug A	0.2	0.0388	0.03961	0.00081	2.088
Drug A	0.4	0.0276	0.02688	0.00072	2.609
Drug A	0.6	0.0164	0.01645	5.00E-05	0.305
Drug A	0.8	0.0052	0.00554	0.00034	6.538
Drug B	0	0.05	0.05036	0.00036	0.72
Drug B	0.2	0.0468	0.0501	0.0033	7.051
Drug B	0.4	0.0436	0.04378	0.00018	0.413
Drug B	0.6	0.0404	0.04201	0.00161	3.985
Drug B	0.8	0.0372	0.03936	0.00216	5.806
				Mean percent error:	2.952
