# Supplementary material for: Quantifying differences in cell line population dynamics using CellPD
Source: BMC Syst Biol. 2016 Sep 21;10:92. doi: 10.1186/s12918-016-0337-5 (PMC5031291; doi:10.1186/s12918-016-0337-5)
Supplement: Additional file 5: — Synthetic data. This folder contains the synthetic data used for Fig. 3 and Additional file 4. Additionally, it contains the python scripts that were used to make the synthetic data, Fig. 3, and Additional file 4: Figure S9-1. (ZIP 890 kb) [file 12918_2016_337_MOESM5_ESM.zip › synthetic_data/files/index_template.html]

{0[cell\_line\_name]:s}: CellPD Results


# CellPD Results

## {0[cell\_line\_name]:s} cell line

These are the main results from running the {0[tool\_name]:s} (Version {0[version]:1.1f}). Please cite this tool as:

> {0[citation\_text]:s}

**[Download Digital Cell Line]**

**[Download these reports as a standalone zip file]**

## User-supplied data

Here are the data, as given by {0[user\_name]:s} on {0[date]:s}:

{0[data\_table]:s}

**Fig. 1:** {0[data\_caption]:s}  
[Download as PNG]
[Download as SVG]  
[Download as PNG (black & white)]
[Download as SVG (black & white)]

### Metadata

These data were provided by:

{0[user\_data\_table]:s}

Cell line information:

{0[experiment\_data\_table]:s}

---

## Models at a glance

Here is a summary of the model fitting results. Click any model for its fitted parameters and plots.

Ranked by Mean Absolute Percentage Error (MAPE)

[Lower MAPE is better.]

Warning legend: c = high correlation, u = high uncertainty, e = error estimates missing

{0[models\_table]:s}

Ranked by Reduced Chi Squared Goodness of Fit (Χ2ν)

[Lower Χ2ν is better.]

Warning legend: c = high correlation, u = high uncertainty, e = error estimates missing

{0[gof\_table]:s}

**Fig. 1:** {0[data\_caption]:s}  
[Download as PNG]
[Download as SVG]

---

[Back to top]

{0[tool\_name]:s} (Version {0[version]:1.1f}) - Results

[Back to the main page]
