# Supplementary material for: Quantifying differences in cell line population dynamics using CellPD
Source: BMC Syst Biol. 2016 Sep 21;10:92. doi: 10.1186/s12918-016-0337-5 (PMC5031291; doi:10.1186/s12918-016-0337-5)
Supplement: Additional file 5: — Synthetic data. This folder contains the synthetic data used for Fig. 3 and Additional file 4. Additionally, it contains the python scripts that were used to make the synthetic data, Fig. 3, and Additional file 4: Figure S9-1. (ZIP 890 kb) [file 12918_2016_337_MOESM5_ESM.zip › synthetic_data/files/report_template.html]

{0[tool\_name]:s} Report


# Fitting results: {0[model\_name]:s}

## MultiCellDS model: {0[MCDS\_name]:s}

## {0[cell\_line\_name]:s} cell line

These are the results from running the {0[tool\_name]:s} (Version {0[version]:1.1f}) for the {0[model\_name]:s}. Please cite this tool as

> {0[citation\_text]:s}

---

## Model used

## Model description

{0[model\_description]:s}

---

## Fitted Parameters

{0[parameters\_table]:s}

### Derived parameters

Corrected exponential doubling time: {0[exponential\_doubling\_time]:1.3g} hours

Rough doubling time [ignoring logistic limitations and post-thaw-lags]: {0[naive\_doubling\_time]:1.3g} hours


**Table 1** {0[table\_caption]:s}

## Model fit

**Fig. 1:** {0[model\_caption]:s}  
[Download as PNG]
[Download as SVG]
[Download as PNG (black & white)]
[Download as SVG (black & white)]

---

[Back to top]

{0[tool\_name]:s} (Version {0[version]:1.1f}) - Results

[Back to the main page]
