# Supplementary material for: Quantifying differences in cell line population dynamics using CellPD
Source: BMC Syst Biol. 2016 Sep 21;10:92. doi: 10.1186/s12918-016-0337-5 (PMC5031291; doi:10.1186/s12918-016-0337-5)
Supplement: Additional file 7: — CellPD Tutorial. This file contains a step by step tutorial detailing how to add experimental data and metadata to CellPD’s input file, as well as how to run CellPD in Windows, OSX, or Linux. (PDF 2240 kb) [file 12918_2016_337_MOESM7_ESM.pdf]

CellPD Tutorial

Supplementary file 1 for
Quantifying Differences in Cell Line Population Dynamics Using CellPD

Authors: Edwin F. Juarez[1,2], Roy Lau[1], Samuel H. Friedman[1], Ahmadreza Ghaffarizadeh[1], Edmond Jonckheere[2], David B. Agus[1], Shannon M. Mumenthaler[1], and Paul Macklin[1].

Authors' affiliations:
1: USC Center for Applied Molecular Medicine, Keck School of Medicine, University of Southern California.
2: Department of Electrical Engineering, Viterbi School of Engineering, University of Southern California.

Contact information: Paul Macklin: Paul.Macklin@usc.edu
Edwin F. Juarez: juarezro@usc.edu

0- What you will need for this tutorial

All of the necessary files for this tutorial are included in `CellPD_tutorial.zip`, which can be downloaded from CellPD.sf.net. Extract the zip file and open the folder that you just uncompressed. Open the folder corresponding to the operating system that you are using.

For Windows make sure the following folders and files are present:

- CellPD
- files
- CellPD.py
- input_s1.xlsx
- runCellPD.bat
- tutorial.docx

For OSX make sure the following folders and files are present:

- files
- venv
- CellPD.py
- CellPD27.py
- Input_s1.xlsx
- runCellPD
- tutorial.docx

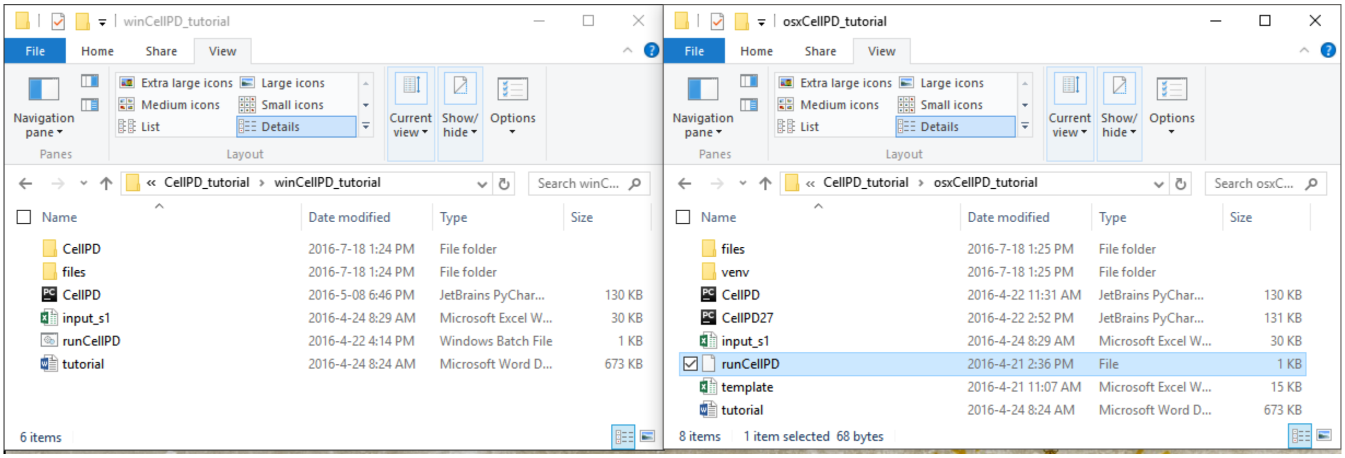

Figure 1: List of folders and files compressed in CellPD_tutorial.zip. On the left, the folder corresponding to the Windows tutorial. On the right, the folder corresponding to the OSX tutorial.

1- From raw data to CellPD input

Let us start with a typical example of data gathered in a laboratory. In this example we will use the data used in the main paper and we will simulate the typical usage for CellPD. Figure S2 shows a typical excel file which gathers the data from a yeast growth experiment under one experimental condition (cells grown in a glucose rich media, “YPD”). These data can be found in Gagneur et al., 2013. Note that this data set contains 6 biological replicate and 1 technical replicate for each biological replicate. CellPD can also handle different number of technical and/or biological replicates.

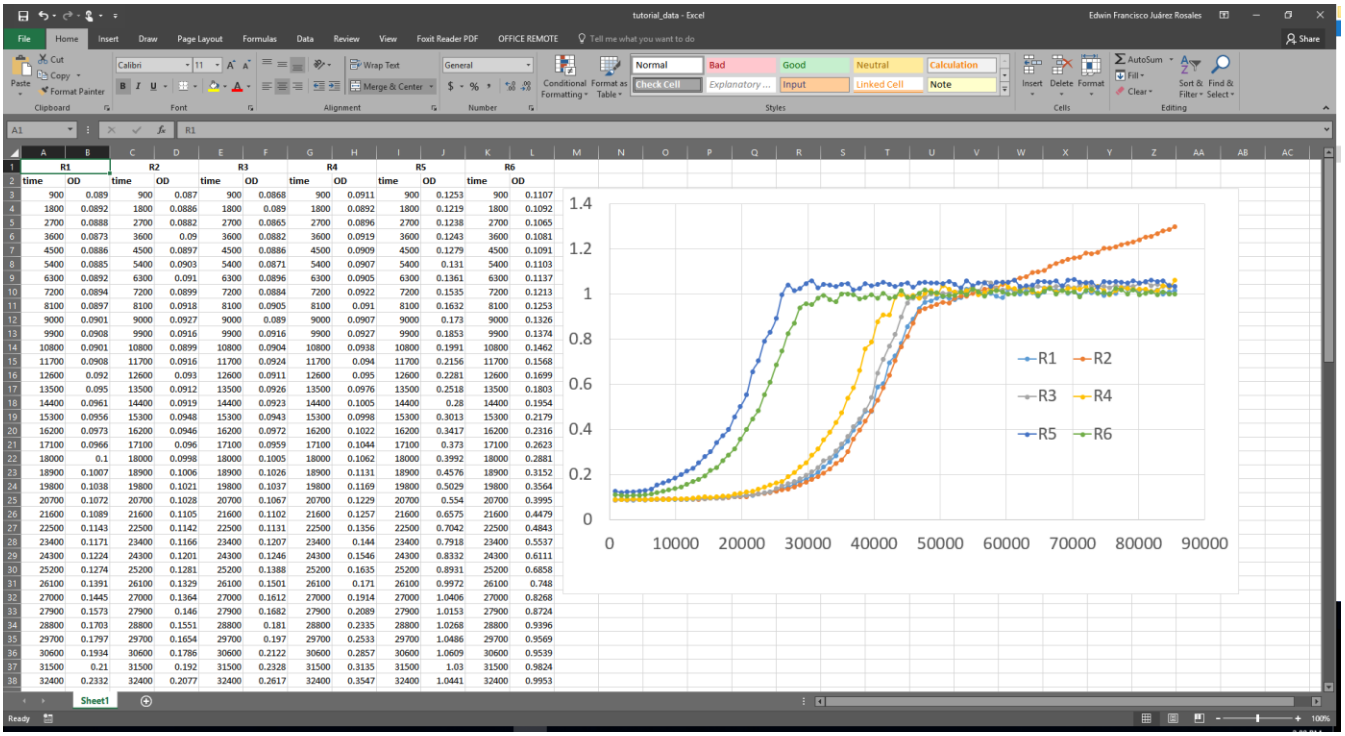

Figure 2: Example of raw data collected in an excel file. Data provided as `tutorial_data.xlsx.`

Next, we copy those data into the CellPD input file.

Open the file input_s1.xlsx and navigate straight to the tab called `Total_Cells`. Note that for this example we provide a file template which already includes all the data, so when you run your own experiments you may erase the data from these files or use one of the clean templates: `_replicates_template.xlsx` (for data which is arranged in biological and technical replicates) or `_means_template.xlsx` (for data which is summarized as means, standard deviations, and standard errors), available for downloading at CellPD.sf.net.

Note that if this example had included cell counts and viability data, then the correct tab to use is `Total+Viability`. Also note the time units. When we configure the `Preferences` we will need to specify these units. In this example we are using Seconds as the units of measurement, the label on this column does not have any effect, therefor we need to select the correct units in the `Preferences` tab

In this example we labeled each biological replicate with cardinal numbers. CellPD also recognizes numbers or letters as ID's for replicates. If two rows under the "Biological Replicate ID" column have the same ID, then CellPD will assume them to be different technical replicates of the same biological replicate, but if those two rows have the same ID under both Biological Replicate ID and Technical Replicate ID column, then it will recognize this as an error.

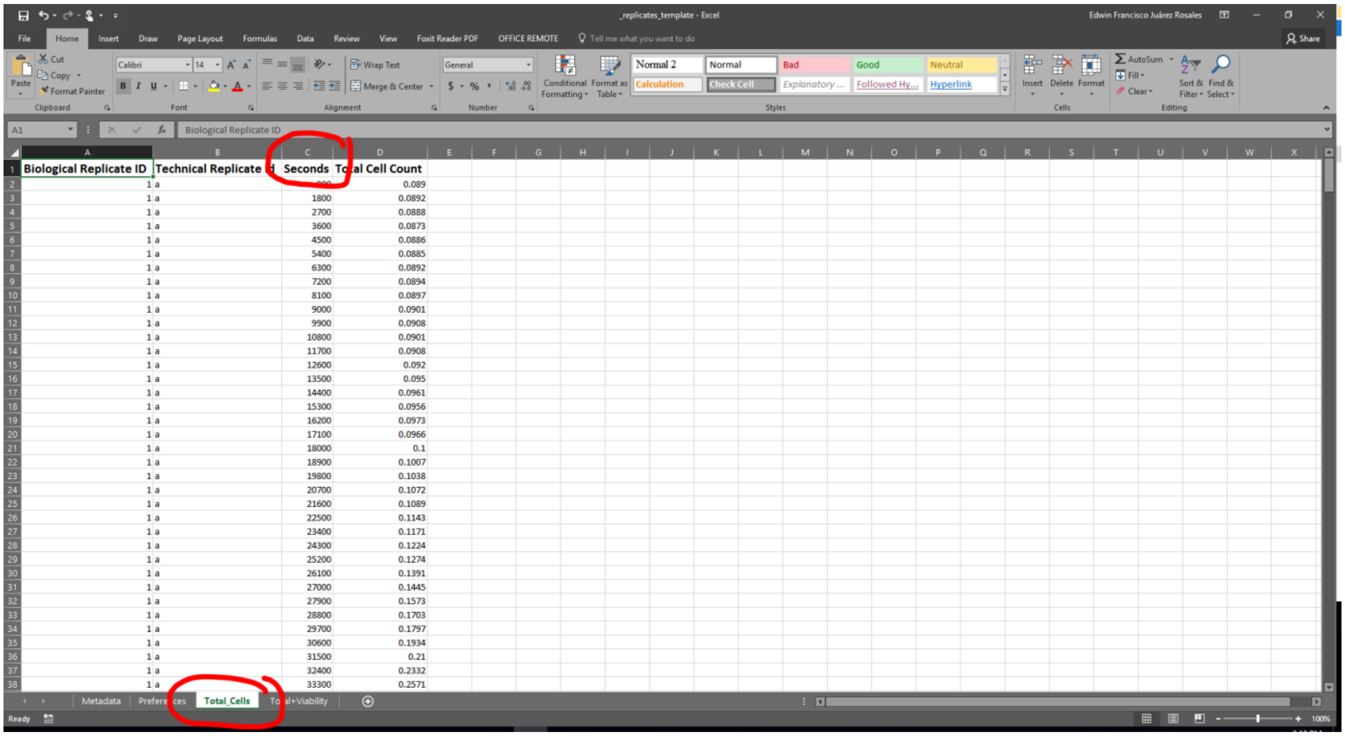

Biological Replicate ID	Technical Replicate ID	Seconds	Total Cell Count
1	a	[illegible]	0.089
1	a	1800	0.0892
1	a	2700	0.0888
1	a	3600	0.0873
1	a	4500	0.0886
1	a	5400	0.0885
1	a	6300	0.0892
1	a	7200	0.0894
1	a	8100	0.0897
1	a	9000	0.0901
1	a	9900	0.0908
1	a	10800	0.0901
1	a	11700	0.0908
1	a	12600	0.092
1	a	13500	0.095
1	a	14400	0.0961
1	a	15300	0.0956
1	a	16200	0.0973
1	a	17100	0.0966
1	a	18000	0.1
1	a	18900	0.1007
1	a	19800	0.1038
1	a	20700	0.1072
1	a	21600	0.1089
1	a	22500	0.1143
1	a	23400	0.1171
1	a	24300	0.1224
1	a	25200	0.1274
1	a	26100	0.1391
1	a	27000	0.1445
1	a	27900	0.1573
1	a	28800	0.1703
1	a	29700	0.1797
1	a	30600	0.1934
1	a	31500	0.21
1	a	32400	0.2332
1	a	33300	0.2571

Figure 2: Experimental data collected in a Microsoft Excel file as input for CellPD.

Next we configure the Preferences.

Open the tab called `Preferences`.

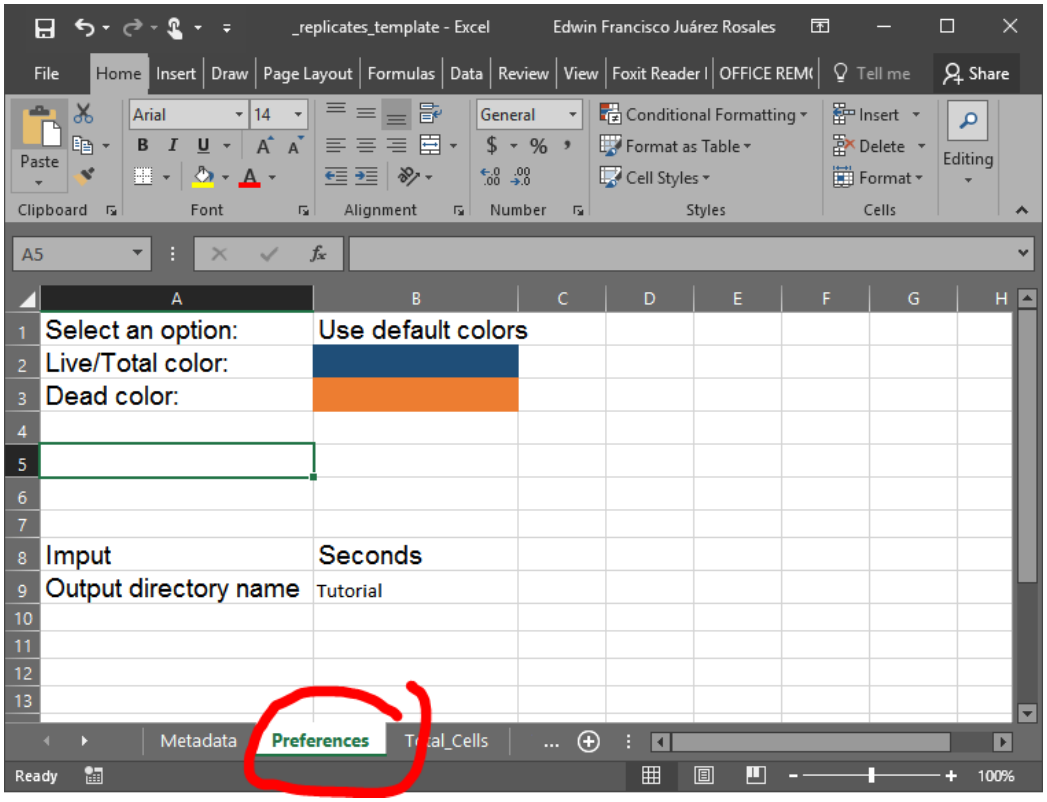

Figure 3: The `Preferences` *tab.*

Choose from the drop-down menu of cell B1 one of the three options: Use custom HEX colors, use cell fill color, use default colors. In this example we are using the default colors, but note that if we wanted to define our own HEX values we need to include the pound (#) character. For some online tools to pick HEX codes check http://colorbrewer2.org/ and http://www.w3schools.com/colors/colors_picker.asp.

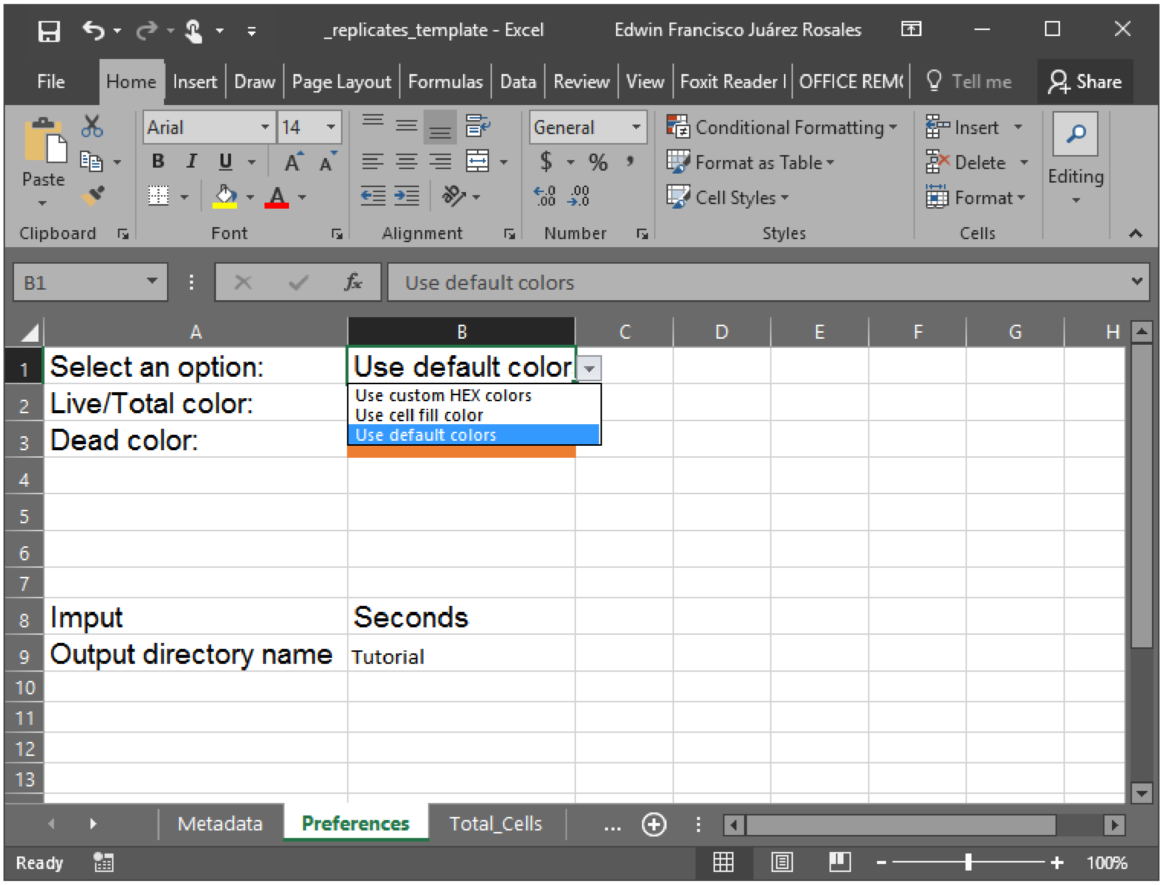

Figure4: Drop down menu with the options to choose color input type.

We also choose the input timescale as Seconds,

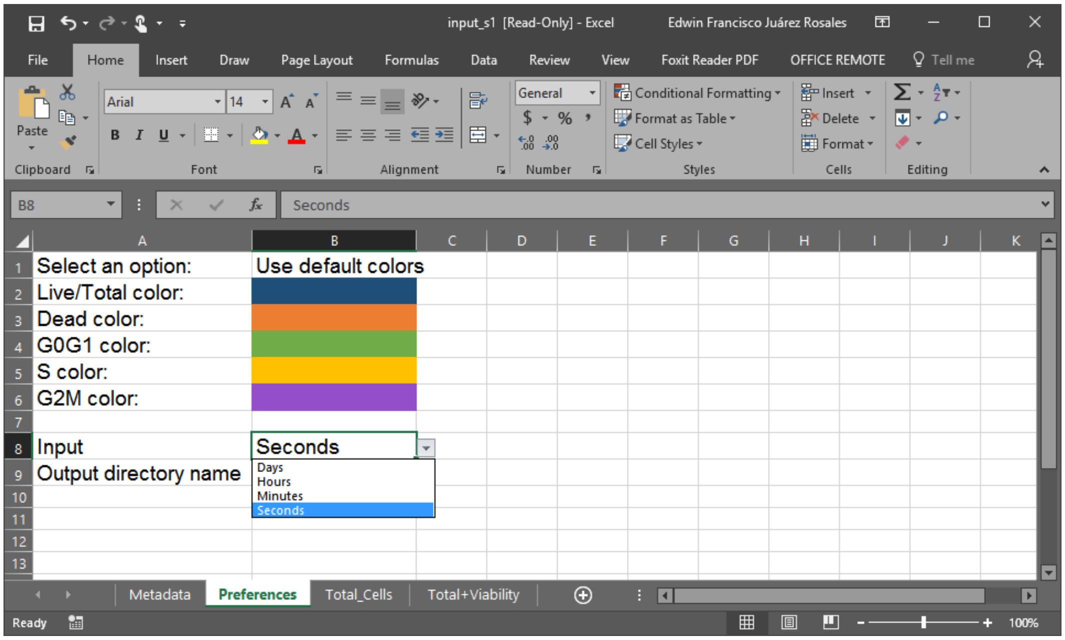

Figure 5: Drop down menu showing the timescale input options.

And we choose the output directory name to be Tutorial (CellPD will create a directory with this name if it does not exist yet).

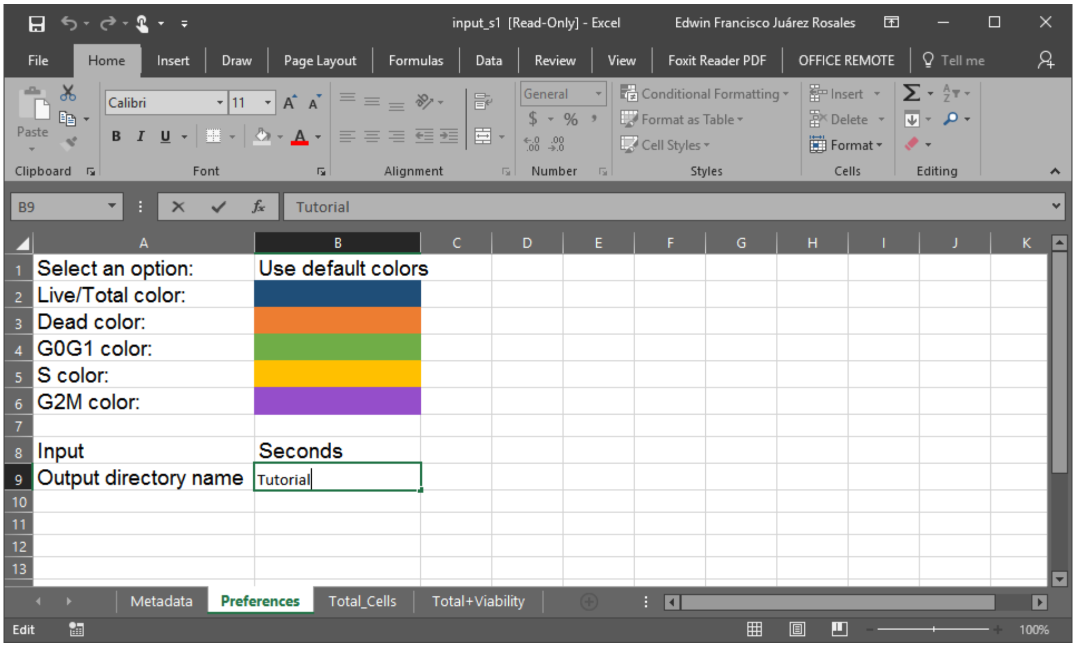

Figure 6: Choosing a name for the output directory.

Finally, we configure the `Metadata` tab. This part of the input file is crucial, most of the information written in this tab will *not* affect the computations performed by CellPD but it will allow CellPD to achieve the important task of aggregating information which can aid in data reproducibility. This tab requires careful input of information the first time the user is working with a new cell line (for that reason, templates for all of the cell lines distributed by ATCC are available at MultiCellDS.org).

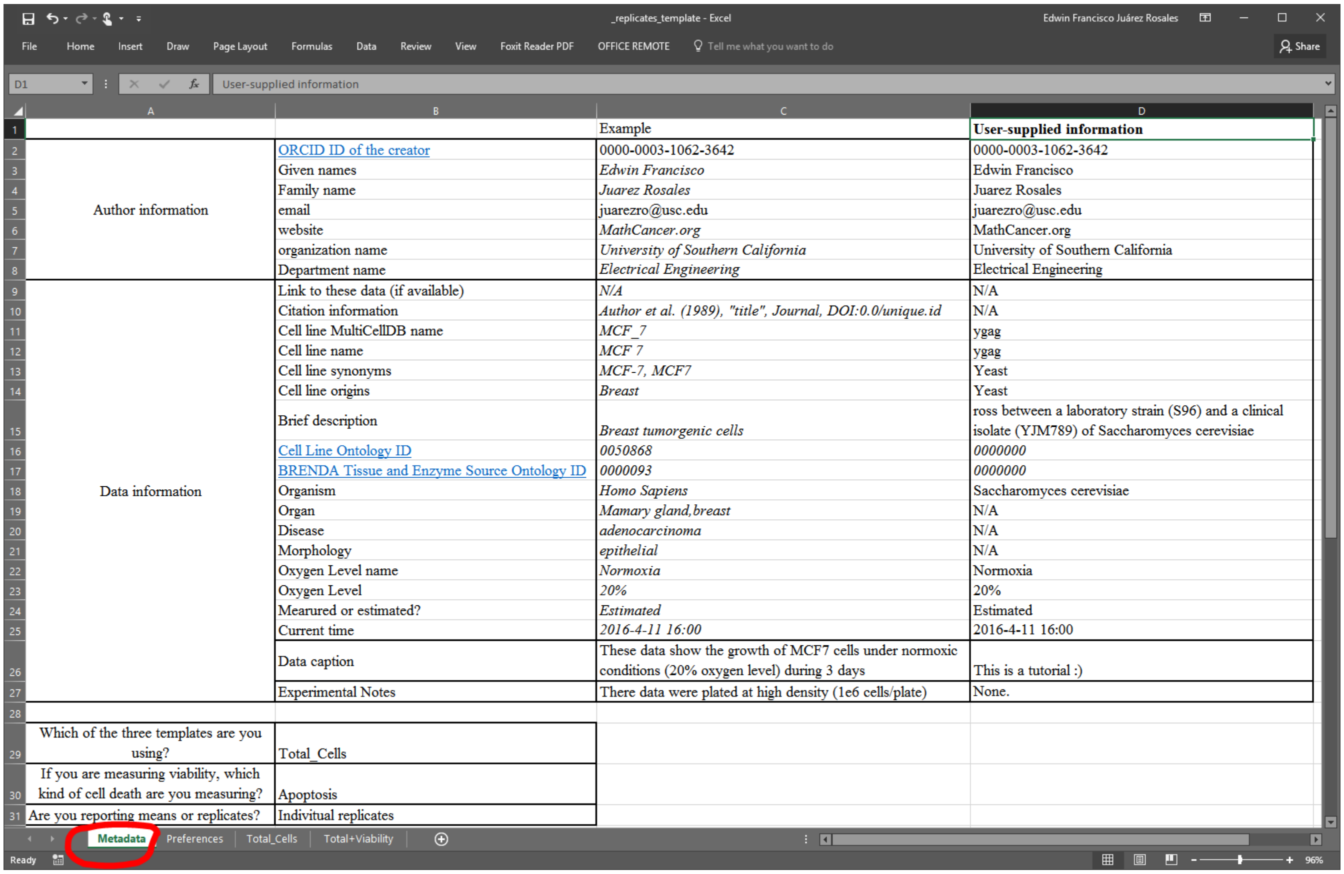

	A	B	C	D
1			Example	**User-supplied information**
2	Author information	ORCID ID of the creator	0000-0003-1062-3642	0000-0003-1062-3642
3		Given names	*Edwin Francisco*	Edwin Francisco
4		Family name	*Juarez Rosales*	Juarez Rosales
5		email	juarezro@usc.edu	juarezro@usc.edu
6		website	*MathCancer.org*	MathCancer.org
7		organization name	*University of Southern California*	University of Southern California
8		Department name	*Electrical Engineering*	Electrical Engineering
9	Data information	Link to these data (if available)	*N/A*	N/A
10		Citation information	*Author et al. (1989), "title", Journal, DOI:0.0/unique.id*	N/A
11		Cell line MultiCellDB name	*MCF_7*	ygag
12		Cell line name	*MCF 7*	ygag
13		Cell line synonyms	*MCF-7, MCF7*	Yeast
14		Cell line origins	*Breast*	Yeast
15		Brief description	*Breast tumorgenic cells*	ross between a laboratory strain (S96) and a clinical isolate (YJM789) of Saccharomyces cerevisiae
16		Cell Line Ontology ID	*0050868*	*0000000*
17		BRENDA Tissue and Enzyme Source Ontology ID	*0000093*	*0000000*
18		Organism	*Homo Sapiens*	Saccharomyces cerevisiae
19		Organ	*Mamary gland,breast*	N/A
20		Disease	*adenocarcinoma*	N/A
21		Morphology	*epithelial*	N/A
22		Oxygen Level name	*Normoxia*	Normoxia
23		Oxygen Level	*20%*	20%
24		Mearured or estimated?	*Estimated*	Estimated
25		Current time	*2016-4-11 16:00*	2016-4-11 16:00
26		Data caption	These data show the growth of MCF7 cells under normoxic conditions (20% oxygen level) during 3 days	This is a tutorial :)
27		Experimental Notes	There data were plated at high density (1e6 cells/plate)	None.
28				
29	Which of the three templates are you using?	Total_Cells		
30	If you are measuring viability, which kind of cell death are you measuring?	Apoptosis		
31	Are you reporting means or replicates?	Indivitual replicates		

Figure 7: Example of the input metadata for CellPD.

After making those modifications, we are saving the file as `input_s1.xlsx`

2- Running CellPD

2.1- Running CellPD in a Windows environment (without installing Python 3)

Make sure that `runCellPD.bat`, `CellPD.py`, the input file (in this example we named it `input_s1.xlsx`), and the folder named `files` are all in the same directory.

Double-click on `runCellPD.bat` and, when prompted, type the name of the input file when CellPD asks for it (in this example we would type `input_s1`)

2.2-Running the Python script on a Windows + Python 3 environment

Make sure that CellPD.py, the input file (in this example we named it `input_s1.xlsx`), and the folder named files are all in the same directory.
1- Open the command line.
`shift + right click` on the current folder, then select `Open command window here`

2- Once cmd opens, type
Python CellPD.py --name input_s1
If we omit the optional argument (the name of the input file), CellPD will simply ask for an input name before proceeding. Note that it is fine to leave out the extension of the input file (e.g., type `input_s1` instead of `input_s1.xlsx`); CellPD checks for the XLSX or XLS file whether or not we type the extension.

2.3-Running CellPD on a mac (OSX) environment (without installing Python 3)

Make sure that `runCellPD`, `CellPD27.py`, the input file (in this example we named it `input_s1.xlsx`), and the folder named `files` are all in the same directory.

Double-click on `runCellPD` and, when prompted, type the name of the input file when CellPD asks for it (in this example we would type `input_s1`).

2.4-Running the Python script on a Unix (Mac or Linux) + Python 3 environment

Make sure that CellPD.py, the input file (in this example we named it `input_s1.xlsx`), and the folder named files are all in the same directory.
1- Open the terminal

2- Once the terminal opens, navigate to the directory where CellPD.py is located
3- type `Python3 CellPD.py input_s1`

If we omit the optional argument (the name of the input file), CellPD will simply ask for an input name before proceeding. Note that it is fine to leave out the extension of the input file (e.g., type `input_s1` instead of `input_s1.xlsx`); CellPD checks for the XLSX or XLS file whether or not we type the extension.

3- CellPD outputs

CellPD will create a directory with the name specified in the `Preferences` tab. In this directory(which we named Tutorial earlier) you will find the following files and folders:

Key: Text in square brackets "[]" are descriptions of what that file or folder is. Files or folder names in between curly brackets "{}" are a generic description of what that name would be. For example, this tutorial would be described as:
{tutorial}.docx [This is the tutorial for CellPD]

- Tutorial [This is the parent directory]
    - overall_report.html [the local HTML parameter estimation report]
    - output\ [a folder with CellPD's secondary outputs]
        - data_summary.csv
        - data_summary.txt
        - {digital_cell_line}.xml
        - mape_ranking.csv
        - rcs_ranking.csv
        - style.css
        - data\
            - all_models.jpg [A plot of all of the fitted models]
            - all_models.png [A plot of all of the fitted models]
            - all_models.svg [A plot of all of the fitted models]
            - all_models.tiff [A plot of all of the fitted models]
            - data.jpg [A plot of the provided data]
            - data.p [This is a "pickle" file to store the data in a pythonic way]
            - data.png [A plot of the provided data]
            - data.svg [A plot of the provided data]
            - data.tiff [A plot of the provided data]
            - data_BW.jpg [A plot of the provided data in black and white]
            - data_BW.png [A plot of the provided data in black and white]
            - data_BW.svg [A plot of the provided data in black and white]
            - data_BW.tiff [A plot of the provided data in black and white]
            - data_loglinear.jpg [A log-y linear-x plot of the provided data]
            - data_loglinear.png [A log-y linear-x plot of the provided data]
            - data_loglinear.svg [A log-y linear-x plot of the provided data]
            - data_loglinear.tiff [A log-y linear-x plot of the provided data]
            - data_loglinear_BW.jpg [A log-y linear-x plot of the provided data in black and white]
            - data_loglinear_BW.png [A log-y linear-x plot of the provided data in black and white]
            - data_loglinear_BW.svg [A log-y linear-x plot of the provided data in black and white]
            - data_loglinear_BW.tiff [A log-y linear-x plot of the provided data in black and white]
        - {math model}\
            - {math model}.jpg [A plot of the model fit]
            - {math model}.png [A plot of the model fit]
            - {math model}.svg [A plot of the model fit]
            - {math model}.tiff [A plot of the model fit]
            - {math model}_BW.jpg [A black and white plot of the model fit]
            - {math model}_BW.png [A black and white plot of the model fit]
            - {math model}_BW.svg [A black and white plot of the model fit]
            - {math model}_BW.tiff [A black and white plot of the model fit]
            - {math model}_caption.txt [A text file with the plot caption]

{math model}_description.txt [A text file with the model description]
{math model}_eqn.png [An image showing the model equation(s)]
{math model}_eqn.svg [An image showing the model equation(s)]
{math model}_eqn.tex [A standalone LaTeX file with the model equation(s)]
{math model}_latex_title.txt [A text file with the name of the model in LaTeX format]
{math model}_loglinear.jpg [A log-y linear-x plot of the model fit]
{math model}_loglinear.png [A log-y linear-x plot of the model fit]
{math model}_loglinear.svg [A log-y linear-x plot of the model fit]
{math model}_loglinear.tiff [A log-y linear-x plot of the model fit]
{math model}_loglinear_BW.jpg [A black and white log-y linear-x plot of the model fit]
{math model}_loglinear_BW.png [A black and white log-y linear-x plot of the model fit]
{math model}_loglinear_BW.svg [A black and white log-y linear-x plot of the model fit]
{math model}_loglinear_BW.tiff [A black and white log-y linear-x plot of the model fit]
{math model}_MCDS_template.txt [A python template of model in MultiCellDS format]
{math model}_model.tex [The model equation(s) in latex format]
{math model}_output.p [A pickle file to store the model output in a pythonic way]
{math model}_parameters.xlsx [A spreadsheet with the model 's parameter estimates]
{math model}_partial.xml [The MultiCellDS representation of the model parameters]
{math model}_plane.png [An image with the equations of the model, not enumerated]
{math model}_plane.svg [An image with the equations of the model, not enumerated]
{math model}_plane.tex [The equations of the model, not enumerated in LaTeX]
{math model}_report.html [The HTML file with the model's parameter estimates]
{math model}_table.csv [A CSV file with the model's parameter estimates]
{math model}_table.tex [A table with the model's parameter estimates in LaTeX]
{math model}_table.txt [A text file with the model's parameter estimates]
{math model}_table_caption.txt [The model's parameter estimates table capion]
{math model}_table_html.txt [A table with the model's parameter estimates in HTML]
{math model}_title.txt [The name of the model]

References:

GAGNEUR, J., STEGLE, O., ZHU, C., JAKOB, P., TEKKEDIL, M. M., AIYAR, R. S., SCHUON, A.-K., PE'ER, D. & STEINMETZ, L. M. 2013. Genotype-environment interactions reveal causal pathways that mediate genetic effects on phenotype. *PLoS Genet,* 9**,** e1003803.
