# Supplementary material for: Quantifying differences in cell line population dynamics using CellPD
Source: BMC Syst Biol. 2016 Sep 21;10:92. doi: 10.1186/s12918-016-0337-5 (PMC5031291; doi:10.1186/s12918-016-0337-5)
Supplement: Additional file 8: — Cuantificación de Dinámicas Poblacionales de Cultivos de Líneas Celulares Utilizando CellPD. This file is a Spanish summary of the manuscript. (PDF 127 kb) [file 12918_2016_337_MOESM8_ESM.pdf]

Cuantificación de Dinámicas Poblacionales de Cultivos de Líneas Celulares Utilizando CellPD

Additional file 5 for "*Quantifying Differences in Cell Line Population Dynamics Using CellPD*"

Authors: Edwin F. Juarez[1,2], Roy Lau[1], Samuel H. Friedman[1], Ahmadreza Ghaffarizadeh[1], Edmond Jonckheere[2], David B. Agus[1], Shannon M. Mumenthaler[1], and Paul Macklin[1].

Authors' affiliations:
1: Lawrence J. Ellison Institute for Transformative Medicine, University of Southern California.
2: Department of Electrical Engineering, Viterbi School of Engineering, University of Southern California.

Contact information: Paul Macklin: Paul.Macklin@usc.edu
Edwin F. Juarez: juarezro@usc.edu

Marco teórico

El rápido desarrollo de la Biología Sistemática (o Biología de Sistemas) ha generado una abundancia de bases de datos, entre ellas destacan las bases de datos de alto rendimiento de cultivos celulares. Esta abundancia de información ha puesto de manifiesto la necesidad de contar con herramientas para procesar y analizar dichas bases de datos, con el objetivo de comparar cuantitativamente cambios moleculares con el fenotipo celular. Es necesario que los resultados producidos por estas herramientas sean reproducibles y accesibles a la comunidad científica. Sin embargo, las herramientas existentes para el análisis cuantitativo suelen requerir conocimientos especializados para ser usadas. CellPD (digitalizador del fenotipo celular, por sus siglas en inglés) facilita el análisis fenotípico cuantitativo y permite a sus usuarios utilizar modelos matemáticos que describen dinámicas poblacionales de cultivos celulares sin la necesidad de conocimiento especializado. Los datos de entrada de CellPD son almacenados en un solo archivo (una hoja de cálculos), pero es capaz de generar una variedad de archivos; entre los cuales se destacan reportes de estimación de parámetros (en formato XLSX, CSV, y TXT), gráficos de alta calidad y archivos de formato XML que facilitan el uso de técnicas de minería de datos.

Descargar CellPD

CellPD es un programa de código abierto (licencia MIT) escrito en Python, que puede ser descargado en CellPD.sf.net o en MultiCellDS.org/CellPD. De momento el programa y los tutoriales se encuentran en inglés. Adicionalmente, preguntas sobre CellPD pueden ser dirigidas (en español o en inglés) al primer autor de este artículo al correo electrónico: juarezro@usc.edu.

Resultados

Para validar las estimaciones realizadas con CellPD, se comparó con otra herramienta científica pública, llamada cellGrowth, y con las herramientas de regresión de Microsoft Excel. CellPD estima correctamente las tazas de crecimiento de cultivos de levadura comparado con cellGrowth y Excel. A la vez, CellPD es más robusta a la escasez de datos. También se examinó la facilidad de uso de CellPD con miembros de un grupo de biólogos sin entrenamiento en modelamiento computacional, de los cuales todos fueron capaces de usar CellPD y estimar las tazas de cambio de un cultivo celular en menos de 30 minutos. Se diseñó un experimento simulado con el objetivo de demostrar cómo CellPD puede ayudar en el análisis de bases de datos de alto rendimiento. Dicha simulación crea un archivo idéntico a los creados por microscopios de alto contenido (HCS, por sus siglas en inglés). En el experimento simulado, una línea celular es expuesta a 5 concentraciones de dos fármacos. CellPD estima correctamente las tazas de nacimiento, muerte, y crecimiento de las

células. Además, permite cuantificar los efectos citostáticos y citotóxicos de los fármacos simulados, distinguiendo correctamente entre ellos.

Conclusión

CellPD es una herramienta que puede ser utilizada por científicos (con o sin entrenamiento en modelamiento computacional/matemático) para cuantificar aspectos importantes del fenotipo celular. Algunos usos de CellPD incluyen cuantificación de efectos de fármacos en cultivos celulares, análisis funcional de experimentos de eliminación de genes, control de la calidad de la información recolectada en experimentos, generación de *big data* para minería de datos, e integración de información de experimentos biológicos en modelos computacionales.
