# Supplementary material for: Quantifying differences in cell line population dynamics using CellPD
Source: BMC Syst Biol. 2016 Sep 21;10:92. doi: 10.1186/s12918-016-0337-5 (PMC5031291; doi:10.1186/s12918-016-0337-5)
Supplement: Additional file 10: — Windows and OSX distributions of CellPD. This file contains simple instructions and links to download CellPD and use it on a Windows or an OSX computer. (PDF 119 kb) [file 12918_2016_337_MOESM10_ESM.pdf]

Windows and OSX distributions of CellPD

Additional file 5 for "*Quantifying Differences in Cell Line Population Dynamics Using CellPD*"

Authors: Edwin F. Juarez[1,2], Roy Lau[1], Samuel H. Friedman[1], Ahmadreza Ghaffarizadeh[1], Edmond Jonckheere[2], David B. Agus[1], Shannon M. Mumenthaler[1], and Paul Macklin[1].

Authors' affiliations:
1: Lawrence J. Ellison Institute for Transformative Medicine, University of Southern California.
2: Department of Electrical Engineering, Viterbi School of Engineering, University of Southern California.

Contact information: Paul Macklin: Paul.Macklin@usc.edu
Edwin F. Juarez: juarezro@usc.edu

We created a Windows stand-alone version of CellPD using PyInstaller (as described in the main text). This distribution of CellPD does not require installation of any software and can be downloaded (along with its tutorial) from CellPD.sf.net at:
https://sourceforge.net/projects/cellpd/files/CellPD/1.0.0/Windows/

We also created an OSX stand-alone version of CellPD. This distribution of CellPD leverages the pre-installed version of Python 2.7 which comes with OS 10.7 and up. This distribution of CellPD (and its tutorial) can be downloaded from CellPD.sf.net at:
https://sourceforge.net/projects/cellpd/files/CellPD/1.0.0/OSX/
