# Supplementary material for: Quantifying differences in cell line population dynamics using CellPD
Source: BMC Syst Biol. 2016 Sep 21;10:92. doi: 10.1186/s12918-016-0337-5 (PMC5031291; doi:10.1186/s12918-016-0337-5)
Supplement: Additional file 1: — Example of CellPD’s outputs. This folder contains two examples of the outputs generated by CellPD (using the data from Fig. 2 and Additional file 6). (ZIP 36462 kb) [file 12918_2016_337_MOESM1_ESM.zip › USC/output/CellPD_output.zip!/USC/output/CellPD_output.zip!/USC/overall_report.html]

HCT116: CellPD Results


# CellPD Results

## HCT116 cell line

These are the main results from running the CellPD: Cell Line Phenotype Digitizer (Version 1.0). Please cite this tool as:

> We fit the data to several growth models using CellPD (Version 1.0), which uses least-squares fitting ... (Juarez et al., 2015).
>
> E. Juarez et al., *CellPD: a cell line parameter digitizer to quantify and record critical cell line growth parameters*, eLife (2016, in review)

**[Download Digital Cell Line]**

**[Download these reports as a standalone zip file]**

## User-supplied data

Here are the data, as given by Edwin Francisco Juarez Rosales on 2016-04-15:

|  | Total cells | |
| --- | --- | --- |
| Hours | Mean | STD |
| 0.0 | 1.13e+03 | 53 |
| 48.0 | 5.51e+03 | 755 |
| 72.0 | 1.55e+04 | 1.67e+03 |

**Fig. 1:** None.  
[Download as PNG]
[Download as SVG]  
[Download as PNG (black & white)]
[Download as SVG (black & white)]

### Metadata

These data were provided by:

|  |  |
| --- | --- |
| Given names | Edwin Francisco |
| Family name | Juarez Rosales |
| Email | juarezro@usc.edu |
| Website | MathCancer.org |
| Organization | University of Southern California |
| Department | Electrical Engineering |
| ORCID | 0000-0003-1062-3642 |

Cell line information:

|  |  |
| --- | --- |
| Link to data | http://physics.cancer.gov/docs/bioresource/colorectal/NCI-PBCF-CCL247\_HCT116\_SOP-508.pdf |
| Citation information | http://physics.cancer.gov/docs/bioresource/colorectal/NCI-PBCF-CCL247\_HCT116\_SOP-508.pdf NCI PS-ON, "Thawing, Propagating, and Cryopreserving Protocol: HCT116, Version 1.6." NCI-PBCF-CCL247 (HCT 116). http://physics.cancer.gov/docs/bioresource/colorectal/NCI-PBCF-CCL247\_HCT116\_SOP-508.pdf. |
| MultiCellDB ID | HCT\_116\_0-4 |
| Cell line name | HCT116 |
| Synonyms | HCT116 |
| Cell line origins | Colon |
| Brief description | Colorectal Carcinoma |
| CLO ID | 1582 |
| BTO ID | 2806 |
| Organism | Homo Sapiens |
| Organ | Colon |
| Disease | colorectal adenocarcinoma |
| Morphology | N/A |
| oxygenation level name | Normoxia |
| oxygenation level | 0.2 |
| oxygenation Measurement Type | Estimated |
| Time of creation | 2016-04-15T11:19:03.516547-07:00 |

---

## Models at a glance

Here is a summary of the model fitting results. Click any model for its fitted parameters and plots.

Ranked by Mean Absolute Percentage Error (MAPE)

[Lower MAPE is better.]

Warning legend: c = high correlation, u = high uncertainty, e = error estimates missing

| Name | MAPE | Warning |
| --- | --- | --- |
| Total cells model | 6.37% |  |
| Total cells model (with logistic limits) | 6.37% | (u) |

Ranked by Reduced Chi Squared Goodness of Fit (Χ^2^_ν_)

[Lower Χ^2^_ν_ is better.]

Warning legend: c = high correlation, u = high uncertainty, e = error estimates missing

| Name | Χ^2^_ν_ | Warning |
| --- | --- | --- |
| Total cells model | 4.64 |  |
| Total cells model (with logistic limits) | inf | (u) |

**Fig. 1:** None.  
[Download as PNG]
[Download as SVG]

---

[Back to top]

CellPD: Cell Line Phenotype Digitizer (Version 1.0) - Results

[Back to the main page]
