# Supplementary material for: Quantifying differences in cell line population dynamics using CellPD
Source: BMC Syst Biol. 2016 Sep 21;10:92. doi: 10.1186/s12918-016-0337-5 (PMC5031291; doi:10.1186/s12918-016-0337-5)
Supplement: Additional file 1: — Example of CellPD’s outputs. This folder contains two examples of the outputs generated by CellPD (using the data from Fig. 2 and Additional file 6). (ZIP 36462 kb) [file 12918_2016_337_MOESM1_ESM.zip › USC/output/CellPD_output.zip!/USC/output/CellPD_output.zip!/USC/output/data/Data_loglinear_BW.pdf]

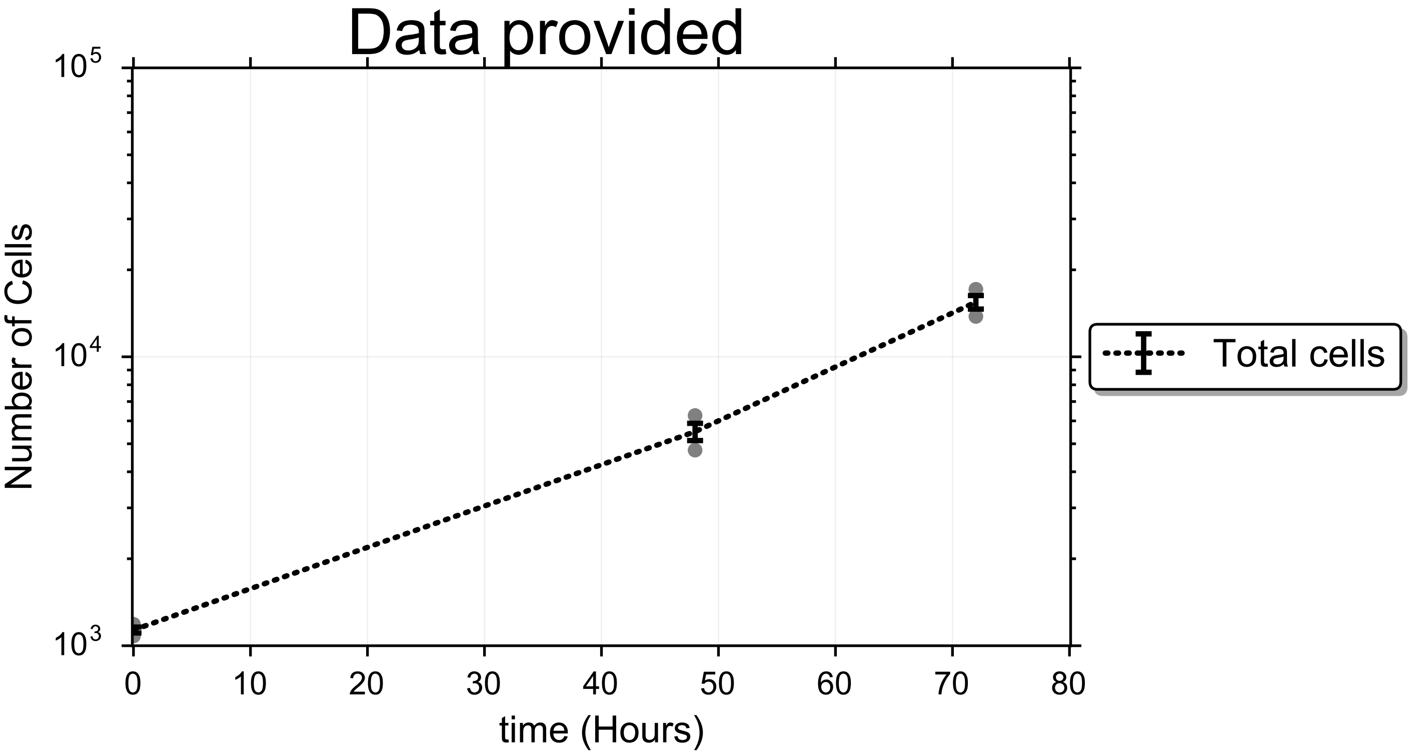

Data provided
Number of Cells
10^5
10^4
10^3
0
10
20
30
40
50
60
70
80
time (Hours)
Total cells
